# The landscape of enhancer RNA identify prognosis‐related molecular subtypes in gastric cancer

**DOI:** 10.1002/cam4.4959

**Published:** 2022-07-08

**Authors:** Aiting Yan, Ying Chen, Rongrong Bian, Cuizhu Wang, Haitao Que, Yucheng Shen, Xiaomin Lu

**Affiliations:** ^1^ Department of Oncology Affiliated Haian Hospital of Nantong University Nantong Jiangsu China; ^2^ Department of Oncology The Yancheng School of Clinical Medicine of Nanjing Medical University, Yancheng Third people's hospital Yancheng Jiangsu China; ^3^ Department of Oncology, Nanjing Liuhe People Hospital Nanjing Jiangsu China

**Keywords:** eRNAs, gastric cancer, immune microenvironment, methylation, mutation

## Abstract

**Background:**

Enhancer RNAs (eRNAs), the transcriptional products of active enhancers, are of great significance in the initial progression of cancers. However, the biological function and bioinformatics profiles of eRNA in gastric cancer remains largely enigmatic.

**Methods:**

Firstly, STAD were clustered into three subtypes with the data of eRNA expression from TCeA. Then we explored the difference of the tumor immune microenvironment, transcription levels, and transcription regulation among the three clusters. Finally, samples collected from 12 patients diagnosed with STAD were used to conduct qRT‐PCR, verifying the conclusion based on network database.

**Results:**

The three clusters were detected to have different tumor microenvironments: Cluster A has an immune “cold” microenvironment. While cluster B features as more infiltration of immune cells, accompanied with higher expression of immune checkpoints such as PDCD1, LAG3, and TIGIT. Besides, Cluster C shows a higher stromal feature with B lineage, neutrophils, and fibroblasts. Further analyses indicated that CpG island methylation level of Cluster B is different from the other two clusters. Meanwhile, Cluster A and B showed significant enrichment of TP53 and KRAS mutation respectively while Cluster C has higher tumor mutation burden (TMB) and microsatellite instability (MSI). With the elaboration of transcriptional regulation of epigenetic clustering, we detected that Cluster A enriched in epithelial phenotype pathways. Cluster B enriched in cell–cell adhesion. Cluster C enriched in fibroblast proliferation. The clinical cohort show that Cluster B patients have lower interstitial cell characteristics and CAF infiltration.

**Conclusion:**

We identified three unique epigenetic clusters of STAD through the differential activation of super‐enhancers, and identified Cluster B with a higher immune infiltrating and a better prognosis, which provides a novel understanding of eRNAs and potential clinical applicability of eRNA‐based molecular subtypes in gastric cancer.

## Graphical abstract

We explored the landscape of eRNA and identified three subtypes with different immune status, genetic alternation, and epigenetic clusters, which provided eRNA‐based prognostic biomarkers and novel understanding in molecular carcinogenesis.

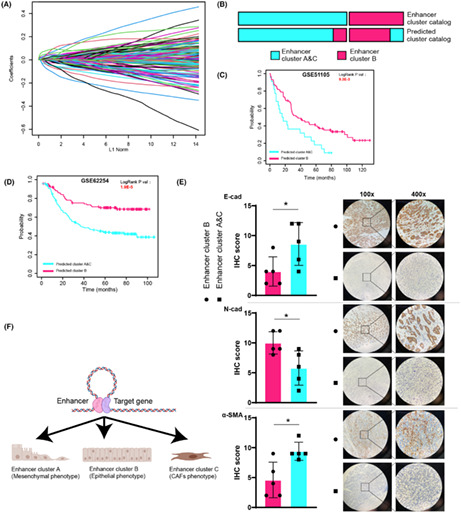

## 1 INTRODUCTION

The incidence of gastric cancer has been rising globally, and gastric cancer remains a principle cause of cancer‐related death^1^ and represents a major worldwide public health problem.^2^ Gastric adenocarcinoma is major type of gastric cancer. Responsible for more than 90% of cases,^2^ they are detected in advanced stages in most situation.^3^ Because of the lack of accurate bio‐marker guidance, it is difficult to choose individualized treatment plan in clinic. Therefore, the five‐year survival rate of patients with advanced STAD reveals a low level. It is important to identify sensitive therapeutic targets for individual gastric adenocarcinoma patients and to perform precise stratified management of gastric adenocarcinoma patients .The study of cistromics revealed that the interaction between protein‐chromatin and inter‐chromatin at the DNA level is based on a variety of high‐throughput sequencing technologies,^4^, ^5^, ^6^ especially the powerful transcriptional regulation ability of enhancer. Enhancers are key non‐coding DNA sequences as a cis‐element,^7^ which control temporal or spatial specific gene expression characteristics during various biological processes and diseases, especially in cancers.^8^, ^9^, ^10^, ^11^ Transcription factors surrounding enhancers can establish nucleosome‐adjacent, conserved, covalent chromatin modifications by triggering the recruitment of chromatin‐modifying enzymes.^12^, ^13^ Different from promoters, enhancers are usually thousands to millions of bases away from the genes they regulate target genes, non‐directionally.^14^ However, due to stringent requirements for tumor tissue samples, unlike RNA‐seq, histone ChIP‐seq or CUT&RUN‐seq cannot be easily applied to large cohorts of tumor samples, such as TCGA, thereby limiting the power of connecting enhancers activation with clinical phenotypes.^15^ However, surprising studies found that enhancer regions are actively transcribed via recruit RNA polymerases, and generate enhancer RNAs (eRNAs).^16^, ^17^

Considering the conservation of enhancers loci and transcription process of eRNA, they can be regarded as markers of active enhancers. eRNA transcription will identify enhancers that are caught when regulating gene expression. For example, published studies discovered that eRNA functioned as a better marker of active enhancers than H3K27ac.^18^ That eRNA marking active enhancers in cells is quite a surprising finding, and several studies have reported the successful orientation of active enhancer regions with the help of eRNA.^19^ eRNA expression profiles are characterized by high tissue‐specification,^20^ and accordingly useful to describe specific disease states and identify disease‐specific variants in a wide scope of diseases.^15^

The aim of this study was to seek for novel therapeutic gastric cancer bio‐marker. Recently, Chen et al provided a high‐resolution map for eRNA which can conveniently quantified super‐enhancer activities via RNA‐seq.^15^ This allows us to use a large amount of publicly available RNA‐seq data to explore the heterogeneity of STAD in enhancer reprogramming. We classified STAD into three subgroups based on the different expression of eRNA. The three subgroups differ in the aspects of immune microenvironment, methylation data analysis, multiple omics analysis, transcriptional regulation, etc., and among the three clusters, we discovered promising one with better reaction to immunotherapy and better prognosis.

## 2 METHODS

### 2.1 Data download

We downloaded the expression data of TCGA‐LUAD eRNAs in putative super‐enhancers (*n* = ~200 k) from TCeA—The Cancer RNA Atlas (https://bioinformatics.mdanderson.org); We downloaded the clinical data, DNA methylation(450 K) data and RNA‐seq data of TCGA‐STAD from UCSC Xena browser (http://xena.ucsc.edu). The expression matrix of 22 immune cells was obtained from the CIBERSORT algorithm (https://cibersortx.stanford.edu).

### 2.2 Unconscious cluster arrays and different expression analysis

Comparison of eRNA expression between early and advanced gastric cancer patient.The difference of each eRNA expression between 40 tumor IV patients and 16 tumor IA patients adenocarcinomas was evaluated. FPKM was counted to measure eRNA expression. The fold change was calculated and transformed to log2 for the quantification of gene expression value from advanced patients to early patients, the eRNA of which elevated significantly(log2FC > 1.5, *p* value < 0.05). Then, consensus clustering was carried out with the R package ConsensusClusterPlus (v 1.54.0). For each sample, the input data was the Standardized expression value (FPKM) for differential eRNAs defined above.The consensus clustering was conducted by the following parameters: Number of repetitions = 50; pItem = 0.8; pFeature = 1; Pearson distance metric and HCLUST.

### 2.3 Whole exon sequence data and methylation data analysis

We used R package ESTIMATE (v 1.0.13) to analyze the immune infiltration score, we used the RNA‐seq data to calculate the infiltration score. We used R package ChAMP(v 2.20.1) to analyze DNA methylation difference among three enhancer cluster; R package impute(v 1.64.0) was used to remove the Na value in the methylation matrix. We calculated the average methylation level of each site in each enhancer cluster, and drew the distribution curve of methylation signal value density.

### 2.4 Differential expression genes analysis and constructing epigenetic clusters signature

Between the two experimental conditions, differential expression analysis was conducted using “DESeq2” R package (Version 1.26.0) with standard comparison mode. The log2|fold change| > 1 and adj. *p* < 0.05 were established as the cut‐off values to search differentially expressed genes (DEGs) between each cluster. A LASSO algorithm regression model for the selection of candidate genes was carried out with penalty parameter tuning conducted by 10‐fold cross‐validation, using glmnet package in R(Version 3.0). The cut‐off value of each Covariate of candidate genes is an absolute value of 1.5.

### 2.5 RNA extraction and quantitative reverse‐transcription polymerase chain reaction

Twelve patients who were subject to surgery without neoadjuvant chemotherapy (NAC) and were diagnosed with STAD at Affiliated Haian Hospital of Nantong University. The study was permitted by the Regional Ethics Committee at Affiliated Haian Hospital of Nantong University. The experiments were conducted with the understanding and written consent of each participant. The study methodologies conformed to the standards set by the Declaration of Helsinki. Study material consisted of formalin‐fixed paraffin‐embedded (FFPE) specimens obtained from radical surgery from 2017 to 2020. Each patient were subjected to a standard radical surgical procedure, and all specimens were analyzed by expert pathologists. Total RNA was extracted from FFPE slides using RNeasy FFPE Kit (Qiagen). The synthesis of complementary DNA (cDNA) was carried out by PrimeScript RT Master Mix (RR036A) (TAKARA). We conducted the quantitative reverse‐transcriptase polymerase chain reaction (qRT‐PCR) assays by ViiA 7 Dx RT‐PCR System (Applied Biosystems) with PowerUp SYBR Green Master Mix (Applied Biosystems). The cycling conditions and procedure were followed as 40 cycles of 95°C for 15 s and 60°C for 60 s. The relative expression of target genes (2^−ΔCT^) was normalized against GAPDH reference gene.

### 2.6 Immunohistochemistry (IHC)

STAD tissues were fixed with 10% formalin and embedded in paraffin, which were cut into 4‐μm‐thick sections and incubated overnight with primary antibodies, including: Anti‐E‐Cadherin (Abcam, #ab1416), anti‐N‐Cadherin (Abcam, #ab76011), and anti‐αSMA (Abcam, #ab7817). The sections were further incubated with a secondary antibody (ProteinTech, #SA00003‐1 & #SA00003‐2) at 37°C for 1.5 h and stained with a 3,3‐diaminobenzidine solution.

### 2.7 ChIP‐seq data download and visualization

ChIP‐seq data were download from ENCODE (https://www.encodeproject.org/, H3K27ac ChIP‐seq: ENCLB776PUV. EP300 ChIP‐seq: ENCLB929JHE). ChIP‐seq visualization used Integrative Genomics Viewer (IGV).^21^

### 2.8 Statistical analysis and schematic diagram

All statistical analyses were carried out using R (Version 3.6.2) and the programs GraphPad Prism 8. We used the Student's *t*‐test in the limma R package to obtain *p* values when dealing with high‐throughput data variance analysis. To deal with other non‐normally distributed data, we used the nonparametric *t*‐test. we used the most widely used Log‐Rank *p*‐value for patient survival analysis. All statistical tests were two‐tailed and the statistical significance level set at 0.05 in this study. Schematic diagram created with BioRender.com.

## 3 RESULT

### 3.1 Epigenetics clusters in gastric carcinoma

**FIGURE 1:**
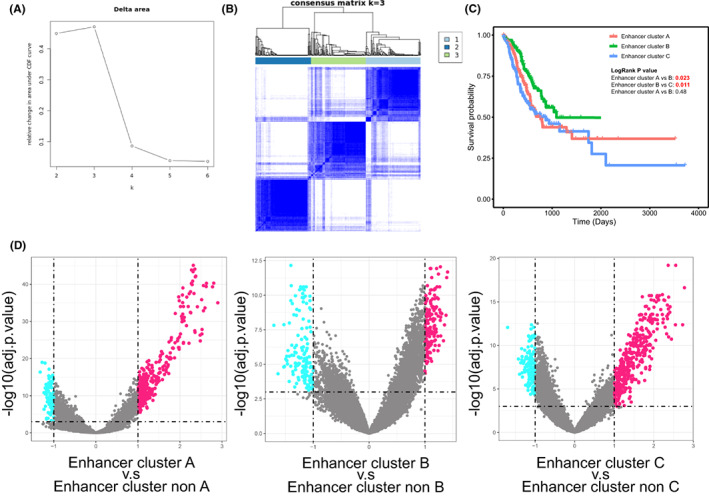
(A) The Delta area of several clustering numbers via un‐consensus clustering method. (B) The consensus matrix of gene expression when clustering number is 3. (C) The Kaplan–Meier survival array of three epigenetic clusters. (D) The differently expression analysis of eRNA in three epigenetic cluster.

Previous studies have expounded the regulation of epigenetic reprogram on the progression of malignant tumors from multiple perspectives.^22^ Epigenetic therapy has become a target for cancer therapy.^23^ Based on the expression of eRNA in TCGA database, we performed clustering of gastric cancer patients from the perspective of epigenetic heterogeneity. Using unconscious cluster array, we clustered STAD patients base on eRNA expression via appropriate k means (Figure 1A). The three clusters (Cluster A, B & C, which includes 138, 140, and 137 STAD patients, respectively) had different eRNA expression characteristics (Figure 1B) and overall survival (Figure 1C), that Cluster B h. Moreover, we identified enhancers of cluster‐specific activation for each cluster for subsequent analysis (Figure 1D).

### 3.2 Multiple omics analysis of epigenetic clusters

**FIGURE 2:**
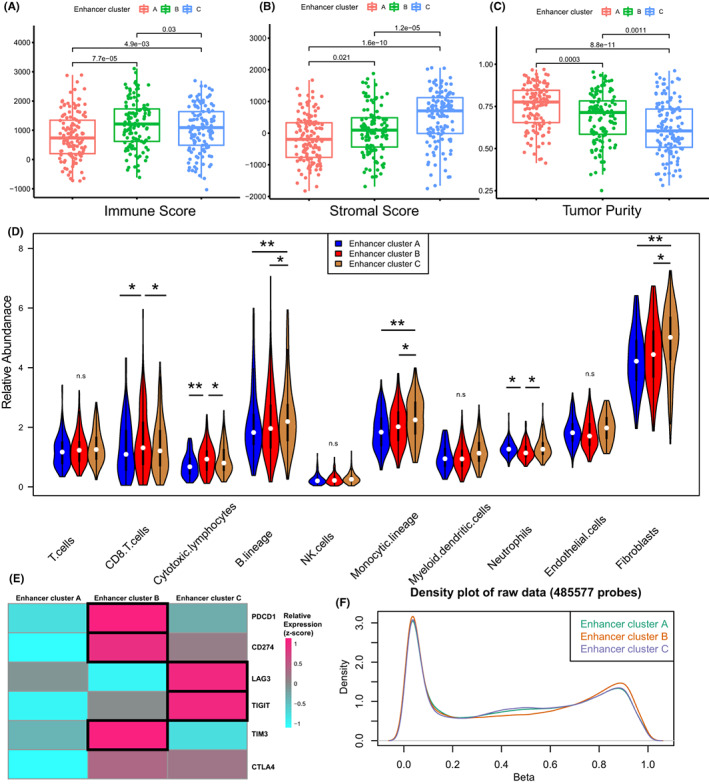
(A–C) The immune score, stromal score, and tumor purity of three epigenetic clusters via ESTIMATE algorithm. (D) The tumor environment cells infiltration of three epigenetic clusters via CIBERSORTS algorithm. (E) The expression of immune checkpoints of three epigenetic clusters. (F) The landscape of 450 K DNA methylation panel array of three epigenetic clusters via Champ algorithm.

We further describe the differences among the three epigenetic clusters from the perspective of multi‐omics. Firstly, we analyzed the immune infiltration of three clusters of bulk samples. Using ESTIMATE immune infiltration tools, we assessed several composition scores of three epigenetic clusters. Remarkably, each of the three clusters had unique tumor microenvironments. Cluster A has a significant high tumor purity (Figure 2C) which suggested an immune “cold” microenvironment. Cluster B has a significant high Immune Score (Figure 2A) while Cluster C has a higher Stromal Score (Figure 2B). The CIBERSORT assay verified the above results, while Cluster B has more infiltration abundance of CD8+ T cells and cytotoxic lymphocytes, consist with the immune activated status (Figure 2D). This immune “hot” status may contribute the better outcome of patients in this cluster. Cluster C has more infiltration abundance of B lineage, monocytic lineage, neutrophils, and fibroblasts, which consist with a niche of tumor progression.

The differences in the composition of lymphocytes and stromal cells between the three clusters allow us to continue to explore the differences in the expression levels of immune checkpoints, which indicate that lymphocytes infiltrated and function exhaust. Cluster B has higher expression of PDCD1, CD274, and TIM3, suggesting that CD8+ T cell function is exhausted, which is consistent with the results of immune infiltration (Figure 2E). At the same time, Cluster C has higher expression of LAG3 and TIGIT, suggesting that while some cytotoxic lymphocytes are exhausted, there is also exhaustion of antigen presenting ability. We have a landscape of the three cluster methylation sites at the same time (Figure 2F), and we found that CpG island methylation level of Cluster B is significantly different from the other two clusters, which may be due to the abnormal methylation of multiple sites caused by enhancer hijacking between clusters.

**FIGURE 3:**
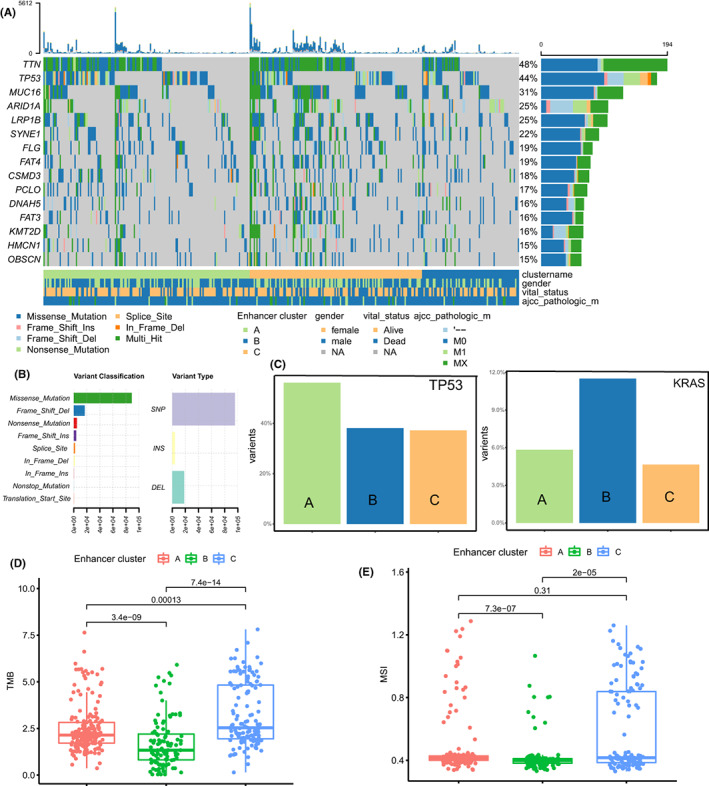
(A) The landscape of genetic mutation events of three epigenetic clusters. (B) The mutation classification and variant type of all TCGA‐STAD patients. (C) The percentage of TP53 and KRAS mutation patients in three epigenetic clusters. (D, E) The tumor mutation burden (TMB) and Microsatellite instability (MSI) of three epigenetic clusters.

To fully understand the differences in transcription levels between epigenetic clusters, we elaborated on three clustered genomic events. Figure 3A,B reveal the landscape of genomic mutation events, that missense mutation is the most high‐frequency mutation classification and SNP is most high‐frequency variant type. Analysis of several cancer‐promoting mutations that are often used as therapeutic targets. Cluster A and Cluster B showed significant enrichment of TP53 mutation and KRAS mutation, respectively (Figure 3C). Meanwhile, Cluster C has higher tumor mutation burden and microsatellite instability (Figure 3D,E). Consistent with previous reports, the immune checkpoints expression is not correlated with TMB, which also reflects the importance of multiple indicators to predict the efficacy of immunotherapy.

### 3.3 Transcriptional regulation of epigenetic clustering

**FIGURE 4:**
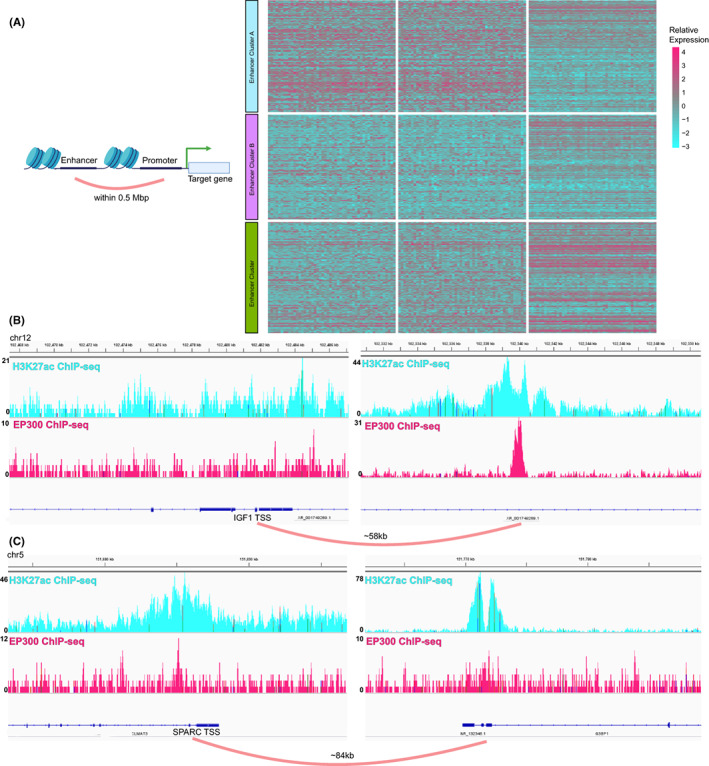
(A) The Schematic of epigenetic regulation of distant RNA by enhancers. The heatmap of differentially expressed genes among the three clusters. (B, C) The IGV screenshot of H3K27ac and EP300 ChIP‐seq in gastric tissue from ENCODE dataset.

We continue to explore the ability of epigenetic clustering to regulate transcription events. In transcription events, super‐enhancers can fold through the genome topology and regulate remote gene transcription via the core transcriptional regulatory circuitry (Figure 4A). These adjustments to distant genes mostly occur within 0.5 Mbp. We analyze the differentially expressed genes among the three clusters (Figure 4B, Tables S1–S4). We exemplify the relationship between epigenetic clustering of differentially expressed genes and differentially activated super‐enhancers (differentially expressed eRNA). About 58 kb and 83 kb downstream of the transcription start site of the mesenchymal‐related genes IGF1 and sprac, which are highly expressed in Cluster A, there are super‐enhancers specifically activated by Cluster A, respectively. (Figure 4C,D).

**FIGURE 5:**
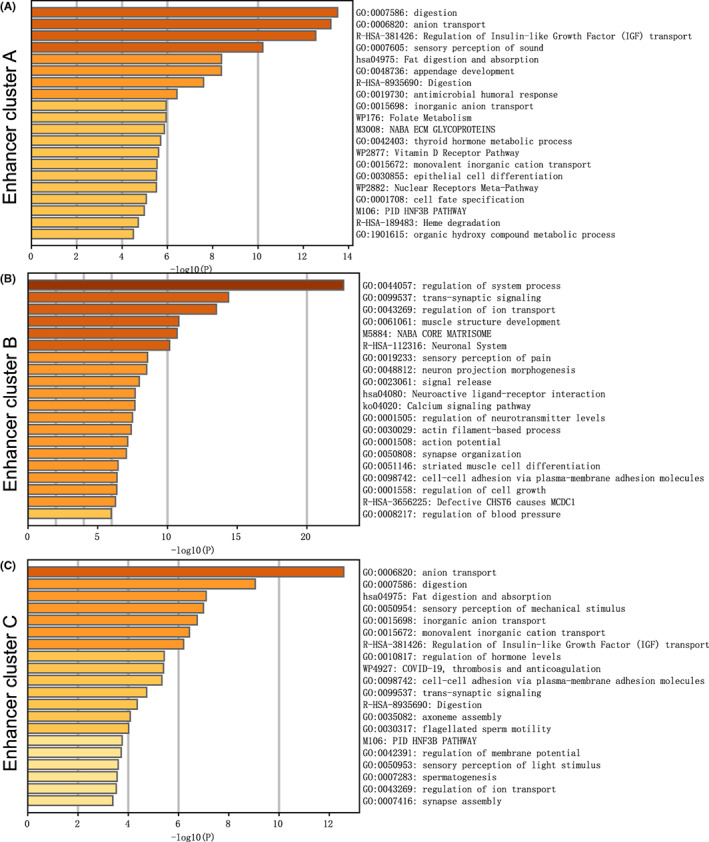
(A–C) The pathway enrichment analysis of three epigenetic clusters via Metascape.

We perform pathway enrichment for differentially expressed genes of epigenetic clustering via Metascape. Cluster A enriched in several pathways including Regulation of Insulin‐like Growth Factor (IGF) transport, epithelial‐mesenchymal cell signaling, and epithelial to mesenchymal transition (Figure 5A). Cluster B enriched in several pathway including lung epithelial cell differentiation and cell–cell adhesion via plasma‐membrane adhesion molecules (Figure 5B). Cluster B enriched in several pathways including positive regulation of FGFR (fibroblast growth factor receptor) signaling pathway and fibroblast proliferation (Figure 5C). These results verify that Clusters A and B have a worse prognosis. Cluster A has a mesenchymal cell phenotype, Cluster B has a tendency to epithelial cells, and Cluster C is characterized by a high degree of cancer‐associated fibroblasts (CAFs) infiltration which consistent with the results of previous tumor microenvironment cell abundance analysis.

### 3.4 Construction and validation of epigenetic clusters signature

**FIGURE 6:**
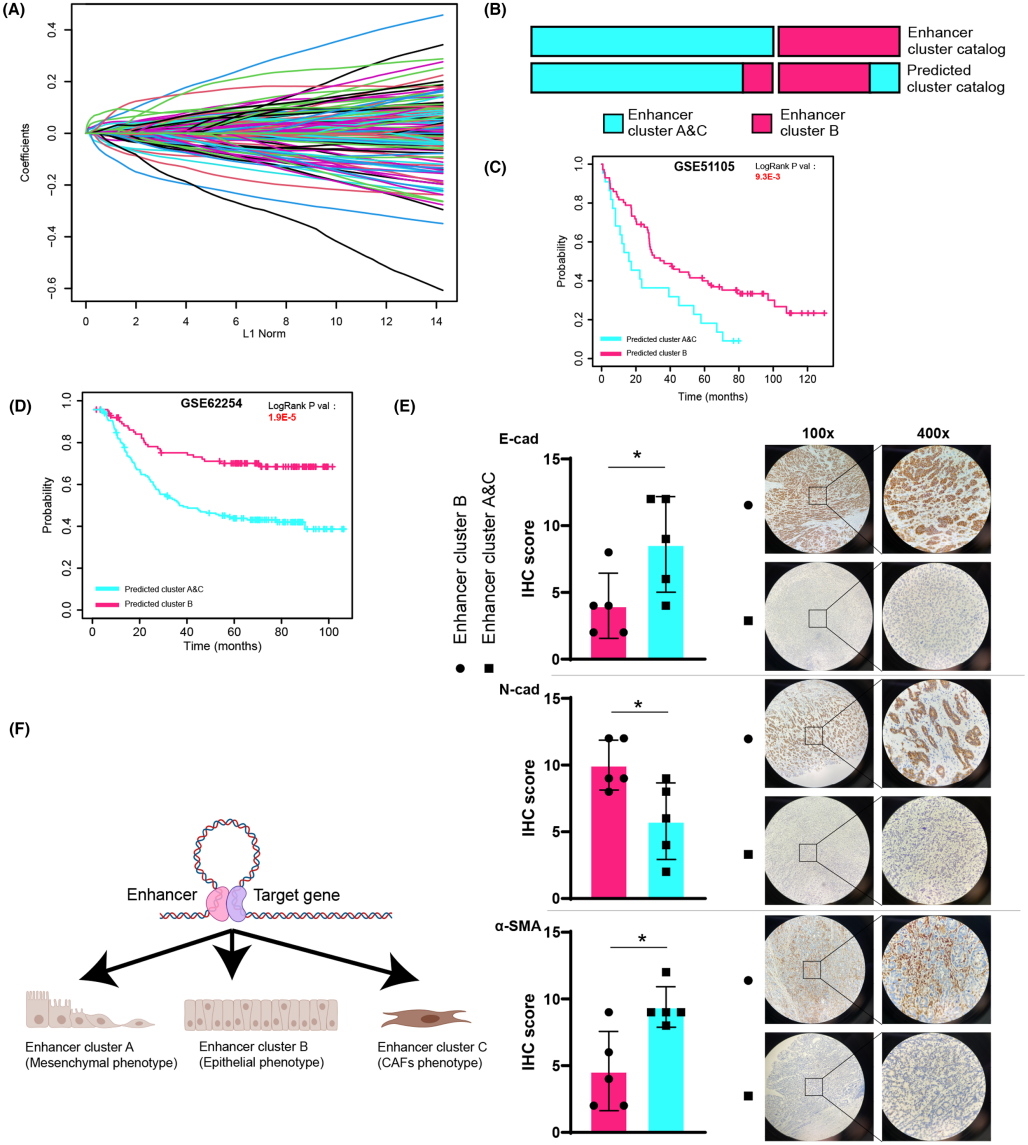
(A) The LASSO algorithm to constructed 18‐gene epigenetic clusters signature. (B) 18‐gene epigenetic clusters signature predictive ability to distinguish clusters. (C–D) The survival analysis of patients according to 18‐gene epigenetic clusters signature from GSE51105 and GSE62254. (E) Representative picture of immunohistochemistry of E‐cad, N‐cad, and α‐SMA. (F) The Schematic of STAD patients' phenotypic differences due to epigenetic heterogeneity.

To perform epigenetic clustering of patients in a small‐sample cohort, we constructed 18‐gene epigenetic clusters signature to predict epigenetic clusters via LASSO (Figure 6A, Table S4). Epigenetic clusters signature has a strong ability to predict epigenetic clusters in TCGA cohort (Figure 6B). We analyzed the survival of patients according to 18‐gene epigenetic clusters signature in two independent microarray datasets (GSE51105, GSE62254) (Figure 6C,D, Table S4). The results show that 18‐gene epigenetic clusters signature has robust predictive ability. We classified 10 patients with gastric adenocarcinoma by enhancer cluster signature via qRT‐PCR and performed immunohistochemistry on surgical specimens (Figure 6E). The result shows that cluster B patients have lower interstitial cell characteristics and CAF infiltration. In summary, we identified three unique epigenetic clusters of STAD through the differential activation of super‐enhancers, and identified a cluster with a lower EMT level and a better prognosis (Figure 6F).

## 4 DISCUSSION

Previous studies have expounded the regulation of epigenetic reprogramming on malignant tumors progression from multiple perspectives.^24^, ^25^ In this study, we performed bioinformatics analysis combined with data collected from publicly available databases for analyzing the association between eRNAs and immune microenvironment, transcriptional regulation of patients with STAD aiming at developing a grouping criteria for gastric adenocarcinoma according to eRNAs. We found a novel molecular subtype with predominantly deficiency of immune, while more CD8+ T cells and cytotoxic lymphocytes infiltration at the same time, which suggested a better response to immunotherapy. Further data also shows the difference across clusters in transcriptional regulation. In general, the findings supply a all‐embracing introduction of eRNA in gastric adenocarcinoma that presents a novel biological perspective complementary to other genomic characteristics for fully exploring the molecular mechanisms of gastric adenocarcinoma. The clinical utility of eRNA‐based molecular subtypes also offers novel insights for the gastric adenocarcinoma therapy.

Firstly, we divided patients with STAD into three subgroups according to the difference in eRNA. Interestingly, emerging results showed that the three clusters had distinct tumor microenvironments respectively. The tumor issues of cluster A exhibited less infiltration of immune cells, which is referred to as immune‐desert tumor because the microenvironment within and around tumors is the absence of massive immune cell infiltration and shows a low level of immune response rate. However, Cluster A is not less abundant in myeloid cells than Cluster B & C. This can further illustrate that myeloid cells play a “two‐sided” role, and cluster A has a stronger tendency to promote cancer progression.Cluster B has the highest immune score, and relatively more infiltration abundance of CD8+ T cells and cytotoxic lymphocytes (CTLs) tends to signify a more active immune microenvironment. Notable, the expression of multiple immune checkpoints in the tumor issue is significantly high such as PDCD1, CD274, and TIM3, which suggests Cluster B is a typical immune‐inflamed tumor. Tumor of this type often has more lymphocytes, but the normal function of CD8+ T cells and cytotoxic lymphocytes is suppressed because of the immune checkpoint, revealing less antitumor activity.^26^ Fortunately, immune checkpoint inhibitors appearing abundantly nowadays could effectively correct such kind of immune microenvironment to activate CD8+ T cells and cytotoxic lymphocytes for the improvement of survival time.^27^ Cluster C is composed of B lineage, monocytic lineage, neutrophils, and fibroblasts. Meanwhile, the expression of LAG3 and TIGIT in this group is relatively high, combined with higher tumor mutation burden (TMB) and microsatellite instability (MSI), which suggests a better response to immune checkpoint inhibitors.^28^ It deserves attention that there are insufficient lymphocytes to produce the killing effect in this tissue, which will greatly reduce the effects of immune checkpoint inhibitors.^29^ With further exploration of Methylation data analysis, we found that the CpG island methylation level of Cluster B is significantly different from the other two clusters, which may be due to the abnormal methylation of multiple sites caused by enhancer hijacking between clusters.

To gain a thorough understanding of the transcriptional level differences between these epigenetic clusters, we elaborated three genomic events. Cluster A was significantly enriched for TP53 mutations and enriched for pathways including regulation of insulin‐like growth factor (IGF) transport, epithelial‐mesenchymal cell signaling, and epithelial‐mesenchymal transition, implying that cluster A has mesenchymal cytoplasmic phenotype. Published studies suggest that TP53 plays an important role in maintaining the epithelial phenotype and inhibiting pluripotency factors to maintain a differentiated state. Following loss of p53 function, repression of pluripotency genes will therefore be lost, leading to activation of EMT. Gain‐of‐function mutant p53 further promotes EMT and stemness phenotype by activating downstream pathways.^30^ Cluster B was observed to have more KRAS mutations and was enriched for multiple pathways including lung epithelial cell differentiation and cell–cell adhesion via plasma membrane adhesion molecules. Cluster B favors epithelial cells. KRAS functions as a molecular switch, leading to overactivation of signaling networks that promote cancer growth and progression. Downstream signals of KRAS, such as MAPK and AKT, can act as mediators of mutant Ras‐induced EMT.^31^ Genovese et al. revealed that ‘low‐metastatic’ clones exhibited downregulation of KRAS signature genes, whereas ‘high‐metastatic’ clones exhibited higher expression of KRAS signature genes.^32^ In analyzing immune infiltration of the three clusters, cluster A was defined as cold tumors, while cluster B was stratified as “hot” tumors. “Hot” tumors include diseases with proinflammatory TMEs and tumor‐infiltrating lymphocytes (TILs), which tend to have higher regulatory markers, including PD‐L1, IDO, Tregs, and ultimately promote tumor immune evasion.^33^ LAG3 and TIGIT are known as “exhaustion” markers because activating T cells self‐regulates their activity and proliferation. Cluster C had higher LAG3 and TIGIT expression. Cluster C is enriched in multiple pathways, including upregulation of fibroblast growth factor receptor signaling and fibroblast proliferation. Among the genomic biomarkers, cluster C has high microsatellite instability (MSI‐H) and high tumor mutational burden (H‐TMB), indicating high neoantigen burden and tumor antigenicity. Thus, the diversity of immune states in different clusters leads to different responses to immunotherapy. The immune signature of TME has prognostic and predictive value in immunotherapy patients. Although this study provided new insights for understanding eRNAs underlying the development of gastric cancer, limitations still exist. The structure of defined eRNAs mentioned above lacks of long‐range chromosome interaction data via Hi‐C. Additionally, the limited sample size impacts the overall statistical power of the study. More efforts are needed to further validate the results.

Overall, we provided a comprehensive description of active eRNAs in gastric adenocarcinoma and proposed that the transcriptional profile of eRNAs illustrates a new biological aspect complementary to other genomic characteristic. These findings are of greatly significant because it not only supplies a better understanding of the mechanisms underlying gastric carcinogenesis, but also provides clinical implications for the therapy of gastric adenocarcinoma.

## AUTHOR CONTRIBUTIONS

Aiting Yan, Yucheng Shen and Xiaomin Lu designed the study and performed major experiments. Aiting Yan and Xiaomin Lu supported the study. Ying Chen, Aiting Yan, and Rongrong Bianare in charge of collecting the information of patients. Cuizhu Wang, Haitao Que, Xiaomin Lu, and Aiting Yan performed the bioinformatics analysis and statistics analysis. Aiting Yan and Xiaomin Lu helped in writing the draft. Rongrong Bian and Cuizhu Wang helped to revise draft and provided important suggestions.

## CONFLICT OF INTEREST

The authors declared no conflicts of interest in this work.

## ETHICS STATEMENT

The study was permitted by the Regional Ethics Committee at Affiliated Haian Hospital of Nantong University.

## Supporting information


Tables S1–S4

## Data Availability

All data is available.
