# Supplementary material for: The landscape of enhancer RNA identify prognosis‐related molecular subtypes in gastric cancer
Source: Cancer Med. 2022 Jul 8;12(2):2046–57. doi: 10.1002/cam4.4959 (PMC9883414; doi:10.1002/cam4.4959)
Supplement: Supplementary file 1 — Tables S1–S4 [file CAM4-12-2046-s001.docx]

Table S1. Difflab Cluster A

|  | ensembl | logFC | AveExpr | t | P.Value | adj.P.Val | B | gene_id |
| --- | --- | --- | --- | --- | --- | --- | --- | --- |
| ENSG00000123407 | ENSG00000123407 | -2.92292 | 4.042294 | -7.46083 | 6.16E-13 | 1.98E-10 | 18.89335 | HOXC12 |
| ENSG00000127928 | ENSG00000127928 | -2.61256 | 3.965184 | -8.32836 | 1.60E-15 | 2.11E-12 | 24.63299 | GNGT1 |
| ENSG00000225548 | ENSG00000225548 | -2.60494 | 2.794293 | -8.53736 | 3.57E-16 | 6.52E-13 | 26.08044 | LINC01980 |
| ENSG00000221867 | ENSG00000221867 | -2.6004 | 4.913651 | -5.2279 | 2.87E-07 | 5.73E-06 | 6.398369 | MAGEA3 |
| ENSG00000106536 | ENSG00000106536 | -2.58043 | 4.769036 | -8.34745 | 1.40E-15 | 1.88E-12 | 24.76418 | POU6F2 |
| ENSG00000159217 | ENSG00000159217 | -2.52979 | 6.092536 | -7.26104 | 2.27E-12 | 5.59E-10 | 17.63632 | IGF2BP1 |
| ENSG00000197172 | ENSG00000197172 | -2.49392 | 4.419369 | -5.19519 | 3.38E-07 | 6.52E-06 | 6.242549 | MAGEA6 |
| ENSG00000213401 | ENSG00000213401 | -2.34909 | 4.050271 | -5.20624 | 3.20E-07 | 6.25E-06 | 6.295107 | MAGEA12 |
| ENSG00000147246 | ENSG00000147246 | -2.31714 | 2.390443 | -8.41677 | 8.51E-16 | 1.34E-12 | 25.24232 | HTR2C |
| ENSG00000146276 | ENSG00000146276 | -2.25471 | 3.687007 | -8.09385 | 8.36E-15 | 7.06E-12 | 23.03789 | GABRR1 |
| ENSG00000147381 | ENSG00000147381 | -2.23246 | 3.211388 | -4.86783 | 1.67E-06 | 2.29E-05 | 4.729875 | MAGEA4 |
| ENSG00000011677 | ENSG00000011677 | -2.2252 | 4.263597 | -6.06499 | 3.25E-09 | 1.74E-07 | 10.66555 | GABRA3 |
| ENSG00000102313 | ENSG00000102313 | -2.21024 | 3.292464 | -10.2679 | 6.16E-22 | 1.74E-17 | 38.91344 | ITIH6 |
| ENSG00000198443 | ENSG00000198443 | -2.16713 | 2.746637 | -7.86442 | 4.08E-14 | 2.20E-11 | 21.50808 | KRTAP4-1 |
| ENSG00000185306 | ENSG00000185306 | -2.16018 | 4.319076 | -7.72572 | 1.05E-13 | 4.99E-11 | 20.59835 | C12orf56 |
| ENSG00000163295 | ENSG00000163295 | -2.15453 | 5.890989 | -6.38896 | 5.01E-10 | 4.03E-08 | 12.45613 | ALPI |
| ENSG00000229404 | ENSG00000229404 | -2.1429 | 4.749529 | -8.19542 | 4.10E-15 | 3.93E-12 | 23.72492 | LINC00858 |
| ENSG00000240893 | ENSG00000240893 | -2.14235 | 2.196708 | -11.8973 | 7.17E-28 | 4.06E-23 | 52.14185 | LINC02042 |
| ENSG00000124143 | ENSG00000124143 | -2.12784 | 4.218477 | -7.51595 | 4.28E-13 | 1.47E-10 | 19.24458 | ARHGAP40 |
| ENSG00000175329 | ENSG00000175329 | -2.11939 | 4.308223 | -5.57217 | 4.84E-08 | 1.45E-06 | 8.088979 | ISX |
| ENSG00000017427 | ENSG00000017427 | -1.12364 | 6.771074 | -5.19345 | 3.41E-07 | 1.30E-05 | 6.259985 | IGF1 |
| ENSG00000166415 | ENSG00000166415 | -2.09135 | 9.267344 | -6.78751 | 4.53E-11 | 6.22E-09 | 14.76007 | WDR72 |
| ENSG00000185686 | ENSG00000185686 | -2.06207 | 6.582748 | -5.58867 | 4.44E-08 | 1.34E-06 | 8.172264 | PRAME |
| ENSG00000187772 | ENSG00000187772 | -2.02756 | 2.046328 | -6.77362 | 4.94E-11 | 6.68E-09 | 14.67792 | LIN28B |
| ENSG00000226476 | ENSG00000226476 | -2.02752 | 4.623612 | -7.61332 | 2.23E-13 | 8.66E-11 | 19.86959 | LINC01748 |
| ENSG00000118137 | ENSG00000118137 | -1.98293 | 6.617102 | -5.44651 | 9.37E-08 | 2.43E-06 | 7.46129 | APOA1 |
| ENSG00000185247 | ENSG00000185247 | -1.97874 | 2.530595 | -5.98507 | 5.10E-09 | 2.50E-07 | 10.23548 | MAGEA11 |
| ENSG00000171759 | ENSG00000171759 | -1.97652 | 4.023675 | -6.81024 | 3.94E-11 | 5.57E-09 | 14.89472 | PAH |
| ENSG00000148942 | ENSG00000148942 | -1.95343 | 5.267314 | -8.25394 | 2.71E-15 | 3.01E-12 | 24.12342 | SLC5A12 |
| ENSG00000250546 | ENSG00000250546 | -1.94239 | 2.276653 | -8.19721 | 4.05E-15 | 3.93E-12 | 23.73707 | LOC101928978 |
| ENSG00000173157 | ENSG00000173157 | -1.928 | 2.441198 | -7.81649 | 5.66E-14 | 2.94E-11 | 21.19242 | ADAMTS20 |
| ENSG00000251165 | ENSG00000251165 | -1.92701 | 3.154547 | -7.97533 | 1.90E-14 | 1.25E-11 | 22.24378 | F11-AS1 |
| ENSG00000118271 | ENSG00000118271 | -1.92345 | 5.610033 | -5.64958 | 3.21E-08 | 1.03E-06 | 8.48162 | TTR |
| ENSG00000197444 | ENSG00000197444 | -1.91941 | 6.041314 | -5.88746 | 8.78E-09 | 3.78E-07 | 9.716592 | OGDHL |
| ENSG00000171564 | ENSG00000171564 | -1.9164 | 4.372184 | -5.09049 | 5.69E-07 | 9.86E-06 | 5.749431 | FGB |
| ENSG00000180818 | ENSG00000180818 | -1.91311 | 7.992434 | -5.30158 | 1.98E-07 | 4.31E-06 | 6.752432 | HOXC10 |
| ENSG00000124260 | ENSG00000124260 | -1.90294 | 2.341618 | -5.51351 | 6.60E-08 | 1.84E-06 | 7.794433 | MAGEA10 |
| ENSG00000088386 | ENSG00000088386 | -1.90153 | 6.537656 | -5.76942 | 1.68E-08 | 6.23E-07 | 9.098488 | SLC15A1 |
| ENSG00000084453 | ENSG00000084453 | -1.8814 | 3.825933 | -7.78988 | 6.79E-14 | 3.35E-11 | 21.01771 | SLCO1A2 |
| ENSG00000259439 | ENSG00000259439 | -1.87609 | 5.873419 | -5.8868 | 8.82E-09 | 3.78E-07 | 9.713101 | LINC01833 |
| ENSG00000123364 | ENSG00000123364 | -1.87171 | 4.652684 | -5.37276 | 1.37E-07 | 3.26E-06 | 7.098545 | HOXC13 |
| ENSG00000198930 | ENSG00000198930 | -1.87099 | 4.670181 | -4.59621 | 5.91E-06 | 6.14E-05 | 3.540447 | CSAG1 |
| ENSG00000198054 | ENSG00000198054 | -1.86553 | 2.372959 | -5.41716 | 1.09E-07 | 2.74E-06 | 7.316437 | DSCR8 |
| ENSG00000139219 | ENSG00000139219 | -1.83799 | 3.857147 | -6.03553 | 3.84E-09 | 2.00E-07 | 10.50647 | COL2A1 |
| ENSG00000140093 | ENSG00000140093 | -1.8292 | 3.718337 | -6.23221 | 1.25E-09 | 8.24E-08 | 11.58036 | SERPINA10 |
| ENSG00000188883 | ENSG00000188883 | -1.8179 | 4.026301 | -7.01338 | 1.11E-11 | 2.06E-09 | 16.1136 | KLRG2 |
| ENSG00000259664 | ENSG00000259664 | -1.79837 | 2.728395 | -7.19666 | 3.44E-12 | 7.83E-10 | 17.23669 | LINC02254 |
| ENSG00000205628 | ENSG00000205628 | -1.79818 | 2.032284 | -6.70201 | 7.66E-11 | 9.50E-09 | 14.25659 | LINC01446 |
| ENSG00000101115 | ENSG00000101115 | -1.79686 | 7.505101 | -7.93211 | 2.56E-14 | 1.54E-11 | 21.95621 | SALL4 |
| ENSG00000171560 | ENSG00000171560 | -1.79607 | 4.646239 | -4.86486 | 1.70E-06 | 2.31E-05 | 4.716514 | FGA |
| ENSG00000105852 | ENSG00000105852 | -1.77946 | 6.509923 | -6.14597 | 2.05E-09 | 1.22E-07 | 11.10607 | PON3 |
| ENSG00000196917 | ENSG00000196917 | -1.77586 | 6.494978 | -7.35124 | 1.26E-12 | 3.58E-10 | 18.20073 | HCAR1 |
| ENSG00000164199 | ENSG00000164199 | -1.77367 | 5.654821 | -8.05194 | 1.12E-14 | 8.56E-12 | 22.75616 | ADGRV1 |
| ENSG00000227674 | ENSG00000227674 | -1.75902 | 2.870545 | -5.82657 | 1.23E-08 | 4.86E-07 | 9.39642 | LINC00355 |
| ENSG00000259129 | ENSG00000259129 | -1.75426 | 2.438962 | -5.96518 | 5.70E-09 | 2.74E-07 | 10.12919 | LINC00648 |
| ENSG00000183145 | ENSG00000183145 | -1.75424 | 4.713621 | -6.56507 | 1.76E-10 | 1.82E-08 | 13.46066 | RIPPLY3 |
| ENSG00000261787 | ENSG00000261787 | -1.75325 | 3.330679 | -9.9553 | 7.52E-21 | 8.51E-17 | 36.49209 | TCF24 |
| ENSG00000110245 | ENSG00000110245 | -1.75302 | 3.383191 | -5.14735 | 4.29E-07 | 7.91E-06 | 6.016163 | APOC3 |
| ENSG00000224271 | ENSG00000224271 | -1.74905 | 3.383156 | -5.8191 | 1.28E-08 | 5.00E-07 | 9.357379 | LOC284930 |
| ENSG00000146038 | ENSG00000146038 | -1.74674 | 6.83874 | -5.91351 | 7.60E-09 | 3.39E-07 | 9.854391 | DCDC2 |
| ENSG00000081479 | ENSG00000081479 | -1.74657 | 4.302624 | -6.49807 | 2.62E-10 | 2.52E-08 | 13.07596 | LRP2 |
| ENSG00000134812 | ENSG00000134812 | -1.74175 | 5.399868 | -4.45674 | 1.10E-05 | 9.94E-05 | 2.953336 | CBLIF |
| ENSG00000189431 | ENSG00000189431 | -1.73838 | 6.90572 | -6.12585 | 2.30E-09 | 1.33E-07 | 10.99617 | RASSF10 |
| ENSG00000128714 | ENSG00000128714 | -1.73108 | 2.400946 | -5.1528 | 4.18E-07 | 7.74E-06 | 6.041874 | HOXD13 |
| ENSG00000174015 | ENSG00000174015 | -1.72856 | 2.860511 | -7.1707 | 4.07E-12 | 8.93E-10 | 17.07629 | CBY2 |
| ENSG00000276476 | ENSG00000276476 | -1.71196 | 4.258976 | -6.57103 | 1.69E-10 | 1.78E-08 | 13.49505 | LINC00540 |
| ENSG00000164411 | ENSG00000164411 | -1.70633 | 3.682977 | -6.97221 | 1.44E-11 | 2.55E-09 | 15.86435 | GJB7 |
| ENSG00000254349 | ENSG00000254349 | -1.70457 | 2.790411 | -8.0629 | 1.04E-14 | 8.15E-12 | 22.82976 | MIR2052HG |
| ENSG00000229876 | ENSG00000229876 | -1.70278 | 2.25566 | -7.52065 | 4.14E-13 | 1.43E-10 | 19.27462 | CASC20 |
| ENSG00000138083 | ENSG00000138083 | -1.69057 | 4.034702 | -6.09888 | 2.68E-09 | 1.50E-07 | 10.84933 | SIX3 |
| ENSG00000230453 | ENSG00000230453 | -1.68499 | 4.122151 | -5.84554 | 1.11E-08 | 4.48E-07 | 9.495896 | ANKRD18B |
| ENSG00000057593 | ENSG00000057593 | -1.68297 | 4.622537 | -6.46416 | 3.21E-10 | 2.94E-08 | 12.88242 | F7 |
| ENSG00000155761 | ENSG00000155761 | -1.682 | 4.840948 | -7.65692 | 1.67E-13 | 7.05E-11 | 20.15138 | SPAG17 |
| ENSG00000257842 | ENSG00000257842 | -1.68152 | 2.049474 | -6.79636 | 4.29E-11 | 5.94E-09 | 14.81245 | LINC02588 |
| ENSG00000163689 | ENSG00000163689 | -1.67512 | 6.776832 | -8.51397 | 4.23E-16 | 7.48E-13 | 25.91723 | C3orf67 |
| ENSG00000125823 | ENSG00000125823 | -1.67151 | 2.623273 | -9.06579 | 7.26E-18 | 2.74E-14 | 29.84443 | CSTL1 |
| ENSG00000263711 | ENSG00000263711 | -1.6713 | 3.422955 | -4.67063 | 4.20E-06 | 4.71E-05 | 3.860333 | LINC02864 |
| ENSG00000113722 | ENSG00000113722 | -1.6668 | 8.856063 | -4.19895 | 3.36E-05 | 0.000241 | 1.911016 | CDX1 |
| ENSG00000128713 | ENSG00000128713 | -1.66596 | 1.898068 | -6.19792 | 1.52E-09 | 9.68E-08 | 11.39117 | HOXD11 |
| ENSG00000165816 | ENSG00000165816 | -1.66172 | 8.126242 | -6.42017 | 4.16E-10 | 3.54E-08 | 12.63257 | VWA2 |
| ENSG00000185002 | ENSG00000185002 | -1.66039 | 3.973951 | -5.60165 | 4.14E-08 | 1.27E-06 | 8.237983 | RFX6 |
| ENSG00000152430 | ENSG00000152430 | -1.65861 | 3.35037 | -9.46928 | 3.37E-19 | 2.38E-15 | 32.81366 | BOLL |
| ENSG00000146039 | ENSG00000146039 | -1.65792 | 5.940163 | -4.92095 | 1.30E-06 | 1.87E-05 | 4.969471 | SLC17A4 |
| ENSG00000110195 | ENSG00000110195 | -1.64574 | 6.77011 | -4.98711 | 9.43E-07 | 1.46E-05 | 5.271114 | FOLR1 |
| ENSG00000130829 | ENSG00000130829 | -1.64491 | 4.341235 | -5.69059 | 2.57E-08 | 8.70E-07 | 8.691505 | DUSP9 |
| ENSG00000128710 | ENSG00000128710 | -1.64204 | 3.575689 | -5.69766 | 2.48E-08 | 8.49E-07 | 8.72784 | HOXD10 |
| ENSG00000250682 | ENSG00000250682 | -1.64151 | 1.742216 | -7.29764 | 1.79E-12 | 4.66E-10 | 17.86471 | LINC00491 |
| ENSG00000170289 | ENSG00000170289 | -1.63931 | 3.917688 | -8.73009 | 8.78E-17 | 2.12E-13 | 27.43628 | CNGB3 |
| ENSG00000164488 | ENSG00000164488 | -1.63786 | 5.4752 | -4.78603 | 2.46E-06 | 3.07E-05 | 4.365326 | DACT2 |
| ENSG00000064195 | ENSG00000064195 | -1.63093 | 3.74709 | -5.20852 | 3.16E-07 | 6.21E-06 | 6.305932 | DLX3 |
| ENSG00000206557 | ENSG00000206557 | -1.62888 | 3.331153 | -5.91765 | 7.43E-09 | 3.33E-07 | 9.876314 | TRIM71 |
| ENSG00000206531 | ENSG00000206531 | -1.62095 | 1.87909 | -9.77233 | 3.19E-20 | 3.01E-16 | 35.09472 | CD200R1L |
| ENSG00000182333 | ENSG00000182333 | -1.61951 | 7.558838 | -2.80189 | 0.005347 | 0.013521 | -2.73105 | LIPF |
| ENSG00000162782 | ENSG00000162782 | -1.61802 | 6.490329 | -6.18087 | 1.68E-09 | 1.05E-07 | 11.29739 | TDRD5 |
| ENSG00000053108 | ENSG00000053108 | -1.61712 | 4.592808 | -5.99265 | 4.89E-09 | 2.41E-07 | 10.27607 | FSTL4 |
| ENSG00000145864 | ENSG00000145864 | -1.6168 | 5.121887 | -6.06505 | 3.25E-09 | 1.74E-07 | 10.66587 | GABRB2 |
| ENSG00000157765 | ENSG00000157765 | -1.61138 | 7.630238 | -4.70466 | 3.59E-06 | 4.15E-05 | 4.008119 | SLC34A2 |
| ENSG00000221986 | ENSG00000221986 | -1.61115 | 3.295244 | -6.62922 | 1.19E-10 | 1.34E-08 | 13.8319 | MYBPHL |
| ENSG00000203799 | ENSG00000203799 | -1.60342 | 6.131832 | -8.06876 | 9.95E-15 | 7.93E-12 | 22.86914 | CCDC162P |
| ENSG00000228697 | ENSG00000228697 | -1.5977 | 2.470745 | -7.97937 | 1.85E-14 | 1.23E-11 | 22.2707 | LOC101928565 |
| ENSG00000267978 | ENSG00000267978 | -1.59667 | 1.772308 | -5.41277 | 1.12E-07 | 2.78E-06 | 7.294816 | MAGEA9B |
| ENSG00000249641 | ENSG00000249641 | -1.59437 | 2.200486 | -6.33331 | 6.94E-10 | 5.16E-08 | 12.14323 | HOXC13-AS |
| ENSG00000231749 | ENSG00000231749 | -1.58704 | 2.935864 | -8.01393 | 1.46E-14 | 1.06E-11 | 22.50154 | ABCA9-AS1 |
| ENSG00000163586 | ENSG00000163586 | -1.58212 | 7.733004 | -3.3952 | 0.00076 | 0.002845 | -0.97178 | FABP1 |
| ENSG00000084674 | ENSG00000084674 | -1.58113 | 6.811322 | -3.96783 | 8.71E-05 | 0.000509 | 1.024514 | APOB |
| ENSG00000046774 | ENSG00000046774 | -1.57973 | 1.857558 | -4.64134 | 4.81E-06 | 5.24E-05 | 3.733906 | MAGEC2 |
| ENSG00000228278 | ENSG00000228278 | -1.57958 | 5.079694 | -5.06248 | 6.53E-07 | 1.10E-05 | 5.618983 | ORM2 |
| ENSG00000099960 | ENSG00000099960 | -1.57581 | 6.189957 | -5.74851 | 1.88E-08 | 6.82E-07 | 8.99006 | SLC7A4 |
| ENSG00000186472 | ENSG00000186472 | -1.57488 | 7.804301 | -5.22829 | 2.86E-07 | 5.72E-06 | 6.400226 | PCLO |
| ENSG00000183844 | ENSG00000183844 | -1.57363 | 9.213855 | -5.32847 | 1.72E-07 | 3.88E-06 | 6.882735 | FAM3B |
| ENSG00000101443 | ENSG00000101443 | -1.57287 | 9.582351 | -4.72131 | 3.33E-06 | 3.90E-05 | 4.080741 | WFDC2 |
| ENSG00000185269 | ENSG00000185269 | -1.57285 | 7.382561 | -4.55809 | 7.02E-06 | 6.98E-05 | 3.378369 | NOTUM |
| ENSG00000151025 | ENSG00000151025 | -1.57192 | 6.874875 | -6.16696 | 1.82E-09 | 1.11E-07 | 11.22099 | GPR158 |
| ENSG00000231776 | ENSG00000231776 | -1.57171 | 1.60015 | -7.39729 | 9.35E-13 | 2.80E-10 | 18.49085 | LINC01611 |
| ENSG00000251151 | ENSG00000251151 | -1.56737 | 4.231832 | -5.60358 | 4.10E-08 | 1.26E-06 | 8.247734 | HOXC-AS3 |
| ENSG00000269994 | ENSG00000269994 | -1.55902 | 4.458833 | -6.39088 | 4.95E-10 | 3.99E-08 | 12.46699 | LOC440173 |
| ENSG00000106038 | ENSG00000106038 | -1.55818 | 4.286052 | -5.24761 | 2.60E-07 | 5.32E-06 | 6.492667 | EVX1 |
| ENSG00000164076 | ENSG00000164076 | -1.5559 | 3.417 | -6.62347 | 1.23E-10 | 1.37E-08 | 13.79852 | CAMKV |
| ENSG00000112818 | ENSG00000112818 | -1.55305 | 7.481017 | -4.21767 | 3.11E-05 | 0.000227 | 1.984827 | MEP1A |
| ENSG00000249574 | ENSG00000249574 | -1.55274 | 4.337367 | -7.06356 | 8.06E-12 | 1.57E-09 | 16.41894 | LOC442497 |
| ENSG00000134343 | ENSG00000134343 | -1.55093 | 4.10064 | -6.34907 | 6.33E-10 | 4.80E-08 | 12.2316 | ANO3 |
| ENSG00000136352 | ENSG00000136352 | -1.54864 | 1.77704 | -5.79956 | 1.42E-08 | 5.42E-07 | 9.255299 | NKX2-1 |
| ENSG00000091138 | ENSG00000091138 | -1.54843 | 6.098604 | -4.16036 | 3.95E-05 | 0.000274 | 1.759825 | SLC26A3 |
| ENSG00000266729 | ENSG00000266729 | -1.54715 | 2.698834 | -6.6172 | 1.28E-10 | 1.41E-08 | 13.7621 | DSG1-AS1 |
| ENSG00000101074 | ENSG00000101074 | -1.54333 | 3.676476 | -7.90567 | 3.08E-14 | 1.79E-11 | 21.78088 | R3HDML |
| ENSG00000268916 | ENSG00000268916 | -1.54327 | 3.046547 | -4.59775 | 5.87E-06 | 6.11E-05 | 3.547016 | CSAG3 |
| ENSG00000134760 | ENSG00000134760 | -1.54047 | 3.687167 | -6.01932 | 4.21E-09 | 2.15E-07 | 10.41922 | DSG1 |
| ENSG00000216588 | ENSG00000216588 | -1.53972 | 4.216063 | -6.8169 | 3.78E-11 | 5.40E-09 | 14.93423 | IGSF23 |
| ENSG00000140279 | ENSG00000140279 | -1.53915 | 10.99716 | -4.37978 | 1.55E-05 | 0.000131 | 2.636316 | DUOX2 |
| ENSG00000134258 | ENSG00000134258 | -1.53725 | 5.85516 | -5.23083 | 2.83E-07 | 5.67E-06 | 6.412392 | VTCN1 |
| ENSG00000137747 | ENSG00000137747 | -1.53429 | 6.094228 | -6.58153 | 1.59E-10 | 1.69E-08 | 13.55567 | TMPRSS13 |
| ENSG00000204983 | ENSG00000204983 | -1.52882 | 6.349469 | -4.11474 | 4.78E-05 | 0.000318 | 1.58271 | PRSS1 |
| ENSG00000164651 | ENSG00000164651 | -1.52567 | 3.806583 | -5.15173 | 4.20E-07 | 7.77E-06 | 6.036806 | SP8 |
| ENSG00000073067 | ENSG00000073067 | -1.52015 | 8.325649 | -4.71262 | 3.46E-06 | 4.03E-05 | 4.042823 | CYP2W1 |
| ENSG00000150893 | ENSG00000150893 | -1.51945 | 7.368033 | -4.91285 | 1.35E-06 | 1.93E-05 | 4.932774 | FREM2 |
| ENSG00000171557 | ENSG00000171557 | -1.5194 | 2.872539 | -4.34673 | 1.79E-05 | 0.000146 | 2.501688 | FGG |
| ENSG00000005981 | ENSG00000005981 | -1.51825 | 4.056646 | -4.43466 | 1.22E-05 | 0.000108 | 2.861874 | ASB4 |
| ENSG00000145192 | ENSG00000145192 | -1.5157 | 2.495646 | -6.064 | 3.27E-09 | 1.75E-07 | 10.66021 | AHSG |
| ENSG00000166961 | ENSG00000166961 | -1.51138 | 3.728686 | -5.94117 | 6.52E-09 | 3.01E-07 | 10.00124 | MS4A15 |
| ENSG00000215808 | ENSG00000215808 | -1.50695 | 2.807042 | -5.43681 | 9.85E-08 | 2.52E-06 | 7.41334 | LINC01139 |
| ENSG00000167434 | ENSG00000167434 | -1.50539 | 4.786064 | -5.10717 | 5.24E-07 | 9.22E-06 | 5.827413 | CA4 |
| ENSG00000180210 | ENSG00000180210 | -1.50173 | 3.699335 | -6.05262 | 3.49E-09 | 1.85E-07 | 10.59867 | F2 |
| ENSG00000070019 | ENSG00000070019 | -1.50015 | 9.648918 | -5.27297 | 2.28E-07 | 4.81E-06 | 6.614459 | GUCY2C |
| ENSG00000175928 | ENSG00000175928 | -1.49992 | 7.059428 | -5.10852 | 5.20E-07 | 9.18E-06 | 5.833731 | LRRN1 |
| ENSG00000256340 | ENSG00000256340 | -1.49912 | 6.00017 | -6.83006 | 3.48E-11 | 5.08E-09 | 15.01243 | ABCC6P1 |
| ENSG00000172782 | ENSG00000172782 | -1.4988 | 3.816085 | -5.53069 | 6.03E-08 | 1.72E-06 | 7.880434 | FADS6 |
| ENSG00000102243 | ENSG00000102243 | -1.49828 | 4.64112 | -4.33137 | 1.91E-05 | 0.000154 | 2.439446 | VGLL1 |
| ENSG00000253301 | ENSG00000253301 | -1.49642 | 2.3819 | -6.39451 | 4.85E-10 | 3.92E-08 | 12.48744 | LINC01606 |
| ENSG00000115718 | ENSG00000115718 | -1.49438 | 6.994687 | -7.16286 | 4.28E-12 | 9.28E-10 | 17.02796 | PROC |
| ENSG00000229314 | ENSG00000229314 | -1.49333 | 4.476661 | -4.10219 | 5.03E-05 | 0.000331 | 1.534301 | ORM1 |
| ENSG00000197408 | ENSG00000197408 | -1.49327 | 7.953917 | -4.53348 | 7.84E-06 | 7.60E-05 | 3.274392 | CYP2B6 |
| ENSG00000113889 | ENSG00000113889 | -1.4914 | 3.365505 | -7.08331 | 7.11E-12 | 1.41E-09 | 16.53954 | KNG1 |
| ENSG00000146530 | ENSG00000146530 | -1.49136 | 3.492167 | -5.80855 | 1.36E-08 | 5.23E-07 | 9.302231 | VWDE |
| ENSG00000250328 | ENSG00000250328 | -1.49078 | 4.632583 | -5.71327 | 2.28E-08 | 7.97E-07 | 8.808128 | MGC32805 |
| ENSG00000214039 | ENSG00000214039 | -1.48985 | 3.597488 | -4.47959 | 9.97E-06 | 9.17E-05 | 3.048416 | LINC02418 |
| ENSG00000204019 | ENSG00000204019 | -1.48875 | 5.184369 | -3.67853 | 0.000269 | 0.001246 | -0.02004 | CT83 |
| ENSG00000168412 | ENSG00000168412 | -1.48771 | 3.270773 | -6.30101 | 8.38E-10 | 6.01E-08 | 11.96259 | MTNR1A |
| ENSG00000197273 | ENSG00000197273 | -1.48695 | 3.602527 | -4.81963 | 2.10E-06 | 2.72E-05 | 4.514401 | GUCA2A |
| ENSG00000229544 | ENSG00000229544 | -1.48335 | 1.797703 | -6.70284 | 7.62E-11 | 9.48E-09 | 14.26144 | NKX1-2 |
| ENSG00000165556 | ENSG00000165556 | -1.48099 | 9.826465 | -5.0064 | 8.59E-07 | 1.36E-05 | 5.359706 | CDX2 |
| ENSG00000105707 | ENSG00000105707 | -1.48016 | 6.585113 | -4.67728 | 4.08E-06 | 4.59E-05 | 3.889148 | HPN |
| ENSG00000215146 | ENSG00000215146 | -1.47704 | 5.519498 | -6.69885 | 7.81E-11 | 9.65E-09 | 14.23806 | LOC441666 |
| ENSG00000117148 | ENSG00000117148 | -1.46882 | 4.081672 | -4.37159 | 1.60E-05 | 0.000134 | 2.60287 | ACTL8 |
| ENSG00000137875 | ENSG00000137875 | -1.46489 | 5.125952 | -6.01339 | 4.35E-09 | 2.20E-07 | 10.38733 | BCL2L10 |
| ENSG00000261863 | ENSG00000261863 | -1.46483 | 3.428913 | -5.53668 | 5.84E-08 | 1.68E-06 | 7.910447 | LINC01996 |
| ENSG00000140274 | ENSG00000140274 | -1.46284 | 7.991593 | -3.95875 | 9.03E-05 | 0.000524 | 0.99062 | DUOXA2 |
| ENSG00000198681 | ENSG00000198681 | -1.46101 | 2.593864 | -3.84445 | 0.000142 | 0.00075 | 0.570113 | MAGEA1 |
| ENSG00000125788 | ENSG00000125788 | -1.4596 | 1.226065 | -7.60997 | 2.29E-13 | 8.80E-11 | 19.84799 | DEFB126 |
| ENSG00000075461 | ENSG00000075461 | -1.45414 | 6.263415 | -4.67622 | 4.10E-06 | 4.62E-05 | 3.884514 | CACNG4 |
| ENSG00000257138 | ENSG00000257138 | -1.45165 | 2.910536 | -7.47672 | 5.54E-13 | 1.83E-10 | 18.99444 | TAS2R38 |
| ENSG00000110244 | ENSG00000110244 | -1.44823 | 4.18624 | -3.42916 | 0.000673 | 0.002585 | -0.86147 | APOA4 |
| ENSG00000196660 | ENSG00000196660 | -1.44689 | 4.418323 | -5.24668 | 2.61E-07 | 5.34E-06 | 6.488234 | SLC30A10 |
| ENSG00000184029 | ENSG00000184029 | -1.44339 | 1.784722 | -5.29507 | 2.04E-07 | 4.41E-06 | 6.720991 | DSCR4 |
| ENSG00000167780 | ENSG00000167780 | -1.44313 | 3.544924 | -5.34622 | 1.57E-07 | 3.62E-06 | 6.969055 | SOAT2 |
| ENSG00000256124 | ENSG00000256124 | -1.441 | 3.613295 | -6.48454 | 2.84E-10 | 2.67E-08 | 12.99865 | LINC01152 |
| ENSG00000130876 | ENSG00000130876 | -1.43986 | 2.939164 | -5.18056 | 3.64E-07 | 6.90E-06 | 6.173106 | SLC7A10 |
| ENSG00000105388 | ENSG00000105388 | -1.43986 | 13.97101 | -4.18026 | 3.64E-05 | 0.000256 | 1.837617 | CEACAM5 |
| ENSG00000249395 | ENSG00000249395 | -1.43794 | 6.849231 | -4.59707 | 5.88E-06 | 6.12E-05 | 3.544127 | CASC9 |
| ENSG00000101144 | ENSG00000101144 | -1.43786 | 8.644301 | -5.53038 | 6.04E-08 | 1.73E-06 | 7.878873 | BMP7 |
| ENSG00000189366 | ENSG00000189366 | -1.43598 | 6.054023 | -7.71075 | 1.16E-13 | 5.38E-11 | 20.50085 | ALG1L |
| ENSG00000128610 | ENSG00000128610 | -1.4348 | 5.976182 | -4.79766 | 2.33E-06 | 2.95E-05 | 4.416836 | FEZF1 |
| ENSG00000275896 | ENSG00000275896 | -1.43333 | 7.746662 | -3.50767 | 0.000507 | 0.002064 | -0.60252 | PRSS2 |
| ENSG00000231431 | ENSG00000231431 | -1.42728 | 3.79265 | -5.09581 | 5.54E-07 | 9.68E-06 | 5.774291 | LOC440910 |
| ENSG00000184697 | ENSG00000184697 | -1.42522 | 4.458865 | -4.0491 | 6.26E-05 | 0.000392 | 1.331024 | CLDN6 |
| ENSG00000185842 | ENSG00000185842 | -1.42473 | 7.63249 | -7.34116 | 1.35E-12 | 3.74E-10 | 18.13738 | DNAH14 |
| ENSG00000235621 | ENSG00000235621 | -1.42381 | 5.363487 | -5.54043 | 5.73E-08 | 1.66E-06 | 7.929285 | LINC00494 |
| ENSG00000096088 | ENSG00000096088 | -1.42365 | 12.07228 | -2.89162 | 0.004058 | 0.01084 | -2.48555 | PGC |
| ENSG00000174469 | ENSG00000174469 | -1.42281 | 8.812244 | -5.20614 | 3.20E-07 | 6.25E-06 | 6.294607 | CNTNAP2 |
| ENSG00000067715 | ENSG00000067715 | -1.42094 | 7.476735 | -5.71285 | 2.28E-08 | 7.98E-07 | 8.805936 | SYT1 |
| ENSG00000170927 | ENSG00000170927 | -1.41675 | 6.251268 | -5.44083 | 9.65E-08 | 2.48E-06 | 7.433211 | PKHD1 |
| ENSG00000253554 | ENSG00000253554 | -1.41573 | 1.557343 | -7.3353 | 1.40E-12 | 3.81E-10 | 18.10063 | LINC01414 |
| ENSG00000249196 | ENSG00000249196 | -1.41448 | 2.357848 | -4.47046 | 1.04E-05 | 9.46E-05 | 3.01038 | TMEM132D-AS1 |
| ENSG00000115850 | ENSG00000115850 | -1.41042 | 3.445474 | -5.85058 | 1.08E-08 | 4.39E-07 | 9.522365 | LCT |
| ENSG00000158816 | ENSG00000158816 | -1.40855 | 3.752451 | -5.2945 | 2.05E-07 | 4.42E-06 | 6.718245 | VWA5B1 |
| ENSG00000181433 | ENSG00000181433 | -1.40588 | 2.406948 | -4.59686 | 5.89E-06 | 6.12E-05 | 3.543247 | SAGE1 |
| ENSG00000229859 | ENSG00000229859 | -1.40481 | 4.424808 | -2.78043 | 0.005705 | 0.014251 | -2.78865 | PGA3 |
| ENSG00000037965 | ENSG00000037965 | -1.40447 | 6.529889 | -6.60478 | 1.38E-10 | 1.50E-08 | 13.69015 | HOXC8 |
| ENSG00000185940 | ENSG00000185940 | -1.40388 | 2.31853 | -6.383 | 5.19E-10 | 4.12E-08 | 12.42253 | KRTAP5-5 |
| ENSG00000011347 | ENSG00000011347 | -1.40212 | 9.104889 | -5.67529 | 2.79E-08 | 9.24E-07 | 8.613074 | SYT7 |
| ENSG00000251138 | ENSG00000251138 | -1.39816 | 3.034298 | -6.63539 | 1.15E-10 | 1.30E-08 | 13.86776 | LOC100507377 |
| ENSG00000188162 | ENSG00000188162 | -1.39716 | 3.016562 | -7.14214 | 4.89E-12 | 1.04E-09 | 16.90034 | OTOG |
| ENSG00000188738 | ENSG00000188738 | -1.39698 | 6.801071 | -6.77063 | 5.03E-11 | 6.74E-09 | 14.66024 | FSIP2 |
| ENSG00000224122 | ENSG00000224122 | -1.39541 | 1.085019 | -8.04383 | 1.18E-14 | 8.93E-12 | 22.70174 | POU6F2-AS1 |
| ENSG00000119121 | ENSG00000119121 | -1.38918 | 6.045056 | -6.3612 | 5.90E-10 | 4.57E-08 | 12.29977 | TRPM6 |
| ENSG00000124134 | ENSG00000124134 | -1.38883 | 4.685792 | -5.72499 | 2.14E-08 | 7.57E-07 | 8.868532 | KCNS1 |
| ENSG00000163618 | ENSG00000163618 | -1.38864 | 7.165065 | -5.14929 | 4.25E-07 | 7.84E-06 | 6.025303 | CADPS |
| ENSG00000101282 | ENSG00000101282 | -1.38852 | 4.226081 | -4.804 | 2.26E-06 | 2.89E-05 | 4.444944 | RSPO4 |
| ENSG00000226383 | ENSG00000226383 | -1.38551 | 5.019321 | -6.14745 | 2.03E-09 | 1.21E-07 | 11.11413 | LINC01876 |
| ENSG00000237361 | ENSG00000237361 | -1.38476 | 3.198563 | -4.73783 | 3.08E-06 | 3.67E-05 | 4.153059 | TUSC8 |
| ENSG00000254670 | ENSG00000254670 | -1.3845 | 3.42026 | -6.06483 | 3.26E-09 | 1.74E-07 | 10.6647 | RASSF10-DT |
| ENSG00000186009 | ENSG00000186009 | -1.3826 | 3.759259 | -3.92583 | 0.000103 | 0.000583 | 0.868352 | ATP4B |
| ENSG00000101470 | ENSG00000101470 | -1.38138 | 5.656762 | -6.45467 | 3.40E-10 | 3.07E-08 | 12.82839 | TNNC2 |
| ENSG00000123201 | ENSG00000123201 | -1.38119 | 5.394884 | -7.20587 | 3.25E-12 | 7.41E-10 | 17.29373 | GUCY1B2 |
| ENSG00000268940 | ENSG00000268940 | -1.38112 | 1.32327 | -5.09514 | 5.56E-07 | 9.70E-06 | 5.77116 | CT45A1 |
| ENSG00000170827 | ENSG00000170827 | -1.38087 | 3.97972 | -4.47105 | 1.04E-05 | 9.44E-05 | 3.012848 | CELP |
| ENSG00000116039 | ENSG00000116039 | -1.37913 | 5.501937 | -7.23466 | 2.70E-12 | 6.36E-10 | 17.47228 | ATP6V1B1 |
| ENSG00000152910 | ENSG00000152910 | -1.37835 | 2.426291 | -5.69513 | 2.51E-08 | 8.57E-07 | 8.714811 | CNTNAP4 |
| ENSG00000226887 | ENSG00000226887 | -1.37823 | 7.194835 | -5.79959 | 1.42E-08 | 5.42E-07 | 9.255462 | ERVMER34-1 |
| ENSG00000131951 | ENSG00000131951 | -1.37566 | 2.87764 | -8.2694 | 2.43E-15 | 2.81E-12 | 24.22898 | LRRC9 |
| ENSG00000166984 | ENSG00000166984 | -1.37565 | 2.758952 | -6.17001 | 1.79E-09 | 1.10E-07 | 11.23776 | TCP10L2 |
| ENSG00000264755 | ENSG00000264755 | -1.37366 | 3.338644 | -6.42439 | 4.06E-10 | 3.48E-08 | 12.65645 | MIR3131 |
| ENSG00000169550 | ENSG00000169550 | -1.37183 | 2.807086 | -4.24057 | 2.82E-05 | 0.000211 | 2.075512 | MUC15 |
| ENSG00000196748 | ENSG00000196748 | -1.37135 | 1.86688 | -7.24639 | 2.50E-12 | 5.97E-10 | 17.54519 | CLPSL2 |
| ENSG00000100473 | ENSG00000100473 | -1.36959 | 7.591186 | -6.28139 | 9.40E-10 | 6.56E-08 | 11.85326 | COCH |
| ENSG00000171804 | ENSG00000171804 | -1.36944 | 3.009247 | -6.91313 | 2.08E-11 | 3.44E-09 | 15.50868 | WDR87 |
| ENSG00000090402 | ENSG00000090402 | -1.36771 | 7.858456 | -3.1436 | 0.001803 | 0.005651 | -1.75682 | SI |
| ENSG00000113100 | ENSG00000113100 | -1.36706 | 1.153037 | -6.76243 | 5.29E-11 | 7.03E-09 | 14.61182 | CDH9 |
| ENSG00000120440 | ENSG00000120440 | -1.36535 | 4.419268 | -6.43769 | 3.75E-10 | 3.28E-08 | 12.73189 | TTLL2 |
| ENSG00000153303 | ENSG00000153303 | -1.36351 | 4.624875 | -5.25604 | 2.49E-07 | 5.15E-06 | 6.533113 | FRMD1 |
| ENSG00000204740 | ENSG00000204740 | -1.36191 | 5.873497 | -3.92958 | 0.000101 | 0.000575 | 0.882248 | MALRD1 |
| ENSG00000204140 | ENSG00000204140 | -1.35898 | 2.464993 | -5.91972 | 7.35E-09 | 3.30E-07 | 9.887297 | CLPSL1 |
| ENSG00000225329 | ENSG00000225329 | -1.35523 | 6.757425 | -4.97812 | 9.85E-07 | 1.51E-05 | 5.229915 | LHFPL3-AS2 |
| ENSG00000234695 | ENSG00000234695 | -1.35499 | 1.575494 | -7.71478 | 1.13E-13 | 5.28E-11 | 20.52708 | LOC105375401 |
| ENSG00000163530 | ENSG00000163530 | -1.35325 | 1.524558 | -6.51625 | 2.35E-10 | 2.30E-08 | 13.18 | DPPA2 |
| ENSG00000126890 | ENSG00000126890 | -1.34817 | 2.142473 | -3.55215 | 0.000431 | 0.001812 | -0.45337 | CTAG2 |
| ENSG00000172461 | ENSG00000172461 | -1.34541 | 6.295849 | -3.54581 | 0.000441 | 0.001847 | -0.47472 | FUT9 |
| ENSG00000146166 | ENSG00000146166 | -1.3453 | 3.702345 | -4.20571 | 3.27E-05 | 0.000236 | 1.937628 | LGSN |
| ENSG00000165215 | ENSG00000165215 | -1.34172 | 12.04213 | -4.53635 | 7.74E-06 | 7.53E-05 | 3.286491 | CLDN3 |
| ENSG00000184368 | ENSG00000184368 | -1.33979 | 5.849467 | -4.73034 | 3.19E-06 | 3.78E-05 | 4.120274 | MAP7D2 |
| ENSG00000138207 | ENSG00000138207 | -1.33968 | 8.323727 | -4.44911 | 1.14E-05 | 0.000102 | 2.921665 | RBP4 |
| ENSG00000178828 | ENSG00000178828 | -1.33835 | 7.462011 | -4.7657 | 2.71E-06 | 3.32E-05 | 4.275555 | RNF186 |
| ENSG00000101098 | ENSG00000101098 | -1.33832 | 3.821683 | -4.57554 | 6.49E-06 | 6.59E-05 | 3.45244 | RIMS4 |
| ENSG00000047936 | ENSG00000047936 | -1.3321 | 4.65671 | -5.05765 | 6.69E-07 | 1.12E-05 | 5.596565 | ROS1 |
| ENSG00000133454 | ENSG00000133454 | -1.33155 | 4.625363 | -4.96354 | 1.06E-06 | 1.60E-05 | 5.163252 | MYO18B |
| ENSG00000229005 | ENSG00000229005 | -1.33098 | 4.844888 | -7.98138 | 1.83E-14 | 1.23E-11 | 22.28412 | HNF4A-AS1 |
| ENSG00000005421 | ENSG00000005421 | -1.33003 | 3.763354 | -5.28738 | 2.12E-07 | 4.53E-06 | 6.683887 | PON1 |
| ENSG00000164794 | ENSG00000164794 | -1.32712 | 2.088631 | -5.63246 | 3.51E-08 | 1.11E-06 | 8.394419 | KCNV1 |
| ENSG00000143512 | ENSG00000143512 | -1.32612 | 3.446991 | -7.47525 | 5.60E-13 | 1.83E-10 | 18.98509 | HHIPL2 |
| ENSG00000155495 | ENSG00000155495 | -1.32461 | 1.830472 | -4.20308 | 3.30E-05 | 0.000238 | 1.927247 | MAGEC1 |
| ENSG00000187658 | ENSG00000187658 | -1.32364 | 2.755973 | -6.38856 | 5.02E-10 | 4.03E-08 | 12.45388 | C5orf52 |
| ENSG00000115112 | ENSG00000115112 | -1.32362 | 10.12653 | -6.26645 | 1.02E-09 | 7.01E-08 | 11.77021 | TFCP2L1 |
| ENSG00000246876 | ENSG00000246876 | -1.32291 | 1.152955 | -7.47818 | 5.49E-13 | 1.83E-10 | 19.0037 | LINC02466 |
| ENSG00000021488 | ENSG00000021488 | -1.32264 | 6.323103 | -4.77391 | 2.60E-06 | 3.22E-05 | 4.311772 | SLC7A9 |
| ENSG00000117834 | ENSG00000117834 | -1.32247 | 6.519788 | -5.99939 | 4.71E-09 | 2.34E-07 | 10.3122 | SLC5A9 |
| ENSG00000255545 | ENSG00000255545 | -1.32126 | 5.30602 | -4.63379 | 4.98E-06 | 5.38E-05 | 3.701399 | LOC283177 |
| ENSG00000132677 | ENSG00000132677 | -1.32052 | 3.869025 | -5.80409 | 1.39E-08 | 5.32E-07 | 9.278947 | RHBG |
| ENSG00000108244 | ENSG00000108244 | -1.31917 | 7.745932 | -3.83993 | 0.000145 | 0.000761 | 0.553721 | KRT23 |
| ENSG00000225546 | ENSG00000225546 | -1.31839 | 1.410691 | -6.09887 | 2.68E-09 | 1.50E-07 | 10.84927 | LINC02476 |
| ENSG00000177359 | ENSG00000177359 | -1.31808 | 5.286598 | -6.78151 | 4.70E-11 | 6.38E-09 | 14.72457 | OVOS2 |
| ENSG00000188338 | ENSG00000188338 | -1.31589 | 5.004516 | -4.98971 | 9.31E-07 | 1.44E-05 | 5.283036 | SLC38A3 |
| ENSG00000254233 | ENSG00000254233 | -1.31152 | 3.543401 | -5.52055 | 6.36E-08 | 1.79E-06 | 7.82967 | LINC02365 |
| ENSG00000006377 | ENSG00000006377 | -1.31043 | 4.535285 | -4.15298 | 4.08E-05 | 0.00028 | 1.731046 | DLX6 |
| ENSG00000123388 | ENSG00000123388 | -1.30909 | 7.764836 | -5.0372 | 7.39E-07 | 1.21E-05 | 5.50182 | HOXC11 |
| ENSG00000186094 | ENSG00000186094 | -1.30844 | 3.427837 | -6.39897 | 4.72E-10 | 3.88E-08 | 12.51266 | AGBL4 |
| ENSG00000248973 | ENSG00000248973 | -1.30752 | 1.034763 | -7.08335 | 7.11E-12 | 1.41E-09 | 16.5398 | LOC107986400 |
| ENSG00000178445 | ENSG00000178445 | -1.30649 | 6.47398 | -5.28737 | 2.12E-07 | 4.53E-06 | 6.683854 | GLDC |
| ENSG00000157423 | ENSG00000157423 | -1.30431 | 4.172799 | -6.43715 | 3.77E-10 | 3.28E-08 | 12.72883 | HYDIN |
| ENSG00000187416 | ENSG00000187416 | -1.30391 | 4.69065 | -4.81567 | 2.14E-06 | 2.76E-05 | 4.4968 | LHFPL3 |
| ENSG00000236155 | ENSG00000236155 | -1.30186 | 4.33589 | -6.48555 | 2.83E-10 | 2.67E-08 | 13.00442 | ZPLD2P |
| ENSG00000205517 | ENSG00000205517 | -1.30098 | 8.001074 | -6.01528 | 4.31E-09 | 2.19E-07 | 10.39747 | RGL3 |
| ENSG00000181333 | ENSG00000181333 | -1.30044 | 4.829923 | -6.38927 | 5.00E-10 | 4.02E-08 | 12.45787 | HEPHL1 |
| ENSG00000272549 | ENSG00000272549 | -1.29896 | 3.429203 | -6.17675 | 1.72E-09 | 1.07E-07 | 11.27472 | LINC02538 |
| ENSG00000245750 | ENSG00000245750 | -1.29863 | 4.523724 | -5.96189 | 5.81E-09 | 2.78E-07 | 10.1116 | DRAIC |
| ENSG00000162949 | ENSG00000162949 | -1.29843 | 7.524811 | -4.67956 | 4.04E-06 | 4.56E-05 | 3.899024 | CAPN13 |
| ENSG00000110243 | ENSG00000110243 | -1.29819 | 2.254542 | -5.86516 | 9.93E-09 | 4.13E-07 | 9.598985 | APOA5 |
| ENSG00000162344 | ENSG00000162344 | -1.2972 | 3.849513 | -4.09231 | 5.24E-05 | 0.000341 | 1.496299 | FGF19 |
| ENSG00000110148 | ENSG00000110148 | -1.294 | 3.966273 | -4.43714 | 1.20E-05 | 0.000107 | 2.872136 | CCKBR |
| ENSG00000171126 | ENSG00000171126 | -1.29397 | 4.030934 | -6.08887 | 2.84E-09 | 1.57E-07 | 10.79495 | KCNG3 |
| ENSG00000241224 | ENSG00000241224 | -1.2937 | 3.63886 | -4.3859 | 1.51E-05 | 0.000128 | 2.661353 | C3orf85 |
| ENSG00000169548 | ENSG00000169548 | -1.29351 | 2.478354 | -5.33049 | 1.70E-07 | 3.85E-06 | 6.892528 | ZNF280A |
| ENSG00000269586 | ENSG00000269586 | -1.29295 | 1.179778 | -5.16 | 4.03E-07 | 7.51E-06 | 6.075841 | CT45A10 |
| ENSG00000249201 | ENSG00000249201 | -1.29271 | 3.620011 | -4.87579 | 1.61E-06 | 2.22E-05 | 4.765637 | CTD-3080P12.3 |
| ENSG00000164007 | ENSG00000164007 | -1.288 | 3.250543 | -5.86687 | 9.84E-09 | 4.10E-07 | 9.608017 | CLDN19 |
| ENSG00000120054 | ENSG00000120054 | -1.28712 | 2.240494 | -4.86085 | 1.73E-06 | 2.34E-05 | 4.698539 | CPN1 |
| ENSG00000079112 | ENSG00000079112 | -1.28699 | 11.93826 | -3.78778 | 0.000177 | 0.000892 | 0.365843 | CDH17 |
| ENSG00000232023 | ENSG00000232023 | -1.28301 | 1.443446 | -5.79582 | 1.45E-08 | 5.51E-07 | 9.235794 | LINC01807 |
| ENSG00000232040 | ENSG00000232040 | -1.28046 | 3.99509 | -4.67319 | 4.16E-06 | 4.67E-05 | 3.871391 | ZBED9 |
| ENSG00000230316 | ENSG00000230316 | -1.2796 | 7.101241 | -4.25985 | 2.60E-05 | 0.000198 | 2.15219 | FEZF1-AS1 |
| ENSG00000152591 | ENSG00000152591 | -1.27911 | 1.638691 | -7.44787 | 6.71E-13 | 2.10E-10 | 18.81107 | DSPP |
| ENSG00000187889 | ENSG00000187889 | -1.27891 | 3.138269 | -5.59813 | 4.22E-08 | 1.29E-06 | 8.220124 | FYB2 |
| ENSG00000274993 | ENSG00000274993 | -1.27819 | 6.10369 | -5.00682 | 8.57E-07 | 1.35E-05 | 5.361668 | LOC105375431 |
| ENSG00000079689 | ENSG00000079689 | -1.27627 | 4.061372 | -4.01035 | 7.33E-05 | 0.000444 | 1.184181 | SCGN |
| ENSG00000137968 | ENSG00000137968 | -1.27395 | 7.355985 | -4.53375 | 7.83E-06 | 7.59E-05 | 3.275535 | SLC44A5 |
| ENSG00000143556 | ENSG00000143556 | -1.27251 | 4.149777 | -2.99923 | 0.002889 | 0.008238 | -2.18141 | S100A7 |
| ENSG00000112333 | ENSG00000112333 | -1.27201 | 2.344936 | -6.09889 | 2.68E-09 | 1.50E-07 | 10.84935 | NR2E1 |
| ENSG00000160224 | ENSG00000160224 | -1.27089 | 3.338133 | -6.41408 | 4.32E-10 | 3.64E-08 | 12.5981 | AIRE |
| ENSG00000107984 | ENSG00000107984 | -1.26994 | 7.367135 | -3.30263 | 0.001051 | 0.003684 | -1.26721 | DKK1 |
| ENSG00000172238 | ENSG00000172238 | -1.26698 | 5.108631 | -3.66662 | 0.000282 | 0.001291 | -0.06148 | ATOH1 |
| ENSG00000165863 | ENSG00000165863 | -1.26693 | 3.184468 | -4.90913 | 1.37E-06 | 1.95E-05 | 4.915968 | C10orf82 |
| ENSG00000081051 | ENSG00000081051 | -1.2668 | 2.746939 | -4.33507 | 1.88E-05 | 0.000152 | 2.45441 | AFP |
| ENSG00000253641 | ENSG00000253641 | -1.26485 | 4.92057 | -6.27004 | 1.00E-09 | 6.89E-08 | 11.79015 | LINCR-0001 |
| ENSG00000175985 | ENSG00000175985 | -1.26388 | 4.756397 | -6.65974 | 9.91E-11 | 1.17E-08 | 14.00955 | PLEKHD1 |
| ENSG00000124939 | ENSG00000124939 | -1.26347 | 3.834484 | -4.28181 | 2.36E-05 | 0.000183 | 2.239938 | SCGB2A1 |
| ENSG00000273777 | ENSG00000273777 | -1.26267 | 3.402821 | -4.92214 | 1.29E-06 | 1.86E-05 | 4.974875 | CEACAM20 |
| ENSG00000256612 | ENSG00000256612 | -1.26221 | 6.358304 | -4.98736 | 9.42E-07 | 1.46E-05 | 5.27227 | CYP2B7P |
| ENSG00000206129 | ENSG00000206129 | -1.26211 | 2.458674 | -6.06564 | 3.24E-09 | 1.74E-07 | 10.66905 | LOC642484 |
| ENSG00000277363 | ENSG00000277363 | -1.26157 | 9.070279 | -6.68225 | 8.64E-11 | 1.04E-08 | 14.14096 | SRCIN1 |
| ENSG00000168907 | ENSG00000168907 | -1.25938 | 8.670043 | -5.29111 | 2.08E-07 | 4.47E-06 | 6.701862 | PLA2G4F |
| ENSG00000138308 | ENSG00000138308 | -1.25912 | 3.613153 | -4.16643 | 3.85E-05 | 0.000268 | 1.783512 | PLA2G12B |
| ENSG00000167759 | ENSG00000167759 | -1.25868 | 5.40485 | -3.95665 | 9.11E-05 | 0.000528 | 0.982796 | KLK13 |
| ENSG00000177459 | ENSG00000177459 | -1.25536 | 6.598689 | -4.80282 | 2.27E-06 | 2.90E-05 | 4.439716 | ERICH5 |
| ENSG00000232164 | ENSG00000232164 | -1.25467 | 2.108815 | -8.20811 | 3.75E-15 | 3.93E-12 | 23.81115 | LINC01873 |
| ENSG00000148702 | ENSG00000148702 | -1.25365 | 8.991984 | -3.72143 | 0.000229 | 0.001092 | 0.130216 | HABP2 |
| ENSG00000269526 | ENSG00000269526 | -1.25222 | 1.837998 | -6.88567 | 2.47E-11 | 3.94E-09 | 15.3441 | ERVV-1 |
| ENSG00000152977 | ENSG00000152977 | -1.25182 | 2.434582 | -4.48724 | 9.64E-06 | 8.93E-05 | 3.080357 | ZIC1 |
| ENSG00000142449 | ENSG00000142449 | -1.25141 | 4.069434 | -5.05195 | 6.88E-07 | 1.14E-05 | 5.570123 | FBN3 |
| ENSG00000259803 | ENSG00000259803 | -1.25112 | 4.524077 | -4.43199 | 1.23E-05 | 0.000109 | 2.850851 | SLC22A31 |
| ENSG00000180537 | ENSG00000180537 | -1.25007 | 4.084274 | -5.12499 | 4.80E-07 | 8.64E-06 | 5.910955 | RNF182 |
| ENSG00000177202 | ENSG00000177202 | -1.24895 | 6.259522 | -7.82113 | 5.49E-14 | 2.88E-11 | 21.2229 | SPACA4 |
| ENSG00000185038 | ENSG00000185038 | -1.24667 | 3.035871 | -5.36411 | 1.43E-07 | 3.36E-06 | 7.056273 | MROH2A |
| ENSG00000197353 | ENSG00000197353 | -1.24595 | 5.108846 | -3.22804 | 0.001357 | 0.004516 | -1.49968 | LYPD2 |
| ENSG00000145506 | ENSG00000145506 | -1.24487 | 7.945494 | -5.2724 | 2.29E-07 | 4.82E-06 | 6.611726 | NKD2 |
| ENSG00000182040 | ENSG00000182040 | -1.24443 | 2.694106 | -5.3272 | 1.73E-07 | 3.89E-06 | 6.876578 | USH1G |
| ENSG00000224057 | ENSG00000224057 | -1.24404 | 4.512297 | -5.57612 | 4.74E-08 | 1.42E-06 | 8.108879 | EGFR-AS1 |
| ENSG00000233392 | ENSG00000233392 | -1.24213 | 3.284749 | -4.86 | 1.74E-06 | 2.35E-05 | 4.694742 | LOC200772 |
| ENSG00000080618 | ENSG00000080618 | -1.24153 | 2.554158 | -6.80455 | 4.08E-11 | 5.72E-09 | 14.86096 | CPB2 |
| ENSG00000176381 | ENSG00000176381 | -1.24153 | 3.011715 | -5.18163 | 3.62E-07 | 6.88E-06 | 6.178205 | PRR18 |
| ENSG00000139352 | ENSG00000139352 | -1.24004 | 3.4202 | -4.71644 | 3.40E-06 | 3.97E-05 | 4.059504 | ASCL1 |
| ENSG00000187017 | ENSG00000187017 | -1.239 | 9.833029 | -5.80322 | 1.40E-08 | 5.34E-07 | 9.27439 | ESPN |
| ENSG00000154997 | ENSG00000154997 | -1.23823 | 2.09677 | -5.72662 | 2.12E-08 | 7.53E-07 | 8.876919 | SEPTIN14 |
| ENSG00000180347 | ENSG00000180347 | -1.23734 | 4.35988 | -5.08227 | 5.92E-07 | 1.02E-05 | 5.711114 | ITPRID1 |
| ENSG00000158516 | ENSG00000158516 | -1.23557 | 4.162775 | -3.72926 | 0.000222 | 0.001066 | 0.157833 | CPA2 |
| ENSG00000198610 | ENSG00000198610 | -1.23526 | 5.581632 | -5.09302 | 5.62E-07 | 9.77E-06 | 5.761238 | AKR1C4 |
| ENSG00000156076 | ENSG00000156076 | -1.23411 | 2.871001 | -4.32805 | 1.94E-05 | 0.000156 | 2.426008 | WIF1 |
| ENSG00000179455 | ENSG00000179455 | -1.23304 | 4.000952 | -4.33178 | 1.91E-05 | 0.000154 | 2.441091 | MKRN3 |
| ENSG00000157542 | ENSG00000157542 | -1.23236 | 5.089077 | -5.8407 | 1.14E-08 | 4.57E-07 | 9.470473 | KCNJ6 |
| ENSG00000113494 | ENSG00000113494 | -1.23195 | 9.111159 | -5.06947 | 6.31E-07 | 1.07E-05 | 5.651499 | PRLR |
| ENSG00000101104 | ENSG00000101104 | -1.23104 | 10.04116 | -8.41061 | 8.90E-16 | 1.36E-12 | 25.19971 | PABPC1L |
| ENSG00000179914 | ENSG00000179914 | -1.23045 | 7.287679 | -3.17926 | 0.001601 | 0.005137 | -1.64901 | ITLN1 |
| ENSG00000152592 | ENSG00000152592 | -1.23011 | 2.727135 | -6.16597 | 1.83E-09 | 1.11E-07 | 11.21559 | DMP1 |
| ENSG00000149742 | ENSG00000149742 | -1.22775 | 2.600539 | -6.34388 | 6.53E-10 | 4.92E-08 | 12.20248 | SLC22A9 |
| ENSG00000257185 | ENSG00000257185 | -1.22601 | 1.156448 | -6.75749 | 5.45E-11 | 7.21E-09 | 14.58272 | LINC02293 |
| ENSG00000163515 | ENSG00000163515 | -1.22572 | 3.412988 | -4.6264 | 5.15E-06 | 5.52E-05 | 3.669671 | RETNLB |
| ENSG00000223760 | ENSG00000223760 | -1.22485 | 1.57936 | -5.76056 | 1.76E-08 | 6.49E-07 | 9.052532 | MED15P9 |
| ENSG00000226416 | ENSG00000226416 | -1.22404 | 1.789221 | -5.74512 | 1.92E-08 | 6.92E-07 | 8.972515 | MRPL23-AS1 |
| ENSG00000229243 | ENSG00000229243 | -1.22295 | 1.459312 | -7.63873 | 1.88E-13 | 7.73E-11 | 20.03369 | LINC01981 |
| ENSG00000203650 | ENSG00000203650 | -1.22179 | 3.41449 | -7.17073 | 4.07E-12 | 8.93E-10 | 17.07646 | LINC01285 |
| ENSG00000149972 | ENSG00000149972 | -1.22159 | 2.876245 | -5.79228 | 1.48E-08 | 5.60E-07 | 9.217387 | CNTN5 |
| ENSG00000251381 | ENSG00000251381 | -1.2196 | 4.06243 | -4.42726 | 1.26E-05 | 0.00011 | 2.83131 | LINC00958 |
| ENSG00000158874 | ENSG00000158874 | -1.21896 | 3.586262 | -3.77029 | 0.00019 | 0.00094 | 0.303366 | APOA2 |
| ENSG00000166840 | ENSG00000166840 | -1.21883 | 3.800128 | -5.66969 | 2.88E-08 | 9.46E-07 | 8.584415 | GLYATL1 |
| ENSG00000136327 | ENSG00000136327 | -1.21727 | 2.13023 | -4.55598 | 7.08E-06 | 7.02E-05 | 3.369442 | NKX2-8 |
| ENSG00000205359 | ENSG00000205359 | -1.21651 | 1.150808 | -7.05193 | 8.68E-12 | 1.67E-09 | 16.34799 | SLCO6A1 |
| ENSG00000272808 | ENSG00000272808 | -1.21602 | 3.484567 | -6.28204 | 9.36E-10 | 6.54E-08 | 11.85691 | LOC105369201 |
| ENSG00000154080 | ENSG00000154080 | -1.21575 | 3.93432 | -3.96746 | 8.72E-05 | 0.000509 | 1.023146 | CHST9 |
| ENSG00000165078 | ENSG00000165078 | -1.21445 | 4.078214 | -4.91866 | 1.31E-06 | 1.89E-05 | 4.959101 | CPA6 |
| ENSG00000136944 | ENSG00000136944 | -1.21189 | 5.307309 | -4.2567 | 2.63E-05 | 0.0002 | 2.139633 | LMX1B |
| ENSG00000231646 | ENSG00000231646 | -1.21151 | 2.7276 | -7.04895 | 8.84E-12 | 1.70E-09 | 16.32985 | FSIP2-AS1 |
| ENSG00000140015 | ENSG00000140015 | -1.2098 | 2.557775 | -5.05473 | 6.78E-07 | 1.13E-05 | 5.583038 | KCNH5 |
| ENSG00000171121 | ENSG00000171121 | -1.20536 | 6.515772 | -8.36653 | 1.22E-15 | 1.72E-12 | 24.89549 | KCNMB3 |
| ENSG00000124232 | ENSG00000124232 | -1.20415 | 2.950131 | -6.02979 | 3.97E-09 | 2.05E-07 | 10.47555 | RBPJL |
| ENSG00000155052 | ENSG00000155052 | -1.20322 | 2.421375 | -5.33336 | 1.68E-07 | 3.80E-06 | 6.906467 | CNTNAP5 |
| ENSG00000183760 | ENSG00000183760 | -1.20179 | 3.008896 | -5.39633 | 1.22E-07 | 2.96E-06 | 7.214019 | ACP7 |
| ENSG00000182111 | ENSG00000182111 | -1.20163 | 2.227078 | -4.90052 | 1.43E-06 | 2.02E-05 | 4.877051 | ZNF716 |
| ENSG00000166763 | ENSG00000166763 | -1.19961 | 4.153248 | -7.62709 | 2.04E-13 | 8.12E-11 | 19.95847 | STRCP1 |
| ENSG00000215148 | ENSG00000215148 | -1.19868 | 3.035884 | -4.70766 | 3.54E-06 | 4.11E-05 | 4.021164 | PRSS41 |
| ENSG00000234928 | ENSG00000234928 | -1.19718 | 2.416924 | -5.19764 | 3.34E-07 | 6.46E-06 | 6.254195 | LINC01659 |
| ENSG00000100593 | ENSG00000100593 | -1.19713 | 3.168565 | -4.84348 | 1.88E-06 | 2.49E-05 | 4.620786 | ISM2 |
| ENSG00000180999 | ENSG00000180999 | -1.19648 | 2.352618 | -7.09201 | 6.73E-12 | 1.36E-09 | 16.59279 | C1orf105 |
| ENSG00000105641 | ENSG00000105641 | -1.19597 | 6.169369 | -3.80289 | 0.000167 | 0.000852 | 0.420015 | SLC5A5 |
| ENSG00000173557 | ENSG00000173557 | -1.19585 | 7.080236 | -5.11284 | 5.09E-07 | 9.03E-06 | 5.853961 | FAM166C |
| ENSG00000112796 | ENSG00000112796 | -1.19578 | 7.591582 | -5.45422 | 9.00E-08 | 2.36E-06 | 7.499453 | ENPP5 |
| ENSG00000124159 | ENSG00000124159 | -1.19564 | 4.100642 | -7.74134 | 9.44E-14 | 4.53E-11 | 20.7002 | MATN4 |
| ENSG00000123999 | ENSG00000123999 | -1.19428 | 3.827844 | -4.48068 | 9.92E-06 | 9.14E-05 | 3.05296 | INHA |
| ENSG00000147255 | ENSG00000147255 | -1.19391 | 5.582148 | -4.84567 | 1.86E-06 | 2.47E-05 | 4.630548 | IGSF1 |
| ENSG00000242866 | ENSG00000242866 | -1.19386 | 3.742171 | -6.9348 | 1.82E-11 | 3.06E-09 | 15.63888 | STRC |
| ENSG00000141744 | ENSG00000141744 | -1.1938 | 3.895015 | -5.11293 | 5.09E-07 | 9.02E-06 | 5.854393 | PNMT |
| ENSG00000183960 | ENSG00000183960 | -1.19191 | 6.742912 | -4.87189 | 1.64E-06 | 2.25E-05 | 4.748074 | KCNH8 |
| ENSG00000134538 | ENSG00000134538 | -1.19158 | 2.232103 | -5.10109 | 5.40E-07 | 9.46E-06 | 5.798981 | SLCO1B1 |
| ENSG00000233217 | ENSG00000233217 | -1.19005 | 5.425628 | -5.7315 | 2.06E-08 | 7.35E-07 | 8.902118 | MROH3P |
| ENSG00000163501 | ENSG00000163501 | -1.18994 | 9.800104 | -4.47319 | 1.03E-05 | 9.38E-05 | 3.021736 | IHH |
| ENSG00000214049 | ENSG00000214049 | -1.1899 | 8.086717 | -3.82364 | 0.000154 | 0.000799 | 0.494774 | UCA1 |
| ENSG00000268089 | ENSG00000268089 | -1.18832 | 3.727044 | -5.52254 | 6.29E-08 | 1.78E-06 | 7.839618 | GABRQ |
| ENSG00000168955 | ENSG00000168955 | -1.18829 | 8.316676 | -3.00887 | 0.002801 | 0.008036 | -2.15365 | TM4SF20 |
| ENSG00000183463 | ENSG00000183463 | -1.18807 | 1.664755 | -5.0638 | 6.49E-07 | 1.09E-05 | 5.625124 | URAD |
| ENSG00000169347 | ENSG00000169347 | -1.18777 | 8.643516 | -2.90505 | 0.003892 | 0.010484 | -2.44819 | GP2 |
| ENSG00000140254 | ENSG00000140254 | -1.18721 | 6.428919 | -4.95435 | 1.10E-06 | 1.65E-05 | 5.121301 | DUOXA1 |
| ENSG00000130054 | ENSG00000130054 | -1.18707 | 5.560549 | -3.84878 | 0.00014 | 0.000741 | 0.585822 | FAM155B |
| ENSG00000236039 | ENSG00000236039 | -1.18675 | 4.723743 | -4.95888 | 1.08E-06 | 1.62E-05 | 5.141978 | LOC101927630 |
| ENSG00000143921 | ENSG00000143921 | -1.18583 | 4.912143 | -4.15184 | 4.10E-05 | 0.000281 | 1.726586 | ABCG8 |
| ENSG00000146049 | ENSG00000146049 | -1.18517 | 2.188712 | -6.60303 | 1.40E-10 | 1.51E-08 | 13.68 | KAAG1 |
| ENSG00000060566 | ENSG00000060566 | -1.18296 | 7.205537 | -3.70392 | 0.000245 | 0.001153 | 0.0687 | CREB3L3 |
| ENSG00000182901 | ENSG00000182901 | -1.18232 | 4.180575 | -4.54545 | 7.43E-06 | 7.29E-05 | 3.324922 | RGS7 |
| ENSG00000158764 | ENSG00000158764 | -1.1816 | 3.014649 | -4.63298 | 5.00E-06 | 5.40E-05 | 3.697925 | ITLN2 |
| ENSG00000204099 | ENSG00000204099 | -1.18141 | 5.45379 | -4.59849 | 5.85E-06 | 6.09E-05 | 3.550174 | NEU4 |
| ENSG00000153294 | ENSG00000153294 | -1.18036 | 6.447266 | -4.54595 | 7.41E-06 | 7.28E-05 | 3.327022 | ADGRF4 |
| ENSG00000187566 | ENSG00000187566 | -1.18001 | 6.452304 | -6.73671 | 6.19E-11 | 7.98E-09 | 14.46035 | NHLRC1 |
| ENSG00000198822 | ENSG00000198822 | -1.17978 | 2.842573 | -6.40455 | 4.57E-10 | 3.79E-08 | 12.54418 | GRM3 |
| ENSG00000122584 | ENSG00000122584 | -1.17957 | 1.877532 | -6.2098 | 1.42E-09 | 9.16E-08 | 11.45659 | NXPH1 |
| ENSG00000212901 | ENSG00000212901 | -1.17843 | 1.469949 | -4.84583 | 1.86E-06 | 2.47E-05 | 4.631281 | KRTAP3-1 |
| ENSG00000087495 | ENSG00000087495 | -1.17776 | 5.708103 | -4.89877 | 1.44E-06 | 2.04E-05 | 4.86916 | PHACTR3 |
| ENSG00000248461 | ENSG00000248461 | -1.17734 | 1.672168 | -4.82682 | 2.03E-06 | 2.66E-05 | 4.546432 | LINC02119 |
| ENSG00000281706 | ENSG00000281706 | -1.17654 | 5.443711 | -7.33487 | 1.41E-12 | 3.81E-10 | 18.09794 | LINC01012 |
| ENSG00000182747 | ENSG00000182747 | -1.17585 | 4.364932 | -3.99386 | 7.84E-05 | 0.000468 | 1.122054 | SLC35D3 |
| ENSG00000099399 | ENSG00000099399 | -1.17395 | 2.127469 | -3.79082 | 0.000175 | 0.000883 | 0.376737 | MAGEB2 |
| ENSG00000183837 | ENSG00000183837 | -1.17341 | 4.173633 | -4.84055 | 1.90E-06 | 2.52E-05 | 4.607687 | PNMA3 |
| ENSG00000256162 | ENSG00000256162 | -1.17291 | 3.359853 | -4.52295 | 8.22E-06 | 7.89E-05 | 3.230057 | SMLR1 |
| ENSG00000109101 | ENSG00000109101 | -1.17236 | 3.739977 | -5.26103 | 2.43E-07 | 5.05E-06 | 6.557071 | FOXN1 |
| ENSG00000106031 | ENSG00000106031 | -1.17174 | 9.152175 | -3.58328 | 0.000385 | 0.001655 | -0.34793 | HOXA13 |
| ENSG00000100665 | ENSG00000100665 | -1.1715 | 7.973545 | -3.67374 | 0.000274 | 0.001264 | -0.03674 | SERPINA4 |
| ENSG00000185290 | ENSG00000185290 | -1.17089 | 2.813502 | -4.00277 | 7.56E-05 | 0.000455 | 1.155603 | NUPR2 |
| ENSG00000159409 | ENSG00000159409 | -1.17045 | 4.824579 | -4.59963 | 5.82E-06 | 6.07E-05 | 3.555073 | CELF3 |
| ENSG00000151838 | ENSG00000151838 | -1.16997 | 2.088446 | -5.9266 | 7.07E-09 | 3.22E-07 | 9.923774 | CCDC175 |
| ENSG00000168631 | ENSG00000168631 | -1.16971 | 10.1245 | -2.70757 | 0.007091 | 0.017003 | -2.98108 | MUCL3 |
| ENSG00000242599 | ENSG00000242599 | -1.16925 | 1.374802 | -5.2385 | 2.72E-07 | 5.51E-06 | 6.449031 | CSAG4 |
| ENSG00000158014 | ENSG00000158014 | -1.16896 | 4.550219 | -4.80142 | 2.29E-06 | 2.91E-05 | 4.433505 | SLC30A2 |
| ENSG00000184647 | ENSG00000184647 | -1.16746 | 2.2387 | -6.22558 | 1.30E-09 | 8.49E-08 | 11.54375 | PRSS55 |
| ENSG00000121075 | ENSG00000121075 | -1.16662 | 3.835864 | -4.44895 | 1.14E-05 | 0.000102 | 2.92104 | TBX4 |
| ENSG00000081277 | ENSG00000081277 | -1.16645 | 6.387523 | -3.53665 | 0.000457 | 0.001897 | -0.50554 | PKP1 |
| ENSG00000224141 | ENSG00000224141 | -1.16619 | 1.606791 | -4.71459 | 3.43E-06 | 4.00E-05 | 4.051406 | MIR548XHG |
| ENSG00000165828 | ENSG00000165828 | -1.16578 | 9.57201 | -3.4941 | 0.000533 | 0.002148 | -0.64767 | PRAP1 |
| ENSG00000088340 | ENSG00000088340 | -1.16559 | 9.697049 | -5.44106 | 9.64E-08 | 2.48E-06 | 7.434308 | FER1L4 |
| ENSG00000156096 | ENSG00000156096 | -1.16377 | 1.705551 | -5.41692 | 1.09E-07 | 2.74E-06 | 7.315223 | UGT2B4 |
| ENSG00000011083 | ENSG00000011083 | -1.16352 | 4.69216 | -4.79671 | 2.34E-06 | 2.96E-05 | 4.412611 | SLC6A7 |
| ENSG00000178171 | ENSG00000178171 | -1.16219 | 3.114646 | -4.61676 | 5.38E-06 | 5.71E-05 | 3.628341 | AMER3 |
| ENSG00000244342 | ENSG00000244342 | -1.16033 | 1.949562 | -7.344 | 1.33E-12 | 3.70E-10 | 18.15526 | LINC00698 |
| ENSG00000198221 | ENSG00000198221 | -1.15925 | 6.155094 | -7.66799 | 1.55E-13 | 6.90E-11 | 20.2231 | AFDN-DT |
| ENSG00000165621 | ENSG00000165621 | -1.15922 | 3.047259 | -4.99124 | 9.24E-07 | 1.44E-05 | 5.290069 | OXGR1 |
| ENSG00000264424 | ENSG00000264424 | -1.15593 | 2.362171 | -5.07759 | 6.06E-07 | 1.04E-05 | 5.689303 | MYH4 |
| ENSG00000157703 | ENSG00000157703 | -1.15578 | 3.713428 | -5.61249 | 3.91E-08 | 1.21E-06 | 8.29293 | SVOPL |
| ENSG00000212916 | ENSG00000212916 | -1.15574 | 7.4652 | -7.43491 | 7.30E-13 | 2.27E-10 | 18.7289 | MAP10 |
| ENSG00000125999 | ENSG00000125999 | -1.15539 | 9.795995 | -2.54945 | 0.011191 | 0.024587 | -3.38179 | BPIFB1 |
| ENSG00000253293 | ENSG00000253293 | -1.15484 | 8.813438 | -4.2026 | 3.31E-05 | 0.000238 | 1.925391 | HOXA10 |
| ENSG00000203688 | ENSG00000203688 | -1.15433 | 4.919299 | -5.12129 | 4.89E-07 | 8.77E-06 | 5.893587 | LINC02487 |
| ENSG00000241359 | ENSG00000241359 | -1.15428 | 4.229285 | -5.6762 | 2.78E-08 | 9.21E-07 | 8.617743 | SYNPR-AS1 |
| ENSG00000113249 | ENSG00000113249 | -1.15408 | 4.867492 | -3.91018 | 0.00011 | 0.000612 | 0.810534 | HAVCR1 |
| ENSG00000170775 | ENSG00000170775 | -1.15354 | 5.676137 | -5.08962 | 5.71E-07 | 9.89E-06 | 5.7454 | GPR37 |
| ENSG00000255501 | ENSG00000255501 | -1.15202 | 2.027705 | -4.80331 | 2.27E-06 | 2.89E-05 | 4.441873 | CARD18 |
| ENSG00000124249 | ENSG00000124249 | -1.15067 | 7.58501 | -4.13895 | 4.32E-05 | 0.000294 | 1.676489 | KCNK15 |
| ENSG00000178201 | ENSG00000178201 | -1.15025 | 5.400996 | -7.32409 | 1.51E-12 | 4.03E-10 | 18.03033 | VN1R1 |
| ENSG00000108576 | ENSG00000108576 | -1.14977 | 4.976276 | -6.53913 | 2.05E-10 | 2.06E-08 | 13.31134 | SLC6A4 |
| ENSG00000225210 | ENSG00000225210 | -1.1493 | 5.032912 | -5.88681 | 8.81E-09 | 3.78E-07 | 9.713158 | DUXAP9 |
| ENSG00000196335 | ENSG00000196335 | -1.14785 | 7.484617 | -5.34033 | 1.62E-07 | 3.70E-06 | 6.940394 | STK31 |
| ENSG00000091583 | ENSG00000091583 | -1.14716 | 4.181797 | -3.93071 | 0.000101 | 0.000573 | 0.886416 | APOH |
| ENSG00000141449 | ENSG00000141449 | -1.1471 | 6.396126 | -4.96394 | 1.05E-06 | 1.59E-05 | 5.165055 | GREB1L |
| ENSG00000230445 | ENSG00000230445 | -1.14699 | 6.191623 | -5.86945 | 9.70E-09 | 4.06E-07 | 9.621589 | LRRC37A6P |
| ENSG00000267327 | ENSG00000267327 | -1.14683 | 1.76038 | -5.76536 | 1.72E-08 | 6.35E-07 | 9.077406 | LOC107985164 |
| ENSG00000198734 | ENSG00000198734 | -1.14645 | 10.42344 | -4.56031 | 6.95E-06 | 6.93E-05 | 3.387785 | F5 |
| ENSG00000184956 | ENSG00000184956 | -1.14535 | 11.15072 | -2.49276 | 0.01311 | 0.028001 | -3.51978 | MUC6 |
| ENSG00000269821 | ENSG00000269821 | -1.14481 | 10.86111 | -7.55794 | 3.23E-13 | 1.19E-10 | 19.51344 | KCNQ1OT1 |
| ENSG00000234840 | ENSG00000234840 | -1.1413 | 3.837957 | -4.59192 | 6.02E-06 | 6.24E-05 | 3.522153 | LINC01239 |
| ENSG00000214814 | ENSG00000214814 | -1.14105 | 8.420481 | -3.21747 | 0.001407 | 0.004646 | -1.53223 | FER1L6 |
| ENSG00000232775 | ENSG00000232775 | -1.13954 | 2.313473 | -5.91596 | 7.50E-09 | 3.35E-07 | 9.867344 | BMS1P22 |
| ENSG00000204889 | ENSG00000204889 | -1.13871 | 2.49366 | -4.39576 | 1.44E-05 | 0.000124 | 2.701713 | KRT40 |
| ENSG00000244791 | ENSG00000244791 | -1.13792 | 1.740446 | -7.21401 | 3.08E-12 | 7.12E-10 | 17.34413 | LOC101927657 |
| ENSG00000197753 | ENSG00000197753 | -1.13764 | 3.373191 | -6.14175 | 2.10E-09 | 1.24E-07 | 11.083 | LHFPL5 |
| ENSG00000241832 | ENSG00000241832 | -1.13744 | 1.600463 | -6.44834 | 3.52E-10 | 3.15E-08 | 12.79244 | CECR3 |
| ENSG00000181392 | ENSG00000181392 | -1.13683 | 7.006387 | -5.04711 | 7.04E-07 | 1.16E-05 | 5.547699 | SYNE4 |
| ENSG00000189325 | ENSG00000189325 | -1.13593 | 9.977636 | -5.04262 | 7.20E-07 | 1.18E-05 | 5.526898 | BNIP5 |
| ENSG00000215529 | ENSG00000215529 | -1.13498 | 3.981809 | -6.15389 | 1.96E-09 | 1.17E-07 | 11.14937 | EFCAB8 |
| ENSG00000228630 | ENSG00000228630 | -1.13384 | 6.133787 | -4.17332 | 3.74E-05 | 0.000262 | 1.810445 | HOTAIR |
| ENSG00000238062 | ENSG00000238062 | -1.13361 | 4.262504 | -6.55612 | 1.85E-10 | 1.91E-08 | 13.40906 | SPATA3-AS1 |
| ENSG00000127252 | ENSG00000127252 | -1.13344 | 3.842475 | -4.81204 | 2.18E-06 | 2.80E-05 | 4.480628 | PLAAT1 |
| ENSG00000161652 | ENSG00000161652 | -1.1334 | 2.689471 | -5.80244 | 1.40E-08 | 5.35E-07 | 9.270355 | IZUMO2 |
| ENSG00000111981 | ENSG00000111981 | -1.13304 | 5.106394 | -5.25539 | 2.50E-07 | 5.16E-06 | 6.529997 | ULBP1 |
| ENSG00000101004 | ENSG00000101004 | -1.13242 | 9.302268 | -6.46663 | 3.16E-10 | 2.92E-08 | 12.89648 | NINL |
| ENSG00000204460 | ENSG00000204460 | -1.13189 | 1.190735 | -5.58904 | 4.43E-08 | 1.34E-06 | 8.174145 | LINC01854 |
| ENSG00000158553 | ENSG00000158553 | -1.13187 | 1.921217 | -6.3958 | 4.81E-10 | 3.90E-08 | 12.49472 | POM121L2 |
| ENSG00000128655 | ENSG00000128655 | -1.13167 | 7.11825 | -5.84949 | 1.08E-08 | 4.41E-07 | 9.516599 | PDE11A |
| ENSG00000147606 | ENSG00000147606 | -1.13134 | 4.450635 | -5.94257 | 6.47E-09 | 3.00E-07 | 10.0087 | SLC26A7 |
| ENSG00000163817 | ENSG00000163817 | -1.13033 | 9.689994 | -4.04671 | 6.32E-05 | 0.000395 | 1.321919 | SLC6A20 |
| ENSG00000180767 | ENSG00000180767 | -1.13024 | 6.723216 | -4.9324 | 1.23E-06 | 1.79E-05 | 5.021452 | CHST13 |
| ENSG00000134365 | ENSG00000134365 | -1.13005 | 1.709368 | -5.59499 | 4.29E-08 | 1.31E-06 | 8.204245 | CFHR4 |
| ENSG00000180806 | ENSG00000180806 | -1.12958 | 6.94751 | -5.18554 | 3.55E-07 | 6.77E-06 | 6.196724 | HOXC9 |
| ENSG00000240922 | ENSG00000240922 | -1.12919 | 2.639688 | -5.81404 | 1.32E-08 | 5.11E-07 | 9.330892 | LSAMP-AS1 |
| ENSG00000230524 | ENSG00000230524 | -1.12879 | 3.872693 | -7.28433 | 1.95E-12 | 4.94E-10 | 17.78156 | COL6A4P1 |
| ENSG00000196289 | ENSG00000196289 | -1.12814 | 1.805287 | -6.97144 | 1.44E-11 | 2.55E-09 | 15.85974 | BECN2 |
| ENSG00000185156 | ENSG00000185156 | -1.12792 | 6.413787 | -4.60662 | 5.63E-06 | 5.93E-05 | 3.584928 | MFSD6L |
| ENSG00000236824 | ENSG00000236824 | -1.12786 | 9.357233 | -5.30496 | 1.94E-07 | 4.25E-06 | 6.768784 | BCYRN1 |
| ENSG00000164761 | ENSG00000164761 | -1.12648 | 8.481914 | -5.24098 | 2.69E-07 | 5.46E-06 | 6.460922 | TNFRSF11B |
| ENSG00000170689 | ENSG00000170689 | -1.12593 | 8.994137 | -4.3452 | 1.80E-05 | 0.000147 | 2.495487 | HOXB9 |
| ENSG00000096395 | ENSG00000096395 | -1.12581 | 1.94193 | -4.30207 | 2.17E-05 | 0.000171 | 2.321236 | MLN |
| ENSG00000150750 | ENSG00000150750 | -1.12568 | 6.308943 | -4.48704 | 9.65E-06 | 8.93E-05 | 3.079511 | C11orf53 |
| ENSG00000205634 | ENSG00000205634 | -1.1253 | 1.519597 | -6.01868 | 4.22E-09 | 2.15E-07 | 10.41577 | LINC00898 |
| ENSG00000207923 | ENSG00000207923 | -1.12422 | 3.112493 | -6.88668 | 2.45E-11 | 3.93E-09 | 15.35017 | MIR559 |
| ENSG00000124490 | ENSG00000124490 | -1.1239 | 1.784326 | -4.50419 | 8.94E-06 | 8.43E-05 | 3.151273 | CRISP2 |
| ENSG00000163283 | ENSG00000163283 | -1.12363 | 5.237709 | -3.40133 | 0.000744 | 0.002795 | -0.95192 | ALPP |
| ENSG00000163631 | ENSG00000163631 | -1.12351 | 4.15708 | -3.19301 | 0.001528 | 0.004954 | -1.60715 | ALB |
| ENSG00000182256 | ENSG00000182256 | -1.12336 | 2.684064 | -4.6633 | 4.35E-06 | 4.84E-05 | 3.82863 | GABRG3 |
| ENSG00000142609 | ENSG00000142609 | -1.12305 | 4.733286 | -5.94525 | 6.37E-09 | 2.96E-07 | 10.02295 | CFAP74 |
| ENSG00000150556 | ENSG00000150556 | -1.12147 | 8.58746 | -4.4973 | 9.22E-06 | 8.63E-05 | 3.122398 | LYPD6B |
| ENSG00000106927 | ENSG00000106927 | -1.12126 | 5.737624 | -4.93472 | 1.21E-06 | 1.78E-05 | 5.031992 | AMBP |
| ENSG00000261488 | ENSG00000261488 | -1.1198 | 6.299357 | -7.15397 | 4.53E-12 | 9.75E-10 | 16.97315 | TBILA |
| ENSG00000173212 | ENSG00000173212 | -1.11911 | 3.690938 | -5.01701 | 8.15E-07 | 1.30E-05 | 5.408567 | MAB21L3 |
| ENSG00000129654 | ENSG00000129654 | -1.11856 | 7.372685 | -3.12592 | 0.001912 | 0.005918 | -1.80982 | FOXJ1 |
| ENSG00000105675 | ENSG00000105675 | -1.11808 | 4.079168 | -3.18045 | 0.001594 | 0.005121 | -1.64538 | ATP4A |
| ENSG00000167037 | ENSG00000167037 | -1.11717 | 7.920734 | -6.29864 | 8.50E-10 | 6.08E-08 | 11.94939 | SGSM1 |
| ENSG00000248498 | ENSG00000248498 | -1.11651 | 1.683167 | -5.22331 | 2.94E-07 | 5.84E-06 | 6.37645 | ASNSP1 |
| ENSG00000183117 | ENSG00000183117 | -1.11555 | 4.094506 | -4.16661 | 3.85E-05 | 0.000268 | 1.784214 | CSMD1 |
| ENSG00000055732 | ENSG00000055732 | -1.11437 | 7.140433 | -5.37721 | 1.34E-07 | 3.19E-06 | 7.120325 | MCOLN3 |
| ENSG00000229494 | ENSG00000229494 | -1.11392 | 1.211377 | -7.1586 | 4.40E-12 | 9.50E-10 | 17.00168 | LOC101927948 |
| ENSG00000226995 | ENSG00000226995 | -1.11371 | 1.660641 | -6.43798 | 3.75E-10 | 3.28E-08 | 12.73356 | LINC00658 |
| ENSG00000133488 | ENSG00000133488 | -1.11356 | 5.382012 | -3.85705 | 0.000135 | 0.000722 | 0.615923 | SEC14L4 |
| ENSG00000005073 | ENSG00000005073 | -1.11352 | 6.682488 | -3.61702 | 0.000339 | 0.001499 | -0.23271 | HOXA11 |
| ENSG00000203995 | ENSG00000203995 | -1.11236 | 4.773491 | -5.24305 | 2.66E-07 | 5.42E-06 | 6.470855 | ZYG11A |
| ENSG00000197893 | ENSG00000197893 | -1.11181 | 3.943945 | -5.32718 | 1.73E-07 | 3.89E-06 | 6.876466 | NRAP |
| ENSG00000171431 | ENSG00000171431 | -1.1106 | 8.682454 | -2.52134 | 0.012109 | 0.026241 | -3.4506 | KRT20 |
| ENSG00000249267 | ENSG00000249267 | -1.11013 | 4.433684 | -4.77502 | 2.59E-06 | 3.20E-05 | 4.316649 | LINC00939 |
| ENSG00000203857 | ENSG00000203857 | -1.1101 | 1.392837 | -5.65789 | 3.07E-08 | 9.94E-07 | 8.524073 | HSD3B1 |
| ENSG00000145692 | ENSG00000145692 | -1.10948 | 3.713916 | -4.91212 | 1.35E-06 | 1.93E-05 | 4.929506 | BHMT |
| ENSG00000148584 | ENSG00000148584 | -1.10808 | 8.39363 | -3.64225 | 0.000309 | 0.001392 | -0.14588 | A1CF |
| ENSG00000179059 | ENSG00000179059 | -1.10803 | 1.737662 | -4.53984 | 7.62E-06 | 7.43E-05 | 3.301203 | ZFP42 |
| ENSG00000103485 | ENSG00000103485 | -1.1055 | 9.256754 | -4.9857 | 9.49E-07 | 1.47E-05 | 5.26466 | QPRT |
| ENSG00000273079 | ENSG00000273079 | -1.10519 | 4.893342 | -4.72139 | 3.33E-06 | 3.90E-05 | 4.081124 | GRIN2B |
| ENSG00000009765 | ENSG00000009765 | -1.10503 | 9.590639 | -4.23725 | 2.86E-05 | 0.000213 | 2.062321 | IYD |
| ENSG00000166426 | ENSG00000166426 | -1.105 | 4.523961 | -3.16949 | 0.001654 | 0.005272 | -1.67865 | CRABP1 |
| ENSG00000163218 | ENSG00000163218 | -1.10492 | 3.181566 | -4.66341 | 4.35E-06 | 4.84E-05 | 3.829099 | PGLYRP4 |
| ENSG00000187172 | ENSG00000187172 | -1.10491 | 1.916082 | -4.63239 | 5.01E-06 | 5.41E-05 | 3.695392 | BAGE2 |
| ENSG00000166796 | ENSG00000166796 | -1.10392 | 3.657092 | -4.18324 | 3.59E-05 | 0.000253 | 1.849303 | LDHC |
| ENSG00000279078 | ENSG00000279078 | -1.10165 | 6.462877 | -7.87885 | 3.70E-14 | 2.05E-11 | 21.60335 | SND1-IT1 |
| ENSG00000228784 | ENSG00000228784 | -1.10154 | 5.611403 | -5.7129 | 2.28E-08 | 7.98E-07 | 8.806213 | LINC00954 |
| ENSG00000179520 | ENSG00000179520 | -1.10018 | 2.27892 | -4.81403 | 2.16E-06 | 2.78E-05 | 4.489466 | SLC17A8 |
| ENSG00000166183 | ENSG00000166183 | -1.09993 | 5.351082 | -5.30868 | 1.91E-07 | 4.18E-06 | 6.786778 | ASPG |
| ENSG00000225868 | ENSG00000225868 | -1.09942 | 2.77227 | -5.62133 | 3.73E-08 | 1.17E-06 | 8.337807 | LOC100631378 |
| ENSG00000262943 | ENSG00000262943 | -1.09878 | 5.616938 | -5.22316 | 2.94E-07 | 5.85E-06 | 6.375745 | ALOX12P2 |
| ENSG00000125888 | ENSG00000125888 | -1.09785 | 1.842002 | -5.4017 | 1.18E-07 | 2.89E-06 | 7.240359 | BANF2 |
| ENSG00000188833 | ENSG00000188833 | -1.09745 | 8.021025 | -4.26956 | 2.49E-05 | 0.000191 | 2.19092 | ENTPD8 |
| ENSG00000197472 | ENSG00000197472 | -1.09702 | 6.66627 | -6.40554 | 4.54E-10 | 3.78E-08 | 12.5498 | ZNF695 |
| ENSG00000196876 | ENSG00000196876 | -1.09567 | 7.667158 | -6.00991 | 4.44E-09 | 2.23E-07 | 10.36864 | SCN8A |
| ENSG00000243955 | ENSG00000243955 | -1.09485 | 8.222679 | -3.08492 | 0.002189 | 0.006592 | -1.93168 | GSTA1 |
| ENSG00000129151 | ENSG00000129151 | -1.09353 | 4.351736 | -5.48616 | 7.62E-08 | 2.05E-06 | 7.658038 | BBOX1 |
| ENSG00000181690 | ENSG00000181690 | -1.09288 | 7.70538 | -6.57242 | 1.68E-10 | 1.77E-08 | 13.50306 | PLAG1 |
| ENSG00000173805 | ENSG00000173805 | -1.09256 | 5.084838 | -5.59473 | 4.30E-08 | 1.31E-06 | 8.202926 | HAP1 |
| ENSG00000182223 | ENSG00000182223 | -1.09051 | 1.642145 | -6.0405 | 3.74E-09 | 1.95E-07 | 10.53324 | ZAR1 |
| ENSG00000148123 | ENSG00000148123 | -1.08871 | 3.891964 | -4.40945 | 1.36E-05 | 0.000118 | 2.757962 | PLPPR1 |
| ENSG00000180316 | ENSG00000180316 | -1.08869 | 3.973754 | -6.14004 | 2.12E-09 | 1.25E-07 | 11.07362 | PNPLA1 |
| ENSG00000167612 | ENSG00000167612 | -1.08804 | 2.410687 | -4.83966 | 1.91E-06 | 2.53E-05 | 4.603707 | ANKRD33 |
| ENSG00000256115 | ENSG00000256115 | -1.0877 | 0.971367 | -7.7894 | 6.81E-14 | 3.35E-11 | 21.01457 | LINC02443 |
| ENSG00000249158 | ENSG00000249158 | -1.08717 | 3.493251 | -3.85468 | 0.000137 | 0.000728 | 0.607298 | PCDHA11 |
| ENSG00000137948 | ENSG00000137948 | -1.08711 | 2.864586 | -5.239 | 2.71E-07 | 5.50E-06 | 6.451443 | BRDT |
| ENSG00000235097 | ENSG00000235097 | -1.0865 | 2.254879 | -5.34671 | 1.57E-07 | 3.61E-06 | 6.971435 | LINC00330 |
| ENSG00000113492 | ENSG00000113492 | -1.08629 | 1.918174 | -5.45228 | 9.09E-08 | 2.37E-06 | 7.489812 | AGXT2 |
| ENSG00000009950 | ENSG00000009950 | -1.08551 | 9.212192 | -4.21147 | 3.19E-05 | 0.000231 | 1.960344 | MLXIPL |
| ENSG00000145808 | ENSG00000145808 | -1.08479 | 2.536082 | -4.53294 | 7.86E-06 | 7.61E-05 | 3.272101 | ADAMTS19 |
| ENSG00000007350 | ENSG00000007350 | -1.08415 | 4.148365 | -3.41853 | 0.000699 | 0.002665 | -0.89611 | TKTL1 |
| ENSG00000133640 | ENSG00000133640 | -1.08322 | 4.240431 | -4.4504 | 1.13E-05 | 0.000102 | 2.927013 | LRRIQ1 |
| ENSG00000172139 | ENSG00000172139 | -1.08268 | 5.03287 | -7.29845 | 1.78E-12 | 4.66E-10 | 17.86977 | SLC9C1 |
| ENSG00000149634 | ENSG00000149634 | -1.08229 | 5.251977 | -7.66566 | 1.57E-13 | 6.90E-11 | 20.208 | SPATA25 |
| ENSG00000138759 | ENSG00000138759 | -1.08183 | 10.03919 | -4.96935 | 1.03E-06 | 1.56E-05 | 5.189782 | FRAS1 |
| ENSG00000144852 | ENSG00000144852 | -1.08161 | 8.249627 | -3.43702 | 0.000655 | 0.002528 | -0.8358 | NR1I2 |
| ENSG00000230666 | ENSG00000230666 | -1.08149 | 3.002229 | -6.62479 | 1.22E-10 | 1.36E-08 | 13.80618 | CEACAM22P |
| ENSG00000273274 | ENSG00000273274 | -1.08128 | 3.373903 | -6.13348 | 2.20E-09 | 1.29E-07 | 11.03782 | ZBTB8B |
| ENSG00000241598 | ENSG00000241598 | -1.08126 | 2.619988 | -5.54928 | 5.47E-08 | 1.60E-06 | 7.973739 | KRTAP5-4 |
| ENSG00000169994 | ENSG00000169994 | -1.08082 | 10.9627 | -3.90083 | 0.000114 | 0.00063 | 0.776122 | MYO7B |
| ENSG00000145626 | ENSG00000145626 | -1.08055 | 1.543254 | -4.94727 | 1.14E-06 | 1.70E-05 | 5.089074 | UGT3A1 |
| ENSG00000198944 | ENSG00000198944 | -1.08032 | 8.70191 | -4.96222 | 1.06E-06 | 1.61E-05 | 5.15723 | SOWAHA |
| ENSG00000121742 | ENSG00000121742 | -1.07987 | 4.358657 | -3.71058 | 0.000238 | 0.00113 | 0.092064 | GJB6 |
| ENSG00000099769 | ENSG00000099769 | -1.07955 | 6.035557 | -3.80217 | 0.000168 | 0.000854 | 0.417456 | IGFALS |
| ENSG00000249816 | ENSG00000249816 | -1.0794 | 2.128371 | -5.80086 | 1.41E-08 | 5.39E-07 | 9.262112 | LINC00964 |
| ENSG00000188373 | ENSG00000188373 | -1.07892 | 2.870528 | -3.15424 | 0.00174 | 0.005496 | -1.72475 | C10orf99 |
| ENSG00000104044 | ENSG00000104044 | -1.07873 | 4.237733 | -3.86066 | 0.000133 | 0.000714 | 0.629043 | OCA2 |
| ENSG00000146678 | ENSG00000146678 | -1.07661 | 4.31941 | -3.59877 | 0.000363 | 0.001584 | -0.29518 | IGFBP1 |
| ENSG00000123569 | ENSG00000123569 | -1.07541 | 0.962102 | -6.27378 | 9.82E-10 | 6.77E-08 | 11.81092 | H2BW1 |
| ENSG00000148734 | ENSG00000148734 | -1.07486 | 6.466748 | -5.83087 | 1.20E-08 | 4.77E-07 | 9.418938 | NPFFR1 |
| ENSG00000186973 | ENSG00000186973 | -1.07468 | 3.275841 | -4.7446 | 2.99E-06 | 3.58E-05 | 4.182765 | FAM183A |
| ENSG00000184454 | ENSG00000184454 | -1.07453 | 7.485189 | -5.21802 | 3.01E-07 | 5.97E-06 | 6.351214 | NCMAP |
| ENSG00000179299 | ENSG00000179299 | -1.07421 | 7.873216 | -5.62876 | 3.58E-08 | 1.13E-06 | 8.375593 | NSUN7 |
| ENSG00000142700 | ENSG00000142700 | -1.0733 | 3.061497 | -3.72142 | 0.000229 | 0.001092 | 0.130197 | DMRTA2 |
| ENSG00000149927 | ENSG00000149927 | -1.07308 | 5.547364 | -5.30735 | 1.92E-07 | 4.21E-06 | 6.780342 | DOC2A |
| ENSG00000146910 | ENSG00000146910 | -1.07247 | 1.826501 | -5.60581 | 4.05E-08 | 1.25E-06 | 8.259073 | CNPY1 |
| ENSG00000259974 | ENSG00000259974 | -1.07241 | 8.386813 | -3.36146 | 0.000856 | 0.003133 | -1.08036 | LINC00261 |
| ENSG00000196436 | ENSG00000196436 | -1.07075 | 6.654406 | -4.32989 | 1.92E-05 | 0.000155 | 2.433432 | NPIPB15 |
| ENSG00000229261 | ENSG00000229261 | -1.06994 | 3.887034 | -6.27351 | 9.84E-10 | 6.77E-08 | 11.80945 | LOC101928994 |
| ENSG00000188817 | ENSG00000188817 | -1.06891 | 2.854043 | -5.97263 | 5.47E-09 | 2.66E-07 | 10.16896 | SNTN |
| ENSG00000072657 | ENSG00000072657 | -1.06858 | 6.321737 | -4.36669 | 1.64E-05 | 0.000137 | 2.582873 | TRHDE |
| ENSG00000184925 | ENSG00000184925 | -1.06846 | 6.138371 | -5.96346 | 5.76E-09 | 2.76E-07 | 10.11998 | LCN12 |
| ENSG00000233718 | ENSG00000233718 | -1.0682 | 2.760594 | -5.56063 | 5.15E-08 | 1.52E-06 | 8.030822 | MYCNOS |
| ENSG00000248599 | ENSG00000248599 | -1.06784 | 2.054311 | -5.92341 | 7.20E-09 | 3.26E-07 | 9.906872 | FLJ42969 |
| ENSG00000101850 | ENSG00000101850 | -1.06712 | 5.460729 | -4.22021 | 3.07E-05 | 0.000225 | 1.994828 | GPR143 |
| ENSG00000127325 | ENSG00000127325 | -1.06648 | 3.189228 | -4.73736 | 3.09E-06 | 3.68E-05 | 4.151019 | BEST3 |
| ENSG00000135773 | ENSG00000135773 | -1.0663 | 7.084701 | -3.7792 | 0.000183 | 0.000916 | 0.335135 | CAPN9 |
| ENSG00000187569 | ENSG00000187569 | -1.06562 | 1.647591 | -4.85773 | 1.75E-06 | 2.37E-05 | 4.684572 | DPPA3 |
| ENSG00000148965 | ENSG00000148965 | -1.0646 | 2.875503 | -4.40695 | 1.37E-05 | 0.000119 | 2.747664 | SAA4 |
| ENSG00000183914 | ENSG00000183914 | -1.06433 | 7.22742 | -5.30727 | 1.92E-07 | 4.21E-06 | 6.779978 | DNAH2 |
| ENSG00000250420 | ENSG00000250420 | -1.06407 | 3.381229 | -3.87084 | 0.000128 | 0.000693 | 0.666182 | AACSP1 |
| ENSG00000268902 | ENSG00000268902 | -1.06261 | 2.35378 | -3.73616 | 0.000216 | 0.001044 | 0.182215 | CSAG2 |
| ENSG00000127249 | ENSG00000127249 | -1.06214 | 6.741248 | -4.02166 | 7.00E-05 | 0.000428 | 1.226909 | ATP13A4 |
| ENSG00000255367 | ENSG00000255367 | -1.06195 | 5.54179 | -5.20926 | 3.15E-07 | 6.20E-06 | 6.309463 | LOC101927708 |
| ENSG00000251629 | ENSG00000251629 | -1.06134 | 1.697781 | -4.44951 | 1.14E-05 | 0.000102 | 2.923346 | LINC02241 |
| ENSG00000158486 | ENSG00000158486 | -1.06125 | 7.049701 | -7.40749 | 8.75E-13 | 2.66E-10 | 18.55534 | DNAH3 |
| ENSG00000266200 | ENSG00000266200 | -1.06113 | 4.451373 | -3.16574 | 0.001675 | 0.005324 | -1.69002 | PNLIPRP2 |
| ENSG00000023839 | ENSG00000023839 | -1.06025 | 7.211041 | -4.41076 | 1.35E-05 | 0.000117 | 2.763321 | ABCC2 |
| ENSG00000272568 | ENSG00000272568 | -1.05969 | 5.294266 | -5.15092 | 4.22E-07 | 7.80E-06 | 6.032996 | LOC100506497 |
| ENSG00000234722 | ENSG00000234722 | -1.05949 | 2.197003 | -4.7078 | 3.54E-06 | 4.11E-05 | 4.021808 | LINC01287 |
| ENSG00000124102 | ENSG00000124102 | -1.05919 | 10.91496 | -3.15321 | 0.001746 | 0.00551 | -1.72788 | PI3 |
| ENSG00000167798 | ENSG00000167798 | -1.05844 | 4.628356 | -3.39447 | 0.000762 | 0.00285 | -0.97412 | C3P1 |
| ENSG00000186529 | ENSG00000186529 | -1.0583 | 9.337245 | -5.00702 | 8.56E-07 | 1.35E-05 | 5.362589 | CYP4F3 |
| ENSG00000207340 | ENSG00000207340 | -1.05793 | 2.754673 | -7.25526 | 2.36E-12 | 5.76E-10 | 17.60033 | RNVU1-1 |
| ENSG00000113073 | ENSG00000113073 | -1.05788 | 3.982154 | -6.85146 | 3.05E-11 | 4.61E-09 | 15.13979 | SLC4A9 |
| ENSG00000251350 | ENSG00000251350 | -1.05771 | 3.015037 | -3.93754 | 9.83E-05 | 0.000561 | 0.911749 | LINC02475 |
| ENSG00000268606 | ENSG00000268606 | -1.05676 | 1.439984 | -5.16764 | 3.88E-07 | 7.30E-06 | 6.111968 | MAGEA2 |
| ENSG00000112280 | ENSG00000112280 | -1.05658 | 7.353559 | -4.40932 | 1.36E-05 | 0.000118 | 2.757416 | COL9A1 |
| ENSG00000180305 | ENSG00000180305 | -1.05615 | 1.368677 | -6.44764 | 3.54E-10 | 3.16E-08 | 12.78843 | WFDC10A |
| ENSG00000237463 | ENSG00000237463 | -1.05537 | 1.837645 | -5.90643 | 7.91E-09 | 3.49E-07 | 9.816856 | LRRC52-AS1 |
| ENSG00000206195 | ENSG00000206195 | -1.05526 | 7.632787 | -5.28563 | 2.14E-07 | 4.56E-06 | 6.67546 | DUXAP8 |
| ENSG00000144395 | ENSG00000144395 | -1.05374 | 7.170528 | -8.11837 | 7.04E-15 | 6.04E-12 | 23.20323 | CCDC150 |
| ENSG00000248265 | ENSG00000248265 | -1.05326 | 3.990659 | -5.81555 | 1.30E-08 | 5.07E-07 | 9.33877 | FLJ12825 |
| ENSG00000169906 | ENSG00000169906 | -1.05188 | 2.674517 | -6.28811 | 9.04E-10 | 6.39E-08 | 11.89071 | S100G |
| ENSG00000161905 | ENSG00000161905 | -1.0518 | 4.857226 | -4.22303 | 3.04E-05 | 0.000223 | 2.005975 | ALOX15 |
| ENSG00000141485 | ENSG00000141485 | -1.05172 | 4.687623 | -4.33787 | 1.86E-05 | 0.000151 | 2.465737 | SLC13A5 |
| ENSG00000236345 | ENSG00000236345 | -1.05156 | 3.127418 | -4.98046 | 9.74E-07 | 1.50E-05 | 5.240635 | SCAT8 |
| ENSG00000204792 | ENSG00000204792 | -1.05119 | 6.080514 | -2.77936 | 0.005724 | 0.014289 | -2.79152 | LINC01291 |
| ENSG00000168243 | ENSG00000168243 | -1.05114 | 7.015123 | -3.76313 | 0.000195 | 0.00096 | 0.277836 | GNG4 |
| ENSG00000173432 | ENSG00000173432 | -1.05111 | 8.513262 | -3.30833 | 0.00103 | 0.003629 | -1.24927 | SAA1 |
| ENSG00000259240 | ENSG00000259240 | -1.05103 | 3.624418 | -5.66598 | 2.94E-08 | 9.62E-07 | 8.565404 | MIR4713HG |
| ENSG00000187021 | ENSG00000187021 | -1.05085 | 3.152827 | -4.59863 | 5.84E-06 | 6.09E-05 | 3.550802 | PNLIPRP1 |
| ENSG00000105398 | ENSG00000105398 | -1.05069 | 3.428958 | -3.35195 | 0.000885 | 0.003218 | -1.11078 | SULT2A1 |
| ENSG00000183273 | ENSG00000183273 | -1.04988 | 3.439053 | -4.76182 | 2.76E-06 | 3.36E-05 | 4.258469 | CCDC60 |
| ENSG00000179846 | ENSG00000179846 | -1.04979 | 4.556094 | -6.17002 | 1.79E-09 | 1.10E-07 | 11.23779 | NKPD1 |
| ENSG00000198732 | ENSG00000198732 | -1.04877 | 7.640044 | -3.59922 | 0.000362 | 0.001582 | -0.29362 | SMOC1 |
| ENSG00000019186 | ENSG00000019186 | -1.04852 | 4.206343 | -3.78845 | 0.000177 | 0.00089 | 0.368221 | CYP24A1 |
| ENSG00000089116 | ENSG00000089116 | -1.04832 | 2.220021 | -4.91698 | 1.32E-06 | 1.90E-05 | 4.951515 | LHX5 |
| ENSG00000240990 | ENSG00000240990 | -1.04746 | 6.122394 | -3.46235 | 0.000598 | 0.002355 | -0.75266 | HOXA11-AS |
| ENSG00000189127 | ENSG00000189127 | -1.04716 | 3.107281 | -5.70512 | 2.38E-08 | 8.23E-07 | 8.766168 | ANKRD34B |
| ENSG00000093134 | ENSG00000093134 | -1.04689 | 5.340539 | -4.42436 | 1.27E-05 | 0.000112 | 2.81934 | VNN3 |
| ENSG00000173678 | ENSG00000173678 | -1.04633 | 2.690245 | -6.46541 | 3.19E-10 | 2.93E-08 | 12.88953 | SPDYE2B |
| ENSG00000078898 | ENSG00000078898 | -1.0448 | 2.064457 | -3.64695 | 0.000303 | 0.001372 | -0.12964 | BPIFB2 |
| ENSG00000221275 | ENSG00000221275 | -1.04392 | 2.38982 | -6.74718 | 5.81E-11 | 7.57E-09 | 14.52195 | MIR548I2 |
| ENSG00000198729 | ENSG00000198729 | -1.04371 | 6.179764 | -3.77813 | 0.000184 | 0.000919 | 0.331334 | PPP1R14C |
| ENSG00000187867 | ENSG00000187867 | -1.04311 | 7.696819 | -4.39635 | 1.44E-05 | 0.000123 | 2.704137 | PALM3 |
| ENSG00000114279 | ENSG00000114279 | -1.04286 | 6.301074 | -6.73241 | 6.36E-11 | 8.16E-09 | 14.43503 | FGF12 |
| ENSG00000162931 | ENSG00000162931 | -1.04154 | 6.638826 | -7.62414 | 2.08E-13 | 8.14E-11 | 19.93942 | TRIM17 |
| ENSG00000130294 | ENSG00000130294 | -1.04111 | 5.90155 | -3.11971 | 0.001952 | 0.006015 | -1.82838 | KIF1A |
| ENSG00000144908 | ENSG00000144908 | -1.04049 | 6.235465 | -3.86676 | 0.00013 | 0.000701 | 0.651316 | ALDH1L1 |
| ENSG00000233198 | ENSG00000233198 | -1.04019 | 4.142269 | -5.51502 | 6.55E-08 | 1.83E-06 | 7.801975 | RNF224 |
| ENSG00000100433 | ENSG00000100433 | -1.03938 | 6.700322 | -4.46312 | 1.07E-05 | 9.71E-05 | 2.979826 | KCNK10 |
| ENSG00000213892 | ENSG00000213892 | -1.03837 | 2.899664 | -5.88334 | 8.99E-09 | 3.84E-07 | 9.694837 | CEACAM16 |
| ENSG00000207606 | ENSG00000207606 | -1.03819 | 2.196913 | -7.45007 | 6.61E-13 | 2.09E-10 | 18.82505 | MIR554 |
| ENSG00000157856 | ENSG00000157856 | -1.03769 | 3.41107 | -5.93467 | 6.76E-09 | 3.10E-07 | 9.966689 | DRC1 |
| ENSG00000167419 | ENSG00000167419 | -1.03736 | 3.573587 | -5.38806 | 1.27E-07 | 3.06E-06 | 7.173427 | LPO |
| ENSG00000265933 | ENSG00000265933 | -1.03654 | 6.551248 | -2.70255 | 0.007197 | 0.017206 | -2.99416 | LINC00668 |
| ENSG00000231172 | ENSG00000231172 | -1.03625 | 2.291264 | -4.3136 | 2.06E-05 | 0.000164 | 2.367654 | LOC101927884 |
| ENSG00000197128 | ENSG00000197128 | -1.03604 | 7.889973 | -6.50167 | 2.57E-10 | 2.48E-08 | 13.09653 | ZNF772 |
| ENSG00000126562 | ENSG00000126562 | -1.03588 | 8.27817 | -4.64157 | 4.80E-06 | 5.24E-05 | 3.734895 | WNK4 |
| ENSG00000163915 | ENSG00000163915 | -1.03554 | 4.992011 | -6.2777 | 9.60E-10 | 6.65E-08 | 11.83272 | IGF2BP2-AS1 |
| ENSG00000173947 | ENSG00000173947 | -1.03465 | 6.465408 | -4.39169 | 1.47E-05 | 0.000125 | 2.685044 | PIFO |
| ENSG00000185467 | ENSG00000185467 | -1.03424 | 6.894869 | -5.25912 | 2.45E-07 | 5.09E-06 | 6.547902 | KPNA7 |
| ENSG00000227640 | ENSG00000227640 | -1.03305 | 5.349226 | -2.93079 | 0.00359 | 0.009808 | -2.37607 | SOX21-AS1 |
| ENSG00000228727 | ENSG00000228727 | -1.03304 | 4.780084 | -6.6433 | 1.09E-10 | 1.26E-08 | 13.91376 | SAPCD1 |
| ENSG00000228294 | ENSG00000228294 | -1.0325 | 1.602524 | -6.57005 | 1.70E-10 | 1.78E-08 | 13.48935 | BMS1P17 |
| ENSG00000230725 | ENSG00000230725 | -1.02994 | 1.954992 | -5.06055 | 6.59E-07 | 1.10E-05 | 5.610046 | LOC284798 |
| ENSG00000166359 | ENSG00000166359 | -1.02983 | 5.623638 | -7.89007 | 3.42E-14 | 1.94E-11 | 21.67755 | WDR88 |
| ENSG00000198488 | ENSG00000198488 | -1.02961 | 6.104035 | -2.92778 | 0.003624 | 0.009884 | -2.38454 | B3GNT6 |
| ENSG00000123584 | ENSG00000123584 | -1.02931 | 1.014196 | -4.94377 | 1.16E-06 | 1.72E-05 | 5.073134 | MAGEA9 |
| ENSG00000117601 | ENSG00000117601 | -1.0293 | 2.621416 | -6.54463 | 1.98E-10 | 2.01E-08 | 13.34297 | SERPINC1 |
| ENSG00000223956 | ENSG00000223956 | -1.02885 | 2.949958 | -6.41387 | 4.32E-10 | 3.64E-08 | 12.59687 | LINC01767 |
| ENSG00000234377 | ENSG00000234377 | -1.02866 | 3.200822 | -7.57876 | 2.82E-13 | 1.05E-10 | 19.64713 | OBI1-AS1 |
| ENSG00000168065 | ENSG00000168065 | -1.02785 | 3.423848 | -4.08799 | 5.34E-05 | 0.000346 | 1.479685 | SLC22A11 |
| ENSG00000258932 | ENSG00000258932 | -1.02783 | 1.58542 | -5.23008 | 2.84E-07 | 5.69E-06 | 6.408808 | LOC728755 |
| ENSG00000124092 | ENSG00000124092 | -1.02771 | 3.263952 | -3.5504 | 0.000434 | 0.001822 | -0.45927 | CTCFL |
| ENSG00000241388 | ENSG00000241388 | -1.02704 | 10.0714 | -5.34343 | 1.60E-07 | 3.66E-06 | 6.955445 | HNF1A-AS1 |
| ENSG00000253972 | ENSG00000253972 | -1.02527 | 4.826729 | -7.28408 | 1.96E-12 | 4.94E-10 | 17.77999 | MAL2-AS1 |
| ENSG00000260386 | ENSG00000260386 | -1.02496 | 3.139193 | -4.79038 | 2.41E-06 | 3.02E-05 | 4.384588 | LDC1P |
| ENSG00000069188 | ENSG00000069188 | -1.0249 | 8.002821 | -5.28994 | 2.10E-07 | 4.49E-06 | 6.696239 | SDK2 |
| ENSG00000176945 | ENSG00000176945 | -1.02475 | 10.32673 | -4.35171 | 1.75E-05 | 0.000144 | 2.521927 | MUC20 |
| ENSG00000100604 | ENSG00000100604 | -1.02446 | 7.029827 | -2.58955 | 0.009988 | 0.022417 | -3.28237 | CHGA |
| ENSG00000147889 | ENSG00000147889 | -1.0242 | 9.460504 | -4.10172 | 5.04E-05 | 0.000332 | 1.532507 | CDKN2A |
| ENSG00000125798 | ENSG00000125798 | -1.02336 | 10.48234 | -4.65762 | 4.46E-06 | 4.94E-05 | 3.804096 | FOXA2 |
| ENSG00000183091 | ENSG00000183091 | -1.02267 | 9.111148 | -4.2714 | 2.47E-05 | 0.00019 | 2.198269 | NEB |
| ENSG00000177414 | ENSG00000177414 | -1.02097 | 1.71698 | -5.09356 | 5.60E-07 | 9.76E-06 | 5.763763 | UBE2U |
| ENSG00000253405 | ENSG00000253405 | -1.02045 | 1.725836 | -5.03769 | 7.37E-07 | 1.20E-05 | 5.504089 | EVX1-AS |
| ENSG00000145198 | ENSG00000145198 | -1.02007 | 5.608972 | -4.48588 | 9.70E-06 | 8.97E-05 | 3.074653 | VWA5B2 |
| ENSG00000170615 | ENSG00000170615 | -1.02004 | 4.397779 | -6.06631 | 3.23E-09 | 1.74E-07 | 10.67266 | SLC26A5 |
| ENSG00000124678 | ENSG00000124678 | -1.01988 | 2.935108 | -4.44791 | 1.15E-05 | 0.000103 | 2.916721 | TCP11 |
| ENSG00000088899 | ENSG00000088899 | -1.01958 | 11.16321 | -6.36439 | 5.79E-10 | 4.51E-08 | 12.31768 | LZTS3 |
| ENSG00000106331 | ENSG00000106331 | -1.01953 | 2.319824 | -3.72722 | 0.000224 | 0.001073 | 0.150638 | PAX4 |
| ENSG00000085552 | ENSG00000085552 | -1.01824 | 10.11081 | -6.08636 | 2.88E-09 | 1.59E-07 | 10.78134 | IGSF9 |
| ENSG00000112175 | ENSG00000112175 | -1.01782 | 4.594911 | -4.22103 | 3.06E-05 | 0.000224 | 1.998094 | BMP5 |
| ENSG00000175318 | ENSG00000175318 | -1.01727 | 6.831046 | -5.85524 | 1.05E-08 | 4.29E-07 | 9.546849 | GRAMD2A |
| ENSG00000155622 | ENSG00000155622 | -1.01701 | 1.162313 | -4.89247 | 1.49E-06 | 2.09E-05 | 4.840707 | XAGE2 |
| ENSG00000223658 | ENSG00000223658 | -1.01696 | 4.227562 | -5.19002 | 3.47E-07 | 6.66E-06 | 6.217989 | C1GALT1C1L |
| ENSG00000279516 | ENSG00000279516 | -1.01691 | 1.214165 | -4.2304 | 2.94E-05 | 0.000218 | 2.035173 | FAM230C |
| ENSG00000143595 | ENSG00000143595 | -1.01684 | 2.953713 | -4.41449 | 1.33E-05 | 0.000116 | 2.778683 | AQP10 |
| ENSG00000091513 | ENSG00000091513 | -1.01672 | 5.536245 | -3.4387 | 0.000651 | 0.002516 | -0.83028 | TF |
| ENSG00000214128 | ENSG00000214128 | -1.01651 | 3.605224 | -4.17937 | 3.65E-05 | 0.000257 | 1.834126 | TMEM213 |
| ENSG00000136231 | ENSG00000136231 | -1.01629 | 8.579455 | -3.87436 | 0.000126 | 0.000685 | 0.679063 | IGF2BP3 |
| ENSG00000162738 | ENSG00000162738 | -1.01582 | 9.343797 | -4.9084 | 1.38E-06 | 1.96E-05 | 4.912658 | VANGL2 |
| ENSG00000178965 | ENSG00000178965 | -1.01539 | 3.694971 | -4.03226 | 6.71E-05 | 0.000415 | 1.267052 | ERICH3 |
| ENSG00000101194 | ENSG00000101194 | -1.01474 | 11.12101 | -6.31626 | 7.67E-10 | 5.58E-08 | 12.04779 | SLC17A9 |
| ENSG00000188306 | ENSG00000188306 | -1.01434 | 4.97024 | -5.32981 | 1.71E-07 | 3.86E-06 | 6.889239 | LRRIQ4 |
| ENSG00000230873 | ENSG00000230873 | -1.01425 | 2.26326 | -5.14537 | 4.34E-07 | 7.97E-06 | 6.006845 | STMND1 |
| ENSG00000113327 | ENSG00000113327 | -1.01395 | 2.188434 | -3.99926 | 7.67E-05 | 0.00046 | 1.142362 | GABRG2 |
| ENSG00000255277 | ENSG00000255277 | -1.01347 | 5.121927 | -5.21657 | 3.04E-07 | 6.01E-06 | 6.344314 | ABCC6P2 |
| ENSG00000153822 | ENSG00000153822 | -1.01336 | 4.660751 | -3.74479 | 0.000209 | 0.001017 | 0.212744 | KCNJ16 |
| ENSG00000186897 | ENSG00000186897 | -1.01247 | 2.659362 | -4.8218 | 2.08E-06 | 2.70E-05 | 4.52405 | C1QL4 |
| ENSG00000280953 | ENSG00000280953 | -1.01187 | 1.927913 | -4.8773 | 1.60E-06 | 2.21E-05 | 4.77242 | LINC01163 |
| ENSG00000179242 | ENSG00000179242 | -1.0115 | 5.539827 | -6.43186 | 3.89E-10 | 3.36E-08 | 12.69881 | CDH4 |
| ENSG00000119125 | ENSG00000119125 | -1.01132 | 10.30255 | -4.12166 | 4.64E-05 | 0.000311 | 1.609476 | GDA |
| ENSG00000186148 | ENSG00000186148 | -1.01018 | 1.961425 | -5.43898 | 9.74E-08 | 2.50E-06 | 7.424035 | LOC440895 |
| ENSG00000231221 | ENSG00000231221 | -1.00989 | 1.855814 | -5.52769 | 6.13E-08 | 1.75E-06 | 7.865403 | LINC01593 |
| ENSG00000087510 | ENSG00000087510 | -1.00951 | 7.183345 | -3.96542 | 8.79E-05 | 0.000513 | 1.015526 | TFAP2C |
| ENSG00000254560 | ENSG00000254560 | -1.00924 | 7.061379 | -4.91173 | 1.36E-06 | 1.93E-05 | 4.927717 | BBOX1-AS1 |
| ENSG00000184735 | ENSG00000184735 | -1.009 | 1.646151 | -4.27471 | 2.44E-05 | 0.000188 | 2.211509 | DDX53 |
| ENSG00000165325 | ENSG00000165325 | -1.00662 | 3.050935 | -5.57218 | 4.84E-08 | 1.45E-06 | 8.089029 | DEUP1 |
| ENSG00000174899 | ENSG00000174899 | -1.00636 | 5.566982 | -4.12319 | 4.61E-05 | 0.000309 | 1.615369 | SLC66A1L |
| ENSG00000165643 | ENSG00000165643 | -1.00507 | 1.61503 | -3.99621 | 7.77E-05 | 0.000464 | 1.130891 | SOHLH1 |
| ENSG00000106384 | ENSG00000106384 | -1.00396 | 7.362478 | -3.66905 | 0.000279 | 0.001281 | -0.05303 | MOGAT3 |
| ENSG00000249717 | ENSG00000249717 | -1.00342 | 1.923613 | -6.05907 | 3.36E-09 | 1.79E-07 | 10.63351 | LOC100507388 |
| ENSG00000139865 | ENSG00000139865 | -1.00211 | 6.304727 | -5.35623 | 1.49E-07 | 3.47E-06 | 7.017809 | TTC6 |
| ENSG00000173612 | ENSG00000173612 | -1.00209 | 1.198162 | -5.36593 | 1.42E-07 | 3.34E-06 | 7.065167 | GPRC6A |
| ENSG00000225778 | ENSG00000225778 | -1.00202 | 4.810569 | -6.20728 | 1.44E-09 | 9.26E-08 | 11.4427 | PROSER2-AS1 |
| ENSG00000260704 | ENSG00000260704 | -1.00145 | 7.539247 | -5.50989 | 6.73E-08 | 1.86E-06 | 7.77634 | LINC00543 |
| ENSG00000196482 | ENSG00000196482 | -1.00134 | 5.977052 | -4.13955 | 4.31E-05 | 0.000293 | 1.678794 | ESRRG |
| ENSG00000105929 | ENSG00000105929 | -1.00109 | 3.695376 | -3.68576 | 0.000262 | 0.00122 | 0.005151 | ATP6V0A4 |
| ENSG00000268964 | ENSG00000268964 | -1.00081 | 1.87049 | -4.68257 | 3.98E-06 | 4.50E-05 | 3.912069 | ERVV-2 |
| ENSG00000226747 | ENSG00000226747 | -1.00066 | 2.614435 | -5.13829 | 4.49E-07 | 8.21E-06 | 5.97347 | FSIP2-AS2 |

Table S2. Difflab Cluster B

|  |  |  |  |  |  |  |  |  |
| --- | --- | --- | --- | --- | --- | --- | --- | --- |
|  | ensembl | logFC | AveExpr | t | P.Value | adj.P.Val | B | gene_id |
| ENSG00000121446 | ENSG00000121446 | 1.200305 | 1.942841 | 6.805238 | 4.06E-11 | 1.15E-09 | 14.833 | RGSL1 |
| ENSG00000105143 | ENSG00000105143 | 1.200393 | 2.441179 | 6.343376 | 6.54E-10 | 1.20E-08 | 12.15081 | SLC1A6 |
| ENSG00000204118 | ENSG00000204118 | 1.200919 | 2.30393 | 6.317189 | 7.63E-10 | 1.36E-08 | 12.00323 | NAP1L6P |
| ENSG00000171487 | ENSG00000171487 | 1.201374 | 1.766943 | 5.81016 | 1.34E-08 | 1.55E-07 | 9.243845 | NLRP5 |
| ENSG00000174469 | ENSG00000174469 | 1.201586 | 8.812244 | 4.276754 | 2.42E-05 | 9.21E-05 | 2.110028 | CNTNAP2 |
| ENSG00000254233 | ENSG00000254233 | 1.201864 | 3.543401 | 4.938506 | 1.19E-06 | 6.91E-06 | 4.956425 | LINC02365 |
| ENSG00000226367 | ENSG00000226367 | 1.202224 | 3.094408 | 8.425224 | 8.01E-16 | 1.50E-13 | 25.33696 | ST7-AS2 |
| ENSG00000184368 | ENSG00000184368 | 1.2026 | 5.849467 | 4.149146 | 4.14E-05 | 0.000148 | 1.603471 | MAP7D2 |
| ENSG00000183690 | ENSG00000183690 | 1.203267 | 6.147605 | 6.589324 | 1.52E-10 | 3.50E-09 | 13.56064 | EFHC2 |
| ENSG00000157765 | ENSG00000157765 | 1.203533 | 7.630238 | 3.410037 | 0.000721 | 0.00177 | -1.05185 | SLC34A2 |
| ENSG00000080618 | ENSG00000080618 | 1.203677 | 2.554158 | 6.453043 | 3.43E-10 | 6.95E-09 | 12.77419 | CPB2 |
| ENSG00000123999 | ENSG00000123999 | 1.205708 | 3.827844 | 4.448013 | 1.15E-05 | 4.85E-05 | 2.811621 | INHA |
| ENSG00000099937 | ENSG00000099937 | 1.205862 | 4.851721 | 6.314436 | 7.75E-10 | 1.38E-08 | 11.98774 | SERPIND1 |
| ENSG00000123977 | ENSG00000123977 | 1.206747 | 2.894431 | 6.148424 | 2.02E-09 | 3.09E-08 | 11.06389 | DAW1 |
| ENSG00000279192 | ENSG00000279192 | 1.206897 | 4.964512 | 5.816348 | 1.30E-08 | 1.50E-07 | 9.276373 | PWAR5 |
| ENSG00000189058 | ENSG00000189058 | 1.20728 | 10.96802 | 4.351408 | 1.75E-05 | 6.98E-05 | 2.41281 | APOD |
| ENSG00000130226 | ENSG00000130226 | 1.207502 | 4.700891 | 4.488084 | 9.60E-06 | 4.16E-05 | 2.97935 | DPP6 |
| ENSG00000189431 | ENSG00000189431 | 1.207659 | 6.90572 | 4.077723 | 5.57E-05 | 0.000191 | 1.326044 | RASSF10 |
| ENSG00000213373 | ENSG00000213373 | 1.208313 | 2.578118 | 8.099998 | 8.00E-15 | 9.89E-13 | 23.10062 | LINC00671 |
| ENSG00000224074 | ENSG00000224074 | 1.208337 | 1.461856 | 8.339569 | 1.48E-15 | 2.44E-13 | 24.74216 | LINC00691 |
| ENSG00000236296 | ENSG00000236296 | 1.208357 | 4.046459 | 7.096102 | 6.55E-12 | 2.51E-10 | 16.59694 | GUSBP5 |
| ENSG00000113211 | ENSG00000113211 | 1.20985 | 5.339266 | 5.845673 | 1.11E-08 | 1.31E-07 | 9.430934 | PCDHB6 |
| ENSG00000162843 | ENSG00000162843 | 1.21012 | 2.874297 | 7.143545 | 4.84E-12 | 1.94E-10 | 16.88998 | WDR64 |
| ENSG00000106648 | ENSG00000106648 | 1.210326 | 1.607357 | 6.305148 | 8.18E-10 | 1.44E-08 | 11.93553 | GALNTL5 |
| ENSG00000113805 | ENSG00000113805 | 1.210763 | 4.145181 | 5.541442 | 5.70E-08 | 5.25E-07 | 7.859118 | CNTN3 |
| ENSG00000003989 | ENSG00000003989 | 1.211048 | 9.534332 | 5.254523 | 2.51E-07 | 1.83E-06 | 6.441998 | SLC7A2 |
| ENSG00000232023 | ENSG00000232023 | 1.21213 | 1.443446 | 5.353278 | 1.52E-07 | 1.19E-06 | 6.922504 | LINC01807 |
| ENSG00000145626 | ENSG00000145626 | 1.212151 | 1.543254 | 5.498781 | 7.13E-08 | 6.31E-07 | 7.644358 | UGT3A1 |
| ENSG00000101489 | ENSG00000101489 | 1.21302 | 4.936427 | 5.807879 | 1.36E-08 | 1.56E-07 | 9.23186 | CELF4 |
| ENSG00000115665 | ENSG00000115665 | 1.213089 | 2.783317 | 5.288407 | 2.11E-07 | 1.58E-06 | 6.606 | SLC5A7 |
| ENSG00000258932 | ENSG00000258932 | 1.213428 | 1.58542 | 6.153257 | 1.97E-09 | 3.02E-08 | 11.0905 | LOC728755 |
| ENSG00000136352 | ENSG00000136352 | 1.214723 | 1.77704 | 4.394904 | 1.45E-05 | 5.93E-05 | 2.591397 | NKX2-1 |
| ENSG00000110148 | ENSG00000110148 | 1.215281 | 3.966273 | 4.083341 | 5.44E-05 | 0.000187 | 1.347707 | CCKBR |
| ENSG00000173572 | ENSG00000173572 | 1.21552 | 1.292456 | 6.314424 | 7.75E-10 | 1.38E-08 | 11.98767 | NLRP13 |
| ENSG00000228784 | ENSG00000228784 | 1.215698 | 5.611403 | 6.251252 | 1.12E-09 | 1.87E-08 | 11.63378 | LINC00954 |
| ENSG00000079689 | ENSG00000079689 | 1.21912 | 4.061372 | 3.75869 | 0.000198 | 0.000576 | 0.140853 | SCGN |
| ENSG00000271086 | ENSG00000271086 | 1.21955 | 1.772754 | 8.560158 | 3.03E-16 | 7.14E-14 | 26.28224 | NAMA |
| ENSG00000233485 | ENSG00000233485 | 1.219784 | 2.301322 | 8.477586 | 5.50E-16 | 1.11E-13 | 25.70259 | FHAD1-AS1 |
| ENSG00000258754 | ENSG00000258754 | 1.219992 | 4.905771 | 4.813897 | 2.16E-06 | 1.15E-05 | 4.392775 | LOC105369203 |
| ENSG00000248360 | ENSG00000248360 | 1.221526 | 3.324993 | 6.461373 | 3.26E-10 | 6.67E-09 | 12.82188 | LINC00504 |
| ENSG00000141338 | ENSG00000141338 | 1.22189 | 8.019223 | 5.044409 | 7.13E-07 | 4.46E-06 | 5.445358 | ABCA8 |
| ENSG00000257127 | ENSG00000257127 | 1.222895 | 2.414819 | 7.533272 | 3.81E-13 | 2.39E-11 | 19.35218 | CLLU1 |
| ENSG00000177369 | ENSG00000177369 | 1.223295 | 2.380447 | 7.223339 | 2.90E-12 | 1.27E-10 | 17.38617 | FLJ40194 |
| ENSG00000113073 | ENSG00000113073 | 1.22364 | 3.982154 | 7.945025 | 2.35E-14 | 2.37E-12 | 22.05647 | SLC4A9 |
| ENSG00000113249 | ENSG00000113249 | 1.223773 | 4.867492 | 4.086014 | 5.38E-05 | 0.000186 | 1.358022 | HAVCR1 |
| ENSG00000280953 | ENSG00000280953 | 1.224957 | 1.927913 | 5.887409 | 8.79E-09 | 1.07E-07 | 9.652012 | LINC01163 |
| ENSG00000155754 | ENSG00000155754 | 1.225314 | 4.32958 | 7.218385 | 2.99E-12 | 1.31E-10 | 17.35524 | C2CD6 |
| ENSG00000080293 | ENSG00000080293 | 1.22607 | 5.55799 | 4.715221 | 3.42E-06 | 1.70E-05 | 3.955435 | SCTR |
| ENSG00000176399 | ENSG00000176399 | 1.226584 | 5.139016 | 4.671994 | 4.18E-06 | 2.02E-05 | 3.766375 | DMRTA1 |
| ENSG00000136155 | ENSG00000136155 | 1.227431 | 6.017429 | 3.430616 | 0.00067 | 0.001663 | -0.98446 | SCEL |
| ENSG00000164796 | ENSG00000164796 | 1.227432 | 2.619208 | 5.259734 | 2.44E-07 | 1.79E-06 | 6.467161 | CSMD3 |
| ENSG00000231367 | ENSG00000231367 | 1.227686 | 2.879041 | 6.955781 | 1.59E-11 | 5.23E-10 | 15.73892 | LINC02613 |
| ENSG00000160321 | ENSG00000160321 | 1.2277 | 4.978026 | 7.510869 | 4.42E-13 | 2.69E-11 | 19.20803 | ZNF208 |
| ENSG00000104177 | ENSG00000104177 | 1.227773 | 7.980783 | 5.414737 | 1.10E-07 | 9.11E-07 | 7.2254 | MYEF2 |
| ENSG00000173809 | ENSG00000173809 | 1.228536 | 4.105802 | 5.605152 | 4.07E-08 | 3.96E-07 | 8.182442 | TDRD12 |
| ENSG00000162669 | ENSG00000162669 | 1.228568 | 3.995634 | 6.770344 | 5.04E-11 | 1.38E-09 | 14.62521 | HFM1 |
| ENSG00000197353 | ENSG00000197353 | 1.228677 | 5.108846 | 3.12872 | 0.001895 | 0.004126 | -1.9347 | LYPD2 |
| ENSG00000232229 | ENSG00000232229 | 1.228732 | 6.300137 | 7.189188 | 3.61E-12 | 1.53E-10 | 17.1733 | LINC00865 |
| ENSG00000164744 | ENSG00000164744 | 1.229027 | 2.742384 | 6.39684 | 4.78E-10 | 9.17E-09 | 12.45365 | SUN3 |
| ENSG00000157005 | ENSG00000157005 | 1.22973 | 4.825163 | 3.240977 | 0.001299 | 0.002966 | -1.591 | SST |
| ENSG00000268964 | ENSG00000268964 | 1.229806 | 1.87049 | 5.738709 | 1.98E-08 | 2.16E-07 | 8.870301 | ERVV-2 |
| ENSG00000176601 | ENSG00000176601 | 1.232832 | 2.922668 | 7.312269 | 1.63E-12 | 7.89E-11 | 17.94401 | MAP3K19 |
| ENSG00000006071 | ENSG00000006071 | 1.233293 | 4.441929 | 5.3711 | 1.38E-07 | 1.10E-06 | 7.010036 | ABCC8 |
| ENSG00000165325 | ENSG00000165325 | 1.233803 | 3.050935 | 6.849472 | 3.09E-11 | 9.15E-10 | 15.0976 | DEUP1 |
| ENSG00000185674 | ENSG00000185674 | 1.234354 | 4.216836 | 8.040116 | 1.21E-14 | 1.36E-12 | 22.69549 | LYG2 |
| ENSG00000125888 | ENSG00000125888 | 1.23687 | 1.842002 | 6.040753 | 3.73E-09 | 5.23E-08 | 10.4754 | BANF2 |
| ENSG00000145692 | ENSG00000145692 | 1.238374 | 3.713916 | 5.429678 | 1.02E-07 | 8.53E-07 | 7.299479 | BHMT |
| ENSG00000092850 | ENSG00000092850 | 1.238577 | 3.608659 | 6.750961 | 5.67E-11 | 1.53E-09 | 14.51015 | TEKT2 |
| ENSG00000183760 | ENSG00000183760 | 1.238684 | 3.008896 | 5.477194 | 7.98E-08 | 6.94E-07 | 7.536227 | ACP7 |
| ENSG00000101098 | ENSG00000101098 | 1.238993 | 3.821683 | 4.146866 | 4.18E-05 | 0.000149 | 1.594546 | RIMS4 |
| ENSG00000205038 | ENSG00000205038 | 1.239388 | 5.240866 | 6.34145 | 6.62E-10 | 1.21E-08 | 12.13994 | PKHD1L1 |
| ENSG00000145808 | ENSG00000145808 | 1.239532 | 2.536082 | 5.132505 | 4.62E-07 | 3.09E-06 | 5.858945 | ADAMTS19 |
| ENSG00000248429 | ENSG00000248429 | 1.23967 | 7.173396 | 7.067033 | 7.89E-12 | 2.91E-10 | 16.41812 | FAM198B-AS1 |
| ENSG00000269526 | ENSG00000269526 | 1.24035 | 1.837998 | 6.687406 | 8.37E-11 | 2.13E-09 | 14.13466 | ERVV-1 |
| ENSG00000113327 | ENSG00000113327 | 1.240471 | 2.188434 | 4.859996 | 1.74E-06 | 9.54E-06 | 4.599823 | GABRG2 |
| ENSG00000235597 | ENSG00000235597 | 1.241163 | 1.901498 | 6.423678 | 4.08E-10 | 7.99E-09 | 12.60643 | LINC01102 |
| ENSG00000138823 | ENSG00000138823 | 1.242135 | 6.232311 | 3.608598 | 0.00035 | 0.000944 | -0.38585 | MTTP |
| ENSG00000153002 | ENSG00000153002 | 1.242924 | 3.122361 | 5.228695 | 2.86E-07 | 2.04E-06 | 6.317597 | CPB1 |
| ENSG00000267659 | ENSG00000267659 | 1.242925 | 3.289082 | 9.007062 | 1.13E-17 | 5.55E-15 | 29.48307 | LINC01482 |
| ENSG00000197134 | ENSG00000197134 | 1.243297 | 5.485415 | 6.725905 | 6.62E-11 | 1.74E-09 | 14.36178 | ZNF257 |
| ENSG00000134365 | ENSG00000134365 | 1.244169 | 1.709368 | 6.104083 | 2.61E-09 | 3.85E-08 | 10.82051 | CFHR4 |
| ENSG00000260735 | ENSG00000260735 | 1.244212 | 3.290939 | 9.365529 | 7.48E-19 | 6.40E-16 | 32.12488 | LOC100505915 |
| ENSG00000230666 | ENSG00000230666 | 1.244466 | 3.002229 | 7.626554 | 2.05E-13 | 1.44E-11 | 19.95574 | CEACAM22P |
| ENSG00000232040 | ENSG00000232040 | 1.244493 | 3.99509 | 4.456479 | 1.10E-05 | 4.70E-05 | 2.846947 | ZBED9 |
| ENSG00000040731 | ENSG00000040731 | 1.244666 | 2.43434 | 5.647581 | 3.24E-08 | 3.27E-07 | 8.399492 | CDH10 |
| ENSG00000163395 | ENSG00000163395 | 1.244802 | 4.26617 | 4.81738 | 2.12E-06 | 1.13E-05 | 4.408358 | IGFN1 |
| ENSG00000269067 | ENSG00000269067 | 1.244845 | 2.83931 | 6.414437 | 4.31E-10 | 8.39E-09 | 12.55377 | ZNF728 |
| ENSG00000096088 | ENSG00000096088 | 1.245094 | 12.07228 | 2.480411 | 0.013566 | 0.023227 | -3.69281 | PGC |
| ENSG00000177025 | ENSG00000177025 | 1.245413 | 4.080406 | 6.732219 | 6.37E-11 | 1.68E-09 | 14.39913 | C19orf18 |
| ENSG00000241684 | ENSG00000241684 | 1.245951 | 5.434684 | 6.960404 | 1.55E-11 | 5.12E-10 | 15.76697 | ADAMTS9-AS2 |
| ENSG00000259929 | ENSG00000259929 | 1.245972 | 1.315308 | 7.090987 | 6.77E-12 | 2.58E-10 | 16.56544 | LOC107984893 |
| ENSG00000102243 | ENSG00000102243 | 1.246101 | 4.64112 | 3.514282 | 0.000495 | 0.001278 | -0.70659 | VGLL1 |
| ENSG00000144852 | ENSG00000144852 | 1.246454 | 8.249627 | 3.91396 | 0.000108 | 0.00034 | 0.706588 | NR1I2 |
| ENSG00000251381 | ENSG00000251381 | 1.247205 | 4.06243 | 4.454944 | 1.11E-05 | 4.73E-05 | 2.840537 | LINC00958 |
| ENSG00000281392 | ENSG00000281392 | 1.247357 | 3.650036 | 8.619222 | 1.97E-16 | 5.15E-14 | 26.69915 | LINC00506 |
| ENSG00000101470 | ENSG00000101470 | 1.247442 | 5.656762 | 5.666948 | 2.92E-08 | 2.99E-07 | 8.49902 | TNNC2 |
| ENSG00000237515 | ENSG00000237515 | 1.247499 | 3.649865 | 5.269105 | 2.33E-07 | 1.72E-06 | 6.512465 | SHISA9 |
| ENSG00000173714 | ENSG00000173714 | 1.247696 | 2.677913 | 6.977246 | 1.39E-11 | 4.68E-10 | 15.86932 | WFIKKN2 |
| ENSG00000231605 | ENSG00000231605 | 1.247759 | 1.817207 | 7.140584 | 4.93E-12 | 1.97E-10 | 16.87166 | LINC01363 |
| ENSG00000214652 | ENSG00000214652 | 1.248097 | 3.599967 | 6.074215 | 3.09E-09 | 4.42E-08 | 10.65738 | ZNF727 |
| ENSG00000174527 | ENSG00000174527 | 1.248184 | 4.265126 | 9.476745 | 3.18E-19 | 3.53E-16 | 32.95738 | MYO1H |
| ENSG00000166426 | ENSG00000166426 | 1.248425 | 4.523961 | 3.533665 | 0.000462 | 0.001202 | -0.64132 | CRABP1 |
| ENSG00000006788 | ENSG00000006788 | 1.249016 | 3.578376 | 6.997567 | 1.22E-11 | 4.19E-10 | 15.99306 | MYH13 |
| ENSG00000139973 | ENSG00000139973 | 1.249051 | 3.986342 | 5.587174 | 4.47E-08 | 4.29E-07 | 8.090888 | SYT16 |
| ENSG00000169760 | ENSG00000169760 | 1.249482 | 5.410393 | 5.178002 | 3.68E-07 | 2.54E-06 | 6.074965 | NLGN1 |
| ENSG00000088926 | ENSG00000088926 | 1.24999 | 2.749365 | 5.347414 | 1.56E-07 | 1.22E-06 | 6.893762 | F11 |
| ENSG00000231683 | ENSG00000231683 | 1.250162 | 2.229123 | 5.271969 | 2.30E-07 | 1.70E-06 | 6.526329 | LOC101927136 |
| ENSG00000139910 | ENSG00000139910 | 1.250367 | 6.954273 | 6.003481 | 4.60E-09 | 6.24E-08 | 10.27367 | NOVA1 |
| ENSG00000127325 | ENSG00000127325 | 1.250831 | 3.189228 | 5.521054 | 6.34E-08 | 5.72E-07 | 7.756308 | BEST3 |
| ENSG00000223882 | ENSG00000223882 | 1.250908 | 2.119773 | 8.816373 | 4.65E-17 | 1.67E-14 | 28.10439 | ABCC5-AS1 |
| ENSG00000130876 | ENSG00000130876 | 1.251172 | 2.939164 | 4.385637 | 1.51E-05 | 6.15E-05 | 2.553212 | SLC7A10 |
| ENSG00000198883 | ENSG00000198883 | 1.251557 | 3.016168 | 4.234399 | 2.89E-05 | 0.000108 | 1.940352 | PNMA5 |
| ENSG00000047936 | ENSG00000047936 | 1.251808 | 4.65671 | 4.651916 | 4.58E-06 | 2.19E-05 | 3.679087 | ROS1 |
| ENSG00000153993 | ENSG00000153993 | 1.253464 | 6.652087 | 5.585516 | 4.51E-08 | 4.32E-07 | 8.082458 | SEMA3D |
| ENSG00000171126 | ENSG00000171126 | 1.254098 | 4.030934 | 5.778647 | 1.60E-08 | 1.79E-07 | 9.07862 | KCNG3 |
| ENSG00000226891 | ENSG00000226891 | 1.254692 | 4.915466 | 8.439257 | 7.24E-16 | 1.37E-13 | 25.4348 | LINC01359 |
| ENSG00000162992 | ENSG00000162992 | 1.255034 | 3.183167 | 4.648466 | 4.65E-06 | 2.22E-05 | 3.664119 | NEUROD1 |
| ENSG00000148123 | ENSG00000148123 | 1.255187 | 3.891964 | 5.038614 | 7.34E-07 | 4.57E-06 | 5.41837 | PLPPR1 |
| ENSG00000135374 | ENSG00000135374 | 1.256035 | 4.255047 | 3.952707 | 9.25E-05 | 0.000297 | 0.851051 | ELF5 |
| ENSG00000139304 | ENSG00000139304 | 1.257574 | 2.876834 | 6.455863 | 3.37E-10 | 6.85E-09 | 12.79033 | PTPRQ |
| ENSG00000205212 | ENSG00000205212 | 1.258329 | 2.332064 | 5.104209 | 5.32E-07 | 3.48E-06 | 5.725425 | CCDC144NL |
| ENSG00000152592 | ENSG00000152592 | 1.258736 | 2.727135 | 6.210605 | 1.42E-09 | 2.30E-08 | 11.4076 | DMP1 |
| ENSG00000129295 | ENSG00000129295 | 1.25907 | 6.980852 | 7.879741 | 3.68E-14 | 3.44E-12 | 21.62085 | LRRC6 |
| ENSG00000116183 | ENSG00000116183 | 1.259119 | 4.740656 | 5.400352 | 1.19E-07 | 9.68E-07 | 7.154243 | PAPPA2 |
| ENSG00000082556 | ENSG00000082556 | 1.259779 | 2.473939 | 5.606616 | 4.03E-08 | 3.94E-07 | 8.189908 | OPRK1 |
| ENSG00000264424 | ENSG00000264424 | 1.259926 | 2.362171 | 5.472538 | 8.18E-08 | 7.08E-07 | 7.512951 | MYH4 |
| ENSG00000183844 | ENSG00000183844 | 1.2609 | 9.213855 | 4.140255 | 4.30E-05 | 0.000152 | 1.568697 | FAM3B |
| ENSG00000204296 | ENSG00000204296 | 1.262157 | 1.86163 | 7.260832 | 2.28E-12 | 1.04E-10 | 17.62073 | TSBP1 |
| ENSG00000171560 | ENSG00000171560 | 1.262193 | 4.646239 | 3.308329 | 0.00103 | 0.002419 | -1.3793 | FGA |
| ENSG00000258525 | ENSG00000258525 | 1.26231 | 3.151163 | 7.779355 | 7.29E-14 | 6.16E-12 | 20.956 | LOC100506071 |
| ENSG00000184226 | ENSG00000184226 | 1.26277 | 6.776634 | 5.562137 | 5.11E-08 | 4.80E-07 | 7.963803 | PCDH9 |
| ENSG00000224271 | ENSG00000224271 | 1.263642 | 3.383156 | 4.045166 | 6.36E-05 | 0.000215 | 1.201043 | LOC284930 |
| ENSG00000185247 | ENSG00000185247 | 1.263699 | 2.530595 | 3.654631 | 0.000295 | 0.000813 | -0.22643 | MAGEA11 |
| ENSG00000183850 | ENSG00000183850 | 1.263857 | 3.445025 | 7.049846 | 8.79E-12 | 3.18E-10 | 16.31266 | ZNF730 |
| ENSG00000187527 | ENSG00000187527 | 1.265085 | 2.313248 | 6.866709 | 2.78E-11 | 8.33E-10 | 15.20107 | ATP13A5 |
| ENSG00000228340 | ENSG00000228340 | 1.265727 | 3.902726 | 6.897129 | 2.30E-11 | 7.09E-10 | 15.38415 | MIR646HG |
| ENSG00000257842 | ENSG00000257842 | 1.265824 | 2.049474 | 4.896907 | 1.46E-06 | 8.20E-06 | 4.766851 | LINC02588 |
| ENSG00000171121 | ENSG00000171121 | 1.265866 | 6.515772 | 8.698302 | 1.11E-16 | 3.27E-14 | 27.26031 | KCNMB3 |
| ENSG00000280543 | ENSG00000280543 | 1.26587 | 4.902035 | 9.98951 | 5.73E-21 | 1.43E-17 | 36.87056 | ASAP1-IT2 |
| ENSG00000124479 | ENSG00000124479 | 1.26663 | 4.052892 | 6.107913 | 2.55E-09 | 3.77E-08 | 10.84148 | NDP |
| ENSG00000135917 | ENSG00000135917 | 1.268117 | 8.248015 | 4.384664 | 1.51E-05 | 6.17E-05 | 2.54921 | SLC19A3 |
| ENSG00000204140 | ENSG00000204140 | 1.268606 | 2.464993 | 5.39602 | 1.22E-07 | 9.87E-07 | 7.132842 | CLPSL1 |
| ENSG00000267978 | ENSG00000267978 | 1.268677 | 1.772308 | 4.167113 | 3.84E-05 | 0.000138 | 1.67395 | MAGEA9B |
| ENSG00000071991 | ENSG00000071991 | 1.26873 | 5.302938 | 4.281118 | 2.37E-05 | 9.06E-05 | 2.127597 | CDH19 |
| ENSG00000144285 | ENSG00000144285 | 1.26874 | 2.120638 | 7.013464 | 1.11E-11 | 3.86E-10 | 16.09005 | SCN1A |
| ENSG00000118156 | ENSG00000118156 | 1.268798 | 4.505373 | 6.38258 | 5.20E-10 | 9.85E-09 | 12.37268 | ZNF541 |
| ENSG00000224468 | ENSG00000224468 | 1.269288 | 3.471367 | 8.915725 | 2.23E-17 | 9.63E-15 | 28.82034 | LAMC1-AS1 |
| ENSG00000255794 | ENSG00000255794 | 1.26947 | 2.739675 | 6.172065 | 1.77E-09 | 2.76E-08 | 11.19424 | RMST |
| ENSG00000165300 | ENSG00000165300 | 1.269814 | 5.473641 | 4.703402 | 3.61E-06 | 1.79E-05 | 3.90359 | SLITRK5 |
| ENSG00000174226 | ENSG00000174226 | 1.270195 | 3.359979 | 6.897541 | 2.29E-11 | 7.07E-10 | 15.38664 | SNX31 |
| ENSG00000181690 | ENSG00000181690 | 1.270408 | 7.70538 | 7.653514 | 1.71E-13 | 1.24E-11 | 20.13119 | PLAG1 |
| ENSG00000147724 | ENSG00000147724 | 1.271719 | 3.46983 | 5.625362 | 3.65E-08 | 3.62E-07 | 8.285657 | FAM135B |
| ENSG00000106809 | ENSG00000106809 | 1.272084 | 8.984186 | 4.2014 | 3.33E-05 | 0.000122 | 1.809214 | OGN |
| ENSG00000231419 | ENSG00000231419 | 1.272217 | 3.534897 | 5.934245 | 6.78E-09 | 8.64E-08 | 9.90165 | LINC00689 |
| ENSG00000100652 | ENSG00000100652 | 1.272493 | 3.217018 | 9.442108 | 4.15E-19 | 4.12E-16 | 32.69747 | SLC10A1 |
| ENSG00000101349 | ENSG00000101349 | 1.272515 | 2.801421 | 5.381527 | 1.31E-07 | 1.05E-06 | 7.061361 | PAK5 |
| ENSG00000046774 | ENSG00000046774 | 1.274261 | 1.857558 | 3.643751 | 0.000307 | 0.000842 | -0.26428 | MAGEC2 |
| ENSG00000228262 | ENSG00000228262 | 1.275405 | 1.706832 | 7.12794 | 5.35E-12 | 2.11E-10 | 16.79344 | LINC01317 |
| ENSG00000250682 | ENSG00000250682 | 1.275423 | 1.742216 | 5.418205 | 1.09E-07 | 8.97E-07 | 7.24258 | LINC00491 |
| ENSG00000134873 | ENSG00000134873 | 1.276876 | 3.811931 | 4.358599 | 1.70E-05 | 6.79E-05 | 2.442222 | CLDN10 |
| ENSG00000188580 | ENSG00000188580 | 1.277033 | 3.960719 | 4.628168 | 5.11E-06 | 2.40E-05 | 3.576272 | NKAIN2 |
| ENSG00000229314 | ENSG00000229314 | 1.277459 | 4.476661 | 3.429274 | 0.000673 | 0.00167 | -0.98887 | ORM1 |
| ENSG00000163833 | ENSG00000163833 | 1.277545 | 2.072991 | 7.970504 | 1.97E-14 | 2.05E-12 | 22.22716 | FBXO40 |
| ENSG00000242512 | ENSG00000242512 | 1.277619 | 2.28483 | 6.145675 | 2.06E-09 | 3.13E-08 | 11.04876 | LINC01206 |
| ENSG00000235097 | ENSG00000235097 | 1.278012 | 2.254879 | 6.268809 | 1.01E-09 | 1.72E-08 | 11.73185 | LINC00330 |
| ENSG00000150676 | ENSG00000150676 | 1.278646 | 2.13178 | 8.103245 | 7.82E-15 | 9.69E-13 | 23.12265 | CCDC83 |
| ENSG00000113209 | ENSG00000113209 | 1.280099 | 6.401248 | 6.17178 | 1.77E-09 | 2.76E-08 | 11.19266 | PCDHB5 |
| ENSG00000107518 | ENSG00000107518 | 1.280586 | 5.386862 | 6.378062 | 5.34E-10 | 1.01E-08 | 12.34705 | ATRNL1 |
| ENSG00000105398 | ENSG00000105398 | 1.281272 | 3.428958 | 4.047774 | 6.30E-05 | 0.000213 | 1.21102 | SULT2A1 |
| ENSG00000273111 | ENSG00000273111 | 1.281562 | 1.99874 | 6.700317 | 7.74E-11 | 1.99E-09 | 14.21071 | LYPD4 |
| ENSG00000163817 | ENSG00000163817 | 1.281707 | 9.689994 | 4.538223 | 7.67E-06 | 3.42E-05 | 3.19112 | SLC6A20 |
| ENSG00000256340 | ENSG00000256340 | 1.284144 | 6.00017 | 5.652967 | 3.15E-08 | 3.19E-07 | 8.427141 | ABCC6P1 |
| ENSG00000089225 | ENSG00000089225 | 1.285787 | 3.194695 | 4.778042 | 2.55E-06 | 1.33E-05 | 4.232936 | TBX5 |
| ENSG00000019186 | ENSG00000019186 | 1.285848 | 4.206343 | 4.61084 | 5.53E-06 | 2.58E-05 | 3.501549 | CYP24A1 |
| ENSG00000227733 | ENSG00000227733 | 1.287431 | 3.730809 | 7.872857 | 3.85E-14 | 3.58E-12 | 21.57507 | LOC101927468 |
| ENSG00000113889 | ENSG00000113889 | 1.288566 | 3.365505 | 5.910105 | 7.75E-09 | 9.66E-08 | 9.772779 | KNG1 |
| ENSG00000173258 | ENSG00000173258 | 1.288641 | 6.256724 | 8.692372 | 1.16E-16 | 3.34E-14 | 27.21812 | ZNF483 |
| ENSG00000140093 | ENSG00000140093 | 1.288682 | 3.718337 | 4.206047 | 3.26E-05 | 0.00012 | 1.827626 | SERPINA10 |
| ENSG00000105929 | ENSG00000105929 | 1.28906 | 3.695376 | 4.720999 | 3.33E-06 | 1.67E-05 | 3.980823 | ATP6V0A4 |
| ENSG00000152977 | ENSG00000152977 | 1.289317 | 2.434582 | 4.549444 | 7.30E-06 | 3.28E-05 | 3.238802 | ZIC1 |
| ENSG00000013293 | ENSG00000013293 | 1.289871 | 3.151362 | 5.720914 | 2.18E-08 | 2.34E-07 | 8.777867 | SLC7A14 |
| ENSG00000229676 | ENSG00000229676 | 1.290573 | 3.355557 | 6.67461 | 9.05E-11 | 2.27E-09 | 14.0594 | ZNF492 |
| ENSG00000132554 | ENSG00000132554 | 1.291055 | 4.073227 | 7.1902 | 3.59E-12 | 1.53E-10 | 17.17959 | RGS22 |
| ENSG00000184647 | ENSG00000184647 | 1.292147 | 2.2387 | 6.844566 | 3.18E-11 | 9.37E-10 | 15.06819 | PRSS55 |
| ENSG00000152936 | ENSG00000152936 | 1.292385 | 1.695181 | 6.938176 | 1.78E-11 | 5.74E-10 | 15.63218 | LMNTD1 |
| ENSG00000236039 | ENSG00000236039 | 1.294221 | 4.723743 | 5.346436 | 1.57E-07 | 1.22E-06 | 6.888966 | LOC101927630 |
| ENSG00000108231 | ENSG00000108231 | 1.29428 | 3.106696 | 5.980614 | 5.23E-09 | 6.94E-08 | 10.15041 | LGI1 |
| ENSG00000117501 | ENSG00000117501 | 1.295818 | 2.5335 | 7.628062 | 2.02E-13 | 1.43E-11 | 19.96554 | MROH9 |
| ENSG00000226807 | ENSG00000226807 | 1.296229 | 2.296537 | 6.933369 | 1.83E-11 | 5.89E-10 | 15.60308 | MROH5 |
| ENSG00000138347 | ENSG00000138347 | 1.296429 | 4.179759 | 6.448514 | 3.52E-10 | 7.10E-09 | 12.74827 | MYPN |
| ENSG00000203730 | ENSG00000203730 | 1.296445 | 2.468374 | 8.478958 | 5.45E-16 | 1.10E-13 | 25.71219 | TEDDM1 |
| ENSG00000235824 | ENSG00000235824 | 1.297064 | 1.928902 | 7.303241 | 1.73E-12 | 8.29E-11 | 17.88715 | LINC00837 |
| ENSG00000171557 | ENSG00000171557 | 1.297418 | 2.872539 | 3.624 | 0.000331 | 0.000898 | -0.33272 | FGG |
| ENSG00000224310 | ENSG00000224310 | 1.298032 | 1.88547 | 7.635866 | 1.92E-13 | 1.37E-11 | 20.0163 | LINC01567 |
| ENSG00000144229 | ENSG00000144229 | 1.298032 | 5.20336 | 5.244323 | 2.64E-07 | 1.91E-06 | 6.392809 | THSD7B |
| ENSG00000016490 | ENSG00000016490 | 1.299085 | 4.417145 | 3.154093 | 0.001741 | 0.003828 | -1.85802 | CLCA1 |
| ENSG00000213973 | ENSG00000213973 | 1.299635 | 3.140941 | 7.493807 | 4.95E-13 | 2.97E-11 | 19.09846 | ZNF99 |
| ENSG00000164591 | ENSG00000164591 | 1.299835 | 5.169145 | 7.01302 | 1.11E-11 | 3.87E-10 | 16.08734 | MYOZ3 |
| ENSG00000251629 | ENSG00000251629 | 1.30027 | 1.697781 | 5.428045 | 1.03E-07 | 8.59E-07 | 7.291373 | LINC02241 |
| ENSG00000104941 | ENSG00000104941 | 1.300481 | 2.08922 | 7.587692 | 2.65E-13 | 1.79E-11 | 19.70364 | RSPH6A |
| ENSG00000091513 | ENSG00000091513 | 1.300573 | 5.536245 | 4.367689 | 1.63E-05 | 6.57E-05 | 2.47947 | TF |
| ENSG00000177414 | ENSG00000177414 | 1.300751 | 1.71698 | 6.514676 | 2.37E-10 | 5.11E-09 | 13.12825 | UBE2U |
| ENSG00000180875 | ENSG00000180875 | 1.300906 | 7.416711 | 4.459019 | 1.09E-05 | 4.65E-05 | 2.857554 | GREM2 |
| ENSG00000124194 | ENSG00000124194 | 1.301992 | 3.866152 | 7.594435 | 2.54E-13 | 1.72E-11 | 19.74731 | GDAP1L1 |
| ENSG00000118492 | ENSG00000118492 | 1.303086 | 1.878537 | 7.08338 | 7.11E-12 | 2.68E-10 | 16.51862 | ADGB |
| ENSG00000165078 | ENSG00000165078 | 1.303975 | 4.078214 | 5.21466 | 3.07E-07 | 2.17E-06 | 6.250219 | CPA6 |
| ENSG00000030304 | ENSG00000030304 | 1.304026 | 5.051919 | 5.613539 | 3.89E-08 | 3.81E-07 | 8.225235 | MUSK |
| ENSG00000164007 | ENSG00000164007 | 1.304956 | 3.250543 | 5.845192 | 1.11E-08 | 1.31E-07 | 9.42839 | CLDN19 |
| ENSG00000175267 | ENSG00000175267 | 1.305467 | 3.671572 | 7.400506 | 9.16E-13 | 4.93E-11 | 18.50252 | VWA3A |
| ENSG00000072657 | ENSG00000072657 | 1.305571 | 6.321737 | 5.308975 | 1.90E-07 | 1.44E-06 | 6.705996 | TRHDE |
| ENSG00000185038 | ENSG00000185038 | 1.305771 | 3.035871 | 5.539892 | 5.74E-08 | 5.28E-07 | 7.851289 | MROH2A |
| ENSG00000141485 | ENSG00000141485 | 1.305782 | 4.687623 | 5.365097 | 1.43E-07 | 1.13E-06 | 6.980527 | SLC13A5 |
| ENSG00000261863 | ENSG00000261863 | 1.307731 | 3.428913 | 4.816876 | 2.13E-06 | 1.13E-05 | 4.406103 | LINC01996 |
| ENSG00000179869 | ENSG00000179869 | 1.307999 | 6.769763 | 5.473366 | 8.14E-08 | 7.06E-07 | 7.517089 | ABCA13 |
| ENSG00000021461 | ENSG00000021461 | 1.309234 | 2.372421 | 8.840463 | 3.89E-17 | 1.48E-14 | 28.27751 | CYP3A43 |
| ENSG00000249961 | ENSG00000249961 | 1.309738 | 2.707792 | 7.935513 | 2.51E-14 | 2.49E-12 | 21.99284 | TERB1 |
| ENSG00000136546 | ENSG00000136546 | 1.31034 | 6.00814 | 4.644926 | 4.73E-06 | 2.25E-05 | 3.648773 | SCN7A |
| ENSG00000129151 | ENSG00000129151 | 1.312757 | 4.351736 | 6.584648 | 1.56E-10 | 3.59E-09 | 13.53344 | BBOX1 |
| ENSG00000234224 | ENSG00000234224 | 1.313585 | 4.148172 | 4.630539 | 5.05E-06 | 2.38E-05 | 3.586516 | TMEM229A |
| ENSG00000178568 | ENSG00000178568 | 1.313835 | 3.850611 | 6.01831 | 4.23E-09 | 5.82E-08 | 10.35381 | ERBB4 |
| ENSG00000184571 | ENSG00000184571 | 1.313983 | 2.2454 | 6.354388 | 6.14E-10 | 1.13E-08 | 12.21302 | PIWIL3 |
| ENSG00000185352 | ENSG00000185352 | 1.314953 | 4.450676 | 4.925744 | 1.27E-06 | 7.29E-06 | 4.898119 | HS6ST3 |
| ENSG00000113492 | ENSG00000113492 | 1.315812 | 1.918174 | 6.608488 | 1.35E-10 | 3.18E-09 | 13.67227 | AGXT2 |
| ENSG00000070729 | ENSG00000070729 | 1.317809 | 3.939464 | 7.374879 | 1.08E-12 | 5.65E-11 | 18.3398 | CNGB1 |
| ENSG00000105392 | ENSG00000105392 | 1.318076 | 3.22498 | 6.968539 | 1.47E-11 | 4.89E-10 | 15.81638 | CRX |
| ENSG00000188883 | ENSG00000188883 | 1.319224 | 4.026301 | 4.851202 | 1.81E-06 | 9.87E-06 | 4.560191 | KLRG2 |
| ENSG00000112796 | ENSG00000112796 | 1.319359 | 7.591582 | 5.962322 | 5.79E-09 | 7.55E-08 | 10.05208 | ENPP5 |
| ENSG00000174898 | ENSG00000174898 | 1.319588 | 2.630083 | 6.673262 | 9.12E-11 | 2.28E-09 | 14.05147 | CATSPERD |
| ENSG00000124939 | ENSG00000124939 | 1.320143 | 3.834484 | 4.406948 | 1.37E-05 | 5.67E-05 | 2.641129 | SCGB2A1 |
| ENSG00000164089 | ENSG00000164089 | 1.321317 | 2.549216 | 5.754159 | 1.82E-08 | 2.01E-07 | 8.950746 | ETNPPL |
| ENSG00000110318 | ENSG00000110318 | 1.321748 | 8.187217 | 9.224214 | 2.20E-18 | 1.46E-15 | 31.07577 | CEP126 |
| ENSG00000172915 | ENSG00000172915 | 1.321822 | 8.393507 | 6.193737 | 1.56E-09 | 2.49E-08 | 11.31408 | NBEA |
| ENSG00000179520 | ENSG00000179520 | 1.322109 | 2.27892 | 5.762562 | 1.74E-08 | 1.93E-07 | 8.994576 | SLC17A8 |
| ENSG00000168702 | ENSG00000168702 | 1.324024 | 3.794827 | 6.70832 | 7.37E-11 | 1.91E-09 | 14.25791 | LRP1B |
| ENSG00000215838 | ENSG00000215838 | 1.325145 | 2.388315 | 7.678177 | 1.45E-13 | 1.07E-11 | 20.29207 | LOC440700 |
| ENSG00000239649 | ENSG00000239649 | 1.325821 | 2.56108 | 7.467988 | 5.87E-13 | 3.42E-11 | 18.933 | MYADML |
| ENSG00000187416 | ENSG00000187416 | 1.325845 | 4.69065 | 4.816778 | 2.13E-06 | 1.14E-05 | 4.405661 | LHFPL3 |
| ENSG00000233008 | ENSG00000233008 | 1.326363 | 3.422422 | 7.709976 | 1.17E-13 | 9.03E-12 | 20.50006 | LOC101927560 |
| ENSG00000096395 | ENSG00000096395 | 1.326401 | 1.94193 | 5.029021 | 7.69E-07 | 4.76E-06 | 5.373753 | MLN |
| ENSG00000077935 | ENSG00000077935 | 1.326784 | 5.256215 | 6.582556 | 1.58E-10 | 3.62E-09 | 13.52128 | SMC1B |
| ENSG00000183960 | ENSG00000183960 | 1.327297 | 6.742912 | 5.371115 | 1.38E-07 | 1.10E-06 | 7.010111 | KCNH8 |
| ENSG00000165181 | ENSG00000165181 | 1.329158 | 5.784442 | 9.238677 | 1.97E-18 | 1.36E-15 | 31.18269 | SHOC1 |
| ENSG00000196090 | ENSG00000196090 | 1.329719 | 5.269812 | 5.812012 | 1.33E-08 | 1.53E-07 | 9.253578 | PTPRT |
| ENSG00000205500 | ENSG00000205500 | 1.329727 | 3.117768 | 9.400959 | 5.70E-19 | 5.20E-16 | 32.38944 | MAPRE3-AS1 |
| ENSG00000170615 | ENSG00000170615 | 1.33032 | 4.397779 | 8.044254 | 1.18E-14 | 1.33E-12 | 22.72341 | SLC26A5 |
| ENSG00000198547 | ENSG00000198547 | 1.330974 | 3.090952 | 7.414336 | 8.36E-13 | 4.57E-11 | 18.59051 | C20orf203 |
| ENSG00000186094 | ENSG00000186094 | 1.331186 | 3.427837 | 6.404429 | 4.57E-10 | 8.83E-09 | 12.4968 | AGBL4 |
| ENSG00000168631 | ENSG00000168631 | 1.331208 | 10.1245 | 3.03869 | 0.002544 | 0.00534 | -2.20204 | MUCL3 |
| ENSG00000140279 | ENSG00000140279 | 1.33172 | 10.99716 | 3.701377 | 0.000247 | 0.000697 | -0.06262 | DUOX2 |
| ENSG00000132972 | ENSG00000132972 | 1.331762 | 2.00459 | 6.492629 | 2.71E-10 | 5.70E-09 | 13.00129 | RNF17 |
| ENSG00000197753 | ENSG00000197753 | 1.331973 | 3.373191 | 7.193851 | 3.51E-12 | 1.50E-10 | 17.20232 | LHFPL5 |
| ENSG00000130612 | ENSG00000130612 | 1.333936 | 2.415594 | 8.704229 | 1.06E-16 | 3.23E-14 | 27.30251 | CYP2G1P |
| ENSG00000138083 | ENSG00000138083 | 1.33692 | 4.034702 | 4.65311 | 4.56E-06 | 2.18E-05 | 3.684268 | SIX3 |
| ENSG00000197410 | ENSG00000197410 | 1.337929 | 6.462057 | 5.234164 | 2.78E-07 | 1.99E-06 | 6.343895 | DCHS2 |
| ENSG00000132958 | ENSG00000132958 | 1.337945 | 3.12413 | 8.147212 | 5.75E-15 | 7.38E-13 | 23.42152 | TPTE2 |
| ENSG00000113396 | ENSG00000113396 | 1.337969 | 3.458433 | 6.096542 | 2.72E-09 | 3.98E-08 | 10.77927 | SLC27A6 |
| ENSG00000143921 | ENSG00000143921 | 1.339102 | 4.912143 | 4.637174 | 4.90E-06 | 2.32E-05 | 3.615207 | ABCG8 |
| ENSG00000187151 | ENSG00000187151 | 1.339353 | 2.124343 | 8.323542 | 1.66E-15 | 2.65E-13 | 24.63132 | ANGPTL5 |
| ENSG00000107984 | ENSG00000107984 | 1.339382 | 7.367135 | 3.430202 | 0.000671 | 0.001665 | -0.98582 | DKK1 |
| ENSG00000273777 | ENSG00000273777 | 1.34058 | 3.402821 | 5.155802 | 4.12E-07 | 2.80E-06 | 5.969352 | CEACAM20 |
| ENSG00000206531 | ENSG00000206531 | 1.341153 | 1.87909 | 7.629018 | 2.01E-13 | 1.42E-11 | 19.97176 | CD200R1L |
| ENSG00000152527 | ENSG00000152527 | 1.342367 | 9.235346 | 8.561629 | 3.00E-16 | 7.13E-14 | 26.2926 | PLEKHH2 |
| ENSG00000234693 | ENSG00000234693 | 1.343661 | 2.409928 | 8.623987 | 1.91E-16 | 5.02E-14 | 26.73287 | LOC100506175 |
| ENSG00000216588 | ENSG00000216588 | 1.343826 | 4.216063 | 5.758438 | 1.78E-08 | 1.97E-07 | 8.973059 | IGSF23 |
| ENSG00000137875 | ENSG00000137875 | 1.344368 | 5.125952 | 5.380257 | 1.32E-07 | 1.06E-06 | 7.055108 | BCL2L10 |
| ENSG00000005981 | ENSG00000005981 | 1.344543 | 4.056646 | 3.838711 | 0.000145 | 0.00044 | 0.429777 | ASB4 |
| ENSG00000140274 | ENSG00000140274 | 1.345455 | 7.991593 | 3.567984 | 0.000407 | 0.001078 | -0.52493 | DUOXA2 |
| ENSG00000099960 | ENSG00000099960 | 1.346233 | 6.189957 | 4.768844 | 2.67E-06 | 1.37E-05 | 4.192106 | SLC7A4 |
| ENSG00000090402 | ENSG00000090402 | 1.346417 | 7.858456 | 3.041597 | 0.002521 | 0.005296 | -2.19352 | SI |
| ENSG00000182230 | ENSG00000182230 | 1.347597 | 3.407103 | 7.092982 | 6.69E-12 | 2.55E-10 | 16.57773 | FAM153B |
| ENSG00000169550 | ENSG00000169550 | 1.348081 | 2.807086 | 4.092611 | 5.24E-05 | 0.000181 | 1.38351 | MUC15 |
| ENSG00000197816 | ENSG00000197816 | 1.348321 | 4.891437 | 8.226812 | 3.29E-15 | 4.56E-13 | 23.96546 | CCDC180 |
| ENSG00000152578 | ENSG00000152578 | 1.349264 | 3.921132 | 7.548392 | 3.45E-13 | 2.20E-11 | 19.44964 | GRIA4 |
| ENSG00000167759 | ENSG00000167759 | 1.349793 | 5.40485 | 4.183746 | 3.58E-05 | 0.00013 | 1.739442 | KLK13 |
| ENSG00000204655 | ENSG00000204655 | 1.350424 | 2.009089 | 7.198362 | 3.41E-12 | 1.46E-10 | 17.23041 | MOG |
| ENSG00000165899 | ENSG00000165899 | 1.350547 | 4.513536 | 7.617933 | 2.17E-13 | 1.51E-11 | 19.89974 | OTOGL |
| ENSG00000236279 | ENSG00000236279 | 1.353109 | 4.320681 | 5.073287 | 6.19E-07 | 3.96E-06 | 5.580244 | CLEC2L |
| ENSG00000183166 | ENSG00000183166 | 1.353208 | 2.730686 | 5.970936 | 5.52E-09 | 7.26E-08 | 10.09836 | CALN1 |
| ENSG00000272808 | ENSG00000272808 | 1.353598 | 3.484567 | 6.952515 | 1.62E-11 | 5.32E-10 | 15.7191 | LOC105369201 |
| ENSG00000182111 | ENSG00000182111 | 1.356844 | 2.227078 | 5.484775 | 7.67E-08 | 6.72E-07 | 7.574162 | ZNF716 |
| ENSG00000150275 | ENSG00000150275 | 1.357308 | 2.581667 | 6.756377 | 5.49E-11 | 1.49E-09 | 14.54227 | PCDH15 |
| ENSG00000225868 | ENSG00000225868 | 1.357752 | 2.77227 | 6.971467 | 1.44E-11 | 4.82E-10 | 15.83418 | LOC100631378 |
| ENSG00000183145 | ENSG00000183145 | 1.35917 | 4.713621 | 4.885991 | 1.53E-06 | 8.57E-06 | 4.717341 | RIPPLY3 |
| ENSG00000225329 | ENSG00000225329 | 1.359227 | 6.757425 | 4.906907 | 1.39E-06 | 7.88E-06 | 4.812297 | LHFPL3-AS2 |
| ENSG00000171804 | ENSG00000171804 | 1.359349 | 3.009247 | 6.729997 | 6.45E-11 | 1.70E-09 | 14.38598 | WDR87 |
| ENSG00000072041 | ENSG00000072041 | 1.359384 | 2.900701 | 5.401144 | 1.19E-07 | 9.65E-07 | 7.158155 | SLC6A15 |
| ENSG00000163515 | ENSG00000163515 | 1.359474 | 3.412988 | 5.07537 | 6.13E-07 | 3.92E-06 | 5.590004 | RETNLB |
| ENSG00000259417 | ENSG00000259417 | 1.361436 | 4.333013 | 5.740212 | 1.97E-08 | 2.14E-07 | 8.878118 | CTXND1 |
| ENSG00000137948 | ENSG00000137948 | 1.361706 | 2.864586 | 6.582567 | 1.58E-10 | 3.62E-09 | 13.52134 | BRDT |
| ENSG00000179447 | ENSG00000179447 | 1.36176 | 2.868936 | 8.843864 | 3.80E-17 | 1.45E-14 | 28.30197 | LOC100130264 |
| ENSG00000121207 | ENSG00000121207 | 1.36177 | 4.457351 | 5.258764 | 2.45E-07 | 1.79E-06 | 6.462475 | LRAT |
| ENSG00000254349 | ENSG00000254349 | 1.364567 | 2.790411 | 6.147976 | 2.03E-09 | 3.09E-08 | 11.06142 | MIR2052HG |
| ENSG00000235142 | ENSG00000235142 | 1.364644 | 6.590073 | 4.573873 | 6.54E-06 | 2.98E-05 | 3.342969 | LINC02532 |
| ENSG00000075461 | ENSG00000075461 | 1.367938 | 6.263415 | 4.308972 | 2.10E-05 | 8.18E-05 | 2.240115 | CACNG4 |
| ENSG00000105852 | ENSG00000105852 | 1.369214 | 6.509923 | 4.553904 | 7.15E-06 | 3.22E-05 | 3.257782 | PON3 |
| ENSG00000123407 | ENSG00000123407 | 1.371732 | 4.042294 | 3.257344 | 0.001228 | 0.002824 | -1.53993 | HOXC12 |
| ENSG00000124721 | ENSG00000124721 | 1.372085 | 4.752462 | 6.673138 | 9.13E-11 | 2.28E-09 | 14.05074 | DNAH8 |
| ENSG00000183036 | ENSG00000183036 | 1.373545 | 4.131648 | 4.700682 | 3.66E-06 | 1.81E-05 | 3.891675 | PCP4 |
| ENSG00000099954 | ENSG00000099954 | 1.373613 | 5.163759 | 7.313881 | 1.61E-12 | 7.81E-11 | 17.95417 | CECR2 |
| ENSG00000170381 | ENSG00000170381 | 1.376319 | 7.073732 | 4.821676 | 2.08E-06 | 1.11E-05 | 4.427591 | SEMA3E |
| ENSG00000273274 | ENSG00000273274 | 1.376529 | 3.373903 | 7.911992 | 2.95E-14 | 2.84E-12 | 21.83573 | ZBTB8B |
| ENSG00000262966 | ENSG00000262966 | 1.377037 | 3.509893 | 7.852939 | 4.42E-14 | 4.05E-12 | 21.44275 | LOC101928266 |
| ENSG00000279355 | ENSG00000279355 | 1.37726 | 4.62115 | 10.24139 | 7.63E-22 | 3.32E-18 | 38.83585 | AGPAT4-IT1 |
| ENSG00000206129 | ENSG00000206129 | 1.37726 | 2.458674 | 6.560678 | 1.80E-10 | 4.03E-09 | 13.39425 | LOC642484 |
| ENSG00000179083 | ENSG00000179083 | 1.377563 | 2.931263 | 5.367215 | 1.41E-07 | 1.12E-06 | 6.990931 | FAM133A |
| ENSG00000162592 | ENSG00000162592 | 1.378059 | 2.159541 | 8.172901 | 4.80E-15 | 6.35E-13 | 23.59667 | CCDC27 |
| ENSG00000206579 | ENSG00000206579 | 1.380136 | 4.867155 | 6.200036 | 1.50E-09 | 2.42E-08 | 11.34897 | XKR4 |
| ENSG00000187398 | ENSG00000187398 | 1.380986 | 4.974111 | 6.554063 | 1.88E-10 | 4.17E-09 | 13.35591 | LUZP2 |
| ENSG00000253671 | ENSG00000253671 | 1.381912 | 5.259074 | 7.740524 | 9.49E-14 | 7.61E-12 | 20.70046 | LOC101930275 |
| ENSG00000134343 | ENSG00000134343 | 1.383838 | 4.10064 | 5.504323 | 6.93E-08 | 6.16E-07 | 7.672179 | ANO3 |
| ENSG00000187021 | ENSG00000187021 | 1.384809 | 3.152827 | 6.080162 | 2.98E-09 | 4.30E-08 | 10.68981 | PNLIPRP1 |
| ENSG00000181433 | ENSG00000181433 | 1.384975 | 2.406948 | 4.446838 | 1.15E-05 | 4.87E-05 | 2.806724 | SAGE1 |
| ENSG00000185842 | ENSG00000185842 | 1.385659 | 7.63249 | 6.980445 | 1.36E-11 | 4.60E-10 | 15.88878 | DNAH14 |
| ENSG00000152785 | ENSG00000152785 | 1.385821 | 5.434451 | 4.128896 | 4.51E-05 | 0.000159 | 1.524369 | BMP3 |
| ENSG00000084453 | ENSG00000084453 | 1.386942 | 3.825933 | 5.44182 | 9.60E-08 | 8.10E-07 | 7.359812 | SLCO1A2 |
| ENSG00000182632 | ENSG00000182632 | 1.387277 | 1.982876 | 6.594925 | 1.47E-10 | 3.41E-09 | 13.59324 | CCNYL2 |
| ENSG00000153132 | ENSG00000153132 | 1.387563 | 5.685763 | 6.140114 | 2.12E-09 | 3.22E-08 | 11.01817 | CLGN |
| ENSG00000256124 | ENSG00000256124 | 1.388507 | 3.613295 | 6.111244 | 2.50E-09 | 3.71E-08 | 10.85972 | LINC01152 |
| ENSG00000143452 | ENSG00000143452 | 1.388963 | 4.817821 | 5.045385 | 7.10E-07 | 4.45E-06 | 5.449903 | HORMAD1 |
| ENSG00000121570 | ENSG00000121570 | 1.389486 | 3.262939 | 7.7441 | 9.26E-14 | 7.49E-12 | 20.72395 | DPPA4 |
| ENSG00000187715 | ENSG00000187715 | 1.389887 | 6.41391 | 4.803549 | 2.27E-06 | 1.20E-05 | 4.346536 | KBTBD12 |
| ENSG00000203799 | ENSG00000203799 | 1.390707 | 6.131832 | 6.725453 | 6.63E-11 | 1.74E-09 | 14.35911 | CCDC162P |
| ENSG00000105641 | ENSG00000105641 | 1.393192 | 6.169369 | 4.384471 | 1.52E-05 | 6.17E-05 | 2.548417 | SLC5A5 |
| ENSG00000160181 | ENSG00000160181 | 1.393813 | 10.00205 | 3.178836 | 0.001603 | 0.003563 | -1.78267 | TFF2 |
| ENSG00000127252 | ENSG00000127252 | 1.393997 | 3.842475 | 5.907677 | 7.85E-09 | 9.77E-08 | 9.759843 | PLAAT1 |
| ENSG00000163295 | ENSG00000163295 | 1.395346 | 5.890989 | 3.943327 | 9.61E-05 | 0.000306 | 0.81596 | ALPI |
| ENSG00000269994 | ENSG00000269994 | 1.398361 | 4.458833 | 5.571177 | 4.87E-08 | 4.61E-07 | 8.00963 | LOC440173 |
| ENSG00000155052 | ENSG00000155052 | 1.399176 | 2.421375 | 6.174071 | 1.75E-09 | 2.73E-08 | 11.20532 | CNTNAP5 |
| ENSG00000180210 | ENSG00000180210 | 1.39998 | 3.699335 | 5.507337 | 6.82E-08 | 6.08E-07 | 7.68732 | F2 |
| ENSG00000212916 | ENSG00000212916 | 1.401426 | 7.4652 | 9.161321 | 3.54E-18 | 2.20E-15 | 30.61204 | MAP10 |
| ENSG00000172139 | ENSG00000172139 | 1.402032 | 5.03287 | 9.742419 | 4.03E-20 | 6.34E-17 | 34.9698 | SLC9C1 |
| ENSG00000240922 | ENSG00000240922 | 1.403912 | 2.639688 | 7.276706 | 2.05E-12 | 9.51E-11 | 17.72031 | LSAMP-AS1 |
| ENSG00000066813 | ENSG00000066813 | 1.4051 | 2.180323 | 7.492668 | 4.99E-13 | 2.98E-11 | 19.09115 | ACSM2B |
| ENSG00000171243 | ENSG00000171243 | 1.405312 | 6.236964 | 4.594456 | 5.95E-06 | 2.75E-05 | 3.431126 | SOSTDC1 |
| ENSG00000162641 | ENSG00000162641 | 1.405676 | 3.449014 | 9.116696 | 4.95E-18 | 2.83E-15 | 30.28421 | AKNAD1 |
| ENSG00000138653 | ENSG00000138653 | 1.405914 | 2.612346 | 6.664395 | 9.63E-11 | 2.39E-09 | 13.99939 | NDST4 |
| ENSG00000198930 | ENSG00000198930 | 1.406106 | 4.670181 | 3.354764 | 0.000876 | 0.002102 | -1.23096 | CSAG1 |
| ENSG00000206557 | ENSG00000206557 | 1.40719 | 3.331153 | 4.964137 | 1.05E-06 | 6.22E-06 | 5.073928 | TRIM71 |
| ENSG00000177459 | ENSG00000177459 | 1.407785 | 6.598689 | 5.334106 | 1.67E-07 | 1.29E-06 | 6.828622 | ERICH5 |
| ENSG00000157703 | ENSG00000157703 | 1.409464 | 3.713428 | 6.861661 | 2.86E-11 | 8.55E-10 | 15.17074 | SVOPL |
| ENSG00000251138 | ENSG00000251138 | 1.411144 | 3.034298 | 6.582073 | 1.58E-10 | 3.62E-09 | 13.51847 | LOC100507377 |
| ENSG00000169031 | ENSG00000169031 | 1.411437 | 6.639944 | 7.045841 | 9.02E-12 | 3.24E-10 | 16.28811 | COL4A3 |
| ENSG00000170074 | ENSG00000170074 | 1.41166 | 3.782171 | 7.496313 | 4.87E-13 | 2.92E-11 | 19.11454 | FAM153A |
| ENSG00000177359 | ENSG00000177359 | 1.413628 | 5.286598 | 7.204941 | 3.27E-12 | 1.41E-10 | 17.27139 | OVOS2 |
| ENSG00000215529 | ENSG00000215529 | 1.414115 | 3.981809 | 7.743092 | 9.33E-14 | 7.53E-12 | 20.71732 | EFCAB8 |
| ENSG00000234352 | ENSG00000234352 | 1.414598 | 2.289741 | 6.910675 | 2.11E-11 | 6.63E-10 | 15.46589 | LOC349160 |
| ENSG00000004846 | ENSG00000004846 | 1.416936 | 3.445489 | 6.757461 | 5.45E-11 | 1.48E-09 | 14.54871 | ABCB5 |
| ENSG00000163116 | ENSG00000163116 | 1.417117 | 3.031179 | 8.82489 | 4.37E-17 | 1.59E-14 | 28.16556 | STPG2 |
| ENSG00000183833 | ENSG00000183833 | 1.417382 | 6.015133 | 6.1076 | 2.55E-09 | 3.78E-08 | 10.83977 | MAATS1 |
| ENSG00000109061 | ENSG00000109061 | 1.417538 | 2.13573 | 7.093045 | 6.68E-12 | 2.55E-10 | 16.57812 | MYH1 |
| ENSG00000078328 | ENSG00000078328 | 1.417977 | 2.570284 | 7.270123 | 2.14E-12 | 9.86E-11 | 17.679 | RBFOX1 |
| ENSG00000151962 | ENSG00000151962 | 1.422514 | 1.926621 | 6.446409 | 3.57E-10 | 7.17E-09 | 12.73624 | RBM46 |
| ENSG00000260386 | ENSG00000260386 | 1.424074 | 3.139193 | 6.730097 | 6.45E-11 | 1.70E-09 | 14.38658 | LDC1P |
| ENSG00000110245 | ENSG00000110245 | 1.425392 | 3.383191 | 4.065047 | 5.87E-05 | 0.0002 | 1.277265 | APOC3 |
| ENSG00000131482 | ENSG00000131482 | 1.425541 | 2.064714 | 7.084315 | 7.07E-12 | 2.67E-10 | 16.52437 | G6PC |
| ENSG00000130988 | ENSG00000130988 | 1.425778 | 6.788385 | 6.411042 | 4.40E-10 | 8.54E-09 | 12.53443 | RGN |
| ENSG00000163689 | ENSG00000163689 | 1.426965 | 6.776832 | 6.935377 | 1.81E-11 | 5.82E-10 | 15.61524 | C3orf67 |
| ENSG00000198185 | ENSG00000198185 | 1.427688 | 6.450163 | 6.432181 | 3.88E-10 | 7.67E-09 | 12.65494 | ZNF334 |
| ENSG00000268864 | ENSG00000268864 | 1.429614 | 1.835103 | 7.676967 | 1.46E-13 | 1.08E-11 | 20.28417 | LOC284379 |
| ENSG00000095627 | ENSG00000095627 | 1.429948 | 3.849703 | 6.264542 | 1.04E-09 | 1.76E-08 | 11.70799 | TDRD1 |
| ENSG00000260372 | ENSG00000260372 | 1.43033 | 2.350846 | 8.307156 | 1.86E-15 | 2.89E-13 | 24.51815 | AQP4-AS1 |
| ENSG00000182050 | ENSG00000182050 | 1.431093 | 3.361692 | 6.58441 | 1.56E-10 | 3.59E-09 | 13.53206 | MGAT4C |
| ENSG00000198597 | ENSG00000198597 | 1.432925 | 4.956713 | 6.704199 | 7.56E-11 | 1.95E-09 | 14.2336 | ZNF536 |
| ENSG00000183747 | ENSG00000183747 | 1.434399 | 2.096599 | 8.480292 | 5.39E-16 | 1.10E-13 | 25.72153 | ACSM2A |
| ENSG00000225449 | ENSG00000225449 | 1.435629 | 2.674738 | 7.401908 | 9.07E-13 | 4.89E-11 | 18.51144 | RAB6C-AS1 |
| ENSG00000187889 | ENSG00000187889 | 1.437518 | 3.138269 | 6.248637 | 1.14E-09 | 1.90E-08 | 11.61919 | FYB2 |
| ENSG00000087495 | ENSG00000087495 | 1.437548 | 5.708103 | 5.967531 | 5.63E-09 | 7.37E-08 | 10.08006 | PHACTR3 |
| ENSG00000203650 | ENSG00000203650 | 1.43861 | 3.41449 | 8.509142 | 4.38E-16 | 9.32E-14 | 25.92367 | LINC01285 |
| ENSG00000185290 | ENSG00000185290 | 1.44045 | 2.813502 | 4.893153 | 1.48E-06 | 8.33E-06 | 4.749813 | NUPR2 |
| ENSG00000256612 | ENSG00000256612 | 1.442861 | 6.358304 | 5.658424 | 3.06E-08 | 3.12E-07 | 8.455183 | CYP2B7P |
| ENSG00000167780 | ENSG00000167780 | 1.443798 | 3.544924 | 5.254999 | 2.50E-07 | 1.82E-06 | 6.444296 | SOAT2 |
| ENSG00000198822 | ENSG00000198822 | 1.444806 | 2.842573 | 7.915077 | 2.88E-14 | 2.79E-12 | 21.85632 | GRM3 |
| ENSG00000152580 | ENSG00000152580 | 1.444885 | 6.491007 | 6.775755 | 4.87E-11 | 1.34E-09 | 14.65738 | IGSF10 |
| ENSG00000262943 | ENSG00000262943 | 1.446228 | 5.616938 | 6.937171 | 1.79E-11 | 5.77E-10 | 15.6261 | ALOX12P2 |
| ENSG00000089250 | ENSG00000089250 | 1.447103 | 4.675036 | 6.191761 | 1.58E-09 | 2.51E-08 | 11.30314 | NOS1 |
| ENSG00000253641 | ENSG00000253641 | 1.449337 | 4.92057 | 7.170712 | 4.07E-12 | 1.69E-10 | 17.05845 | LINCR-0001 |
| ENSG00000214814 | ENSG00000214814 | 1.450029 | 8.420481 | 4.053874 | 6.14E-05 | 0.000208 | 1.234387 | FER1L6 |
| ENSG00000133640 | ENSG00000133640 | 1.451642 | 4.240431 | 5.985703 | 5.08E-09 | 6.77E-08 | 10.17781 | LRRIQ1 |
| ENSG00000224141 | ENSG00000224141 | 1.452031 | 1.606791 | 5.862752 | 1.01E-08 | 1.21E-07 | 9.521244 | MIR548XHG |
| ENSG00000164756 | ENSG00000164756 | 1.452256 | 3.155627 | 6.449924 | 3.49E-10 | 7.05E-09 | 12.75634 | SLC30A8 |
| ENSG00000112319 | ENSG00000112319 | 1.453091 | 6.073396 | 7.456224 | 6.35E-13 | 3.65E-11 | 18.85774 | EYA4 |
| ENSG00000203995 | ENSG00000203995 | 1.454075 | 4.773491 | 6.911086 | 2.11E-11 | 6.61E-10 | 15.46836 | ZYG11A |
| ENSG00000152092 | ENSG00000152092 | 1.454251 | 4.079663 | 5.585357 | 4.52E-08 | 4.32E-07 | 8.081651 | ASTN1 |
| ENSG00000146039 | ENSG00000146039 | 1.454387 | 5.940163 | 4.211403 | 3.19E-05 | 0.000118 | 1.848867 | SLC17A4 |
| ENSG00000172782 | ENSG00000172782 | 1.455323 | 3.816085 | 5.263569 | 2.40E-07 | 1.76E-06 | 6.485695 | FADS6 |
| ENSG00000125823 | ENSG00000125823 | 1.457631 | 2.623273 | 7.558705 | 3.22E-13 | 2.10E-11 | 19.5162 | CSTL1 |
| ENSG00000172461 | ENSG00000172461 | 1.458252 | 6.295849 | 3.789225 | 0.000176 | 0.000521 | 0.250439 | FUT9 |
| ENSG00000235621 | ENSG00000235621 | 1.459859 | 5.363487 | 5.591931 | 4.36E-08 | 4.20E-07 | 8.11509 | LINC00494 |
| ENSG00000184029 | ENSG00000184029 | 1.464599 | 1.784722 | 5.284386 | 2.16E-07 | 1.61E-06 | 6.586494 | DSCR4 |
| ENSG00000072133 | ENSG00000072133 | 1.465259 | 5.555196 | 6.569636 | 1.71E-10 | 3.86E-09 | 13.44622 | RPS6KA6 |
| ENSG00000173678 | ENSG00000173678 | 1.465411 | 2.690245 | 9.394917 | 5.97E-19 | 5.28E-16 | 32.34428 | SPDYE2B |
| ENSG00000156687 | ENSG00000156687 | 1.465609 | 3.317028 | 6.665311 | 9.58E-11 | 2.38E-09 | 14.00477 | UNC5D |
| ENSG00000171759 | ENSG00000171759 | 1.468493 | 4.023675 | 4.838323 | 1.92E-06 | 1.04E-05 | 4.502265 | PAH |
| ENSG00000143512 | ENSG00000143512 | 1.469056 | 3.446991 | 8.2632 | 2.54E-15 | 3.76E-13 | 24.21531 | HHIPL2 |
| ENSG00000173947 | ENSG00000173947 | 1.471899 | 6.465408 | 6.304597 | 8.21E-10 | 1.44E-08 | 11.93243 | PIFO |
| ENSG00000197444 | ENSG00000197444 | 1.472142 | 6.041314 | 4.355054 | 1.72E-05 | 6.89E-05 | 2.427716 | OGDHL |
| ENSG00000197360 | ENSG00000197360 | 1.473051 | 2.539009 | 7.234846 | 2.69E-12 | 1.19E-10 | 17.45806 | ZNF98 |
| ENSG00000231373 | ENSG00000231373 | 1.476128 | 2.603819 | 8.927117 | 2.05E-17 | 9.13E-15 | 28.90276 | GNA14-AS1 |
| ENSG00000171564 | ENSG00000171564 | 1.476936 | 4.372184 | 3.803035 | 0.000167 | 0.000497 | 0.300268 | FGB |
| ENSG00000164659 | ENSG00000164659 | 1.477408 | 8.406477 | 7.327473 | 1.48E-12 | 7.28E-11 | 18.03989 | KIAA1324L |
| ENSG00000149742 | ENSG00000149742 | 1.47818 | 2.600539 | 7.682729 | 1.40E-13 | 1.04E-11 | 20.32181 | SLC22A9 |
| ENSG00000157542 | ENSG00000157542 | 1.48303 | 5.089077 | 7.04511 | 9.06E-12 | 3.25E-10 | 16.28363 | KCNJ6 |
| ENSG00000180287 | ENSG00000180287 | 1.484817 | 2.970622 | 6.449265 | 3.51E-10 | 7.07E-09 | 12.75257 | PLD5 |
| ENSG00000229240 | ENSG00000229240 | 1.485535 | 1.810348 | 7.879204 | 3.69E-14 | 3.45E-12 | 21.61728 | LINC00710 |
| ENSG00000235244 | ENSG00000235244 | 1.487009 | 4.623057 | 6.935147 | 1.81E-11 | 5.83E-10 | 15.61384 | DANT2 |
| ENSG00000155816 | ENSG00000155816 | 1.489789 | 4.96418 | 6.004619 | 4.57E-09 | 6.21E-08 | 10.27981 | FMN2 |
| ENSG00000237596 | ENSG00000237596 | 1.490411 | 3.83341 | 9.357426 | 7.96E-19 | 6.62E-16 | 32.06446 | LOC644135 |
| ENSG00000132464 | ENSG00000132464 | 1.490926 | 4.904546 | 7.386611 | 1.00E-12 | 5.28E-11 | 18.41424 | ENAM |
| ENSG00000153291 | ENSG00000153291 | 1.491512 | 6.894546 | 8.203281 | 3.88E-15 | 5.21E-13 | 23.80428 | SLC25A27 |
| ENSG00000186487 | ENSG00000186487 | 1.495746 | 2.421881 | 7.08306 | 7.12E-12 | 2.68E-10 | 16.51665 | MYT1L |
| ENSG00000143552 | ENSG00000143552 | 1.495938 | 4.279154 | 8.9641 | 1.55E-17 | 7.10E-15 | 29.1708 | NUP210L |
| ENSG00000223658 | ENSG00000223658 | 1.497814 | 4.227562 | 7.838341 | 4.88E-14 | 4.43E-12 | 21.34593 | C1GALT1C1L |
| ENSG00000221986 | ENSG00000221986 | 1.503363 | 3.295244 | 6.028722 | 3.99E-09 | 5.53E-08 | 10.41017 | MYBPHL |
| ENSG00000168955 | ENSG00000168955 | 1.503716 | 8.316676 | 3.770756 | 0.000189 | 0.000553 | 0.184058 | TM4SF20 |
| ENSG00000171495 | ENSG00000171495 | 1.50404 | 2.208086 | 7.141547 | 4.90E-12 | 1.97E-10 | 16.87762 | MROH2B |
| ENSG00000198054 | ENSG00000198054 | 1.504162 | 2.372959 | 4.234826 | 2.89E-05 | 0.000108 | 1.942054 | DSCR8 |
| ENSG00000145864 | ENSG00000145864 | 1.504202 | 5.121887 | 5.506375 | 6.85E-08 | 6.11E-07 | 7.682484 | GABRB2 |
| ENSG00000164627 | ENSG00000164627 | 1.504983 | 4.895146 | 7.995575 | 1.65E-14 | 1.78E-12 | 22.3955 | KIF6 |
| ENSG00000147606 | ENSG00000147606 | 1.505502 | 4.450635 | 8.05712 | 1.08E-14 | 1.25E-12 | 22.81031 | SLC26A7 |
| ENSG00000242715 | ENSG00000242715 | 1.506089 | 3.818729 | 6.319646 | 7.52E-10 | 1.34E-08 | 12.01705 | CCDC169 |
| ENSG00000175329 | ENSG00000175329 | 1.509151 | 4.308223 | 3.82238 | 0.000155 | 0.000465 | 0.370354 | ISX |
| ENSG00000162409 | ENSG00000162409 | 1.510682 | 7.356139 | 5.357546 | 1.48E-07 | 1.17E-06 | 6.943445 | PRKAA2 |
| ENSG00000267313 | ENSG00000267313 | 1.511527 | 2.389144 | 6.235018 | 1.23E-09 | 2.03E-08 | 11.5433 | KC6 |
| ENSG00000124092 | ENSG00000124092 | 1.514726 | 3.263952 | 5.246577 | 2.61E-07 | 1.89E-06 | 6.403672 | CTCFL |
| ENSG00000101443 | ENSG00000101443 | 1.514811 | 9.582351 | 4.459079 | 1.09E-05 | 4.65E-05 | 2.857806 | WFDC2 |
| ENSG00000134812 | ENSG00000134812 | 1.515441 | 5.399868 | 3.787328 | 0.000178 | 0.000524 | 0.243605 | CBLIF |
| ENSG00000178201 | ENSG00000178201 | 1.516945 | 5.400996 | 10.00798 | 4.95E-21 | 1.38E-17 | 37.01372 | VN1R1 |
| ENSG00000081138 | ENSG00000081138 | 1.517958 | 2.245623 | 7.440662 | 7.03E-13 | 3.96E-11 | 18.75833 | CDH7 |
| ENSG00000178965 | ENSG00000178965 | 1.519934 | 3.694971 | 6.095308 | 2.74E-09 | 3.99E-08 | 10.77252 | ERICH3 |
| ENSG00000251209 | ENSG00000251209 | 1.521915 | 2.701492 | 6.964098 | 1.51E-11 | 5.01E-10 | 15.7894 | LINC00923 |
| ENSG00000100557 | ENSG00000100557 | 1.522165 | 5.380041 | 4.588661 | 6.11E-06 | 2.81E-05 | 3.406271 | CCDC198 |
| ENSG00000227906 | ENSG00000227906 | 1.523405 | 2.911844 | 7.389361 | 9.85E-13 | 5.22E-11 | 18.43171 | SNAP25-AS1 |
| ENSG00000139915 | ENSG00000139915 | 1.526175 | 2.32526 | 6.756676 | 5.48E-11 | 1.48E-09 | 14.54404 | MDGA2 |
| ENSG00000166796 | ENSG00000166796 | 1.527445 | 3.657092 | 5.811675 | 1.33E-08 | 1.54E-07 | 9.251805 | LDHC |
| ENSG00000114638 | ENSG00000114638 | 1.527852 | 5.496264 | 4.328664 | 1.93E-05 | 7.61E-05 | 2.320061 | UPK1B |
| ENSG00000163586 | ENSG00000163586 | 1.528275 | 7.733004 | 3.221236 | 0.001389 | 0.003146 | -1.65227 | FABP1 |
| ENSG00000196660 | ENSG00000196660 | 1.528705 | 4.418323 | 5.469105 | 8.33E-08 | 7.19E-07 | 7.4958 | SLC30A10 |
| ENSG00000204889 | ENSG00000204889 | 1.528935 | 2.49366 | 5.92164 | 7.27E-09 | 9.15E-08 | 9.834307 | KRT40 |
| ENSG00000214128 | ENSG00000214128 | 1.53083 | 3.605224 | 6.372905 | 5.50E-10 | 1.03E-08 | 12.31782 | TMEM213 |
| ENSG00000005421 | ENSG00000005421 | 1.531241 | 3.763354 | 6.052631 | 3.49E-09 | 4.93E-08 | 10.53991 | PON1 |
| ENSG00000166473 | ENSG00000166473 | 1.532031 | 4.837776 | 8.461285 | 6.18E-16 | 1.21E-13 | 25.5886 | PKD1L2 |
| ENSG00000231749 | ENSG00000231749 | 1.542093 | 2.935864 | 7.603188 | 2.39E-13 | 1.64E-11 | 19.80405 | ABCA9-AS1 |
| ENSG00000163618 | ENSG00000163618 | 1.542994 | 7.165065 | 5.66778 | 2.91E-08 | 2.98E-07 | 8.503306 | CADPS |
| ENSG00000150627 | ENSG00000150627 | 1.550741 | 5.339352 | 7.11078 | 5.97E-12 | 2.31E-10 | 16.68745 | WDR17 |
| ENSG00000257636 | ENSG00000257636 | 1.554095 | 2.782584 | 6.409047 | 4.45E-10 | 8.62E-09 | 12.52308 | G2E3-AS1 |
| ENSG00000243069 | ENSG00000243069 | 1.557586 | 3.662349 | 7.556748 | 3.26E-13 | 2.11E-11 | 19.50357 | ARHGEF26-AS1 |
| ENSG00000142449 | ENSG00000142449 | 1.562354 | 4.069434 | 6.315586 | 7.70E-10 | 1.37E-08 | 11.9942 | FBN3 |
| ENSG00000154080 | ENSG00000154080 | 1.564272 | 3.93432 | 5.086829 | 5.79E-07 | 3.74E-06 | 5.643734 | CHST9 |
| ENSG00000226476 | ENSG00000226476 | 1.564669 | 4.623612 | 5.597119 | 4.24E-08 | 4.10E-07 | 8.141505 | LINC01748 |
| ENSG00000169302 | ENSG00000169302 | 1.564709 | 4.940229 | 7.391729 | 9.70E-13 | 5.15E-11 | 18.44675 | STK32A |
| ENSG00000175161 | ENSG00000175161 | 1.567617 | 4.385101 | 6.202186 | 1.49E-09 | 2.39E-08 | 11.36089 | CADM2 |
| ENSG00000261787 | ENSG00000261787 | 1.570241 | 3.330679 | 8.519589 | 4.06E-16 | 8.78E-14 | 25.99698 | TCF24 |
| ENSG00000146839 | ENSG00000146839 | 1.571657 | 3.468429 | 7.089775 | 6.82E-12 | 2.60E-10 | 16.55797 | ZAN |
| ENSG00000175497 | ENSG00000175497 | 1.575176 | 3.155625 | 5.722229 | 2.17E-08 | 2.32E-07 | 8.784689 | DPP10 |
| ENSG00000205628 | ENSG00000205628 | 1.575406 | 2.032284 | 5.687932 | 2.61E-08 | 2.72E-07 | 8.607184 | LINC01446 |
| ENSG00000140015 | ENSG00000140015 | 1.576974 | 2.557775 | 6.629936 | 1.19E-10 | 2.86E-09 | 13.79751 | KCNH5 |
| ENSG00000253301 | ENSG00000253301 | 1.578559 | 2.3819 | 6.663604 | 9.68E-11 | 2.40E-09 | 13.99475 | LINC01606 |
| ENSG00000234840 | ENSG00000234840 | 1.579963 | 3.837957 | 6.410548 | 4.41E-10 | 8.56E-09 | 12.53162 | LINC01239 |
| ENSG00000185002 | ENSG00000185002 | 1.581804 | 3.973951 | 5.222553 | 2.95E-07 | 2.10E-06 | 6.288089 | RFX6 |
| ENSG00000230453 | ENSG00000230453 | 1.583293 | 4.122151 | 5.367154 | 1.41E-07 | 1.12E-06 | 6.990636 | ANKRD18B |
| ENSG00000213401 | ENSG00000213401 | 1.583982 | 4.050271 | 3.384697 | 0.000789 | 0.001917 | -1.13431 | MAGEA12 |
| ENSG00000148942 | ENSG00000148942 | 1.584426 | 5.267314 | 6.376189 | 5.40E-10 | 1.02E-08 | 12.33643 | SLC5A12 |
| ENSG00000260230 | ENSG00000260230 | 1.585129 | 5.407864 | 5.49175 | 7.40E-08 | 6.51E-07 | 7.609101 | FRRS1L |
| ENSG00000205592 | ENSG00000205592 | 1.585219 | 3.102895 | 7.081541 | 7.19E-12 | 2.71E-10 | 16.5073 | MUC19 |
| ENSG00000182333 | ENSG00000182333 | 1.585315 | 7.558838 | 2.695859 | 0.00734 | 0.013537 | -3.15181 | LIPF |
| ENSG00000146038 | ENSG00000146038 | 1.586669 | 6.83874 | 5.234414 | 2.78E-07 | 1.99E-06 | 6.345097 | DCDC2 |
| ENSG00000133863 | ENSG00000133863 | 1.587771 | 3.813911 | 5.400474 | 1.19E-07 | 9.68E-07 | 7.154841 | TEX15 |
| ENSG00000122711 | ENSG00000122711 | 1.58779 | 7.21006 | 3.975818 | 8.43E-05 | 0.000273 | 0.937838 | SPINK4 |
| ENSG00000139219 | ENSG00000139219 | 1.588862 | 3.857147 | 5.063899 | 6.48E-07 | 4.11E-06 | 5.536321 | COL2A1 |
| ENSG00000125851 | ENSG00000125851 | 1.59087 | 3.92484 | 6.086309 | 2.88E-09 | 4.18E-08 | 10.72336 | PCSK2 |
| ENSG00000153157 | ENSG00000153157 | 1.593672 | 4.121141 | 8.075817 | 9.48E-15 | 1.12E-12 | 22.93677 | SYCP2L |
| ENSG00000259439 | ENSG00000259439 | 1.59725 | 5.873419 | 4.86268 | 1.71E-06 | 9.44E-06 | 4.611928 | LINC01833 |
| ENSG00000146477 | ENSG00000146477 | 1.597834 | 8.925795 | 7.018495 | 1.07E-11 | 3.76E-10 | 16.12078 | SLC22A3 |
| ENSG00000118137 | ENSG00000118137 | 1.599079 | 6.617102 | 4.257847 | 2.62E-05 | 9.89E-05 | 2.034095 | APOA1 |
| ENSG00000120669 | ENSG00000120669 | 1.599956 | 2.480571 | 6.24683 | 1.15E-09 | 1.91E-08 | 11.60912 | SOHLH2 |
| ENSG00000115850 | ENSG00000115850 | 1.605543 | 3.445474 | 6.629267 | 1.19E-10 | 2.86E-09 | 13.7936 | LCT |
| ENSG00000171815 | ENSG00000171815 | 1.605546 | 2.987311 | 8.059268 | 1.06E-14 | 1.24E-12 | 22.82483 | PCDHB1 |
| ENSG00000225548 | ENSG00000225548 | 1.612513 | 2.794293 | 4.904299 | 1.41E-06 | 7.96E-06 | 4.800439 | LINC01980 |
| ENSG00000134240 | ENSG00000134240 | 1.613326 | 10.51515 | 4.123889 | 4.60E-05 | 0.000162 | 1.504863 | HMGCS2 |
| ENSG00000118432 | ENSG00000118432 | 1.618223 | 6.441046 | 5.853239 | 1.06E-08 | 1.27E-07 | 9.470914 | CNR1 |
| ENSG00000124260 | ENSG00000124260 | 1.618441 | 2.341618 | 4.556967 | 7.05E-06 | 3.19E-05 | 3.270827 | MAGEA10 |
| ENSG00000101115 | ENSG00000101115 | 1.619569 | 7.505101 | 6.908578 | 2.14E-11 | 6.69E-10 | 15.45322 | SALL4 |
| ENSG00000227674 | ENSG00000227674 | 1.622795 | 2.870545 | 5.245382 | 2.63E-07 | 1.90E-06 | 6.397911 | LINC00355 |
| ENSG00000188738 | ENSG00000188738 | 1.625372 | 6.801071 | 7.901609 | 3.16E-14 | 3.02E-12 | 21.76648 | FSIP2 |
| ENSG00000170289 | ENSG00000170289 | 1.629667 | 3.917688 | 8.49892 | 4.72E-16 | 9.96E-14 | 25.85199 | CNGB3 |
| ENSG00000171714 | ENSG00000171714 | 1.631096 | 7.697619 | 6.795783 | 4.31E-11 | 1.21E-09 | 14.77662 | ANO5 |
| ENSG00000196109 | ENSG00000196109 | 1.633971 | 3.477548 | 8.000248 | 1.60E-14 | 1.73E-12 | 22.42692 | ZNF676 |
| ENSG00000081479 | ENSG00000081479 | 1.635122 | 4.302624 | 5.93302 | 6.82E-09 | 8.69E-08 | 9.8951 | LRP2 |
| ENSG00000091138 | ENSG00000091138 | 1.65079 | 6.098604 | 4.37315 | 1.59E-05 | 6.45E-05 | 2.501877 | SLC26A3 |
| ENSG00000240893 | ENSG00000240893 | 1.657296 | 2.196708 | 8.403776 | 9.34E-16 | 1.69E-13 | 25.18764 | LINC02042 |
| ENSG00000164199 | ENSG00000164199 | 1.657351 | 5.654821 | 7.302731 | 1.73E-12 | 8.30E-11 | 17.88394 | ADGRV1 |
| ENSG00000133454 | ENSG00000133454 | 1.657936 | 4.625363 | 6.182783 | 1.66E-09 | 2.62E-08 | 11.25346 | MYO18B |
| ENSG00000146530 | ENSG00000146530 | 1.659254 | 3.492167 | 6.414867 | 4.30E-10 | 8.37E-09 | 12.55621 | VWDE |
| ENSG00000141449 | ENSG00000141449 | 1.65961 | 6.396126 | 7.31801 | 1.57E-12 | 7.66E-11 | 17.9802 | GREB1L |
| ENSG00000152430 | ENSG00000152430 | 1.664648 | 3.35037 | 9.320058 | 1.06E-18 | 8.32E-16 | 31.78623 | BOLL |
| ENSG00000185942 | ENSG00000185942 | 1.668997 | 2.827198 | 8.360702 | 1.27E-15 | 2.15E-13 | 24.88852 | NKAIN3 |
| ENSG00000263711 | ENSG00000263711 | 1.674749 | 3.422955 | 4.599996 | 5.81E-06 | 2.69E-05 | 3.454914 | LINC02864 |
| ENSG00000167798 | ENSG00000167798 | 1.678732 | 4.628356 | 5.417834 | 1.09E-07 | 8.99E-07 | 7.240743 | C3P1 |
| ENSG00000229404 | ENSG00000229404 | 1.696727 | 4.749529 | 6.16734 | 1.82E-09 | 2.82E-08 | 11.16815 | LINC00858 |
| ENSG00000274956 | ENSG00000274956 | 1.69693 | 3.711994 | 7.204009 | 3.28E-12 | 1.42E-10 | 17.26558 | NKAIN3-IT1 |
| ENSG00000147246 | ENSG00000147246 | 1.702107 | 2.390443 | 5.822006 | 1.26E-08 | 1.46E-07 | 9.306143 | HTR2C |
| ENSG00000204740 | ENSG00000204740 | 1.703085 | 5.873497 | 4.886193 | 1.53E-06 | 8.56E-06 | 4.718256 | MALRD1 |
| ENSG00000196917 | ENSG00000196917 | 1.704928 | 6.494978 | 6.888343 | 2.43E-11 | 7.45E-10 | 15.33121 | HCAR1 |
| ENSG00000250546 | ENSG00000250546 | 1.705328 | 2.276653 | 6.921769 | 1.97E-11 | 6.24E-10 | 15.53291 | LOC101928978 |
| ENSG00000183117 | ENSG00000183117 | 1.707023 | 4.094506 | 6.465971 | 3.17E-10 | 6.53E-09 | 12.84823 | CSMD1 |
| ENSG00000067715 | ENSG00000067715 | 1.709844 | 7.476735 | 6.88463 | 2.48E-11 | 7.59E-10 | 15.30885 | SYT1 |
| ENSG00000154997 | ENSG00000154997 | 1.713003 | 2.09677 | 8.104011 | 7.78E-15 | 9.66E-13 | 23.12785 | SEPTIN14 |
| ENSG00000179914 | ENSG00000179914 | 1.715082 | 7.287679 | 4.412281 | 1.34E-05 | 5.56E-05 | 2.663192 | ITLN1 |
| ENSG00000165863 | ENSG00000165863 | 1.716677 | 3.184468 | 6.714658 | 7.09E-11 | 1.85E-09 | 14.29532 | C10orf82 |
| ENSG00000143469 | ENSG00000143469 | 1.729552 | 4.278394 | 8.201473 | 3.93E-15 | 5.26E-13 | 23.79191 | SYT14 |
| ENSG00000150893 | ENSG00000150893 | 1.731809 | 7.368033 | 5.554651 | 5.31E-08 | 4.95E-07 | 7.925897 | FREM2 |
| ENSG00000228697 | ENSG00000228697 | 1.734927 | 2.470745 | 8.628212 | 1.85E-16 | 4.89E-14 | 26.76278 | LOC101928565 |
| ENSG00000152910 | ENSG00000152910 | 1.736156 | 2.426291 | 7.227448 | 2.82E-12 | 1.24E-10 | 17.41183 | CNTNAP4 |
| ENSG00000110244 | ENSG00000110244 | 1.743323 | 4.18624 | 4.086601 | 5.37E-05 | 0.000185 | 1.360288 | APOA4 |
| ENSG00000009694 | ENSG00000009694 | 1.74424 | 5.899737 | 7.797326 | 6.45E-14 | 5.57E-12 | 21.07458 | TENM1 |
| ENSG00000138207 | ENSG00000138207 | 1.746667 | 8.323727 | 5.806724 | 1.37E-08 | 1.57E-07 | 9.22579 | RBP4 |
| ENSG00000124134 | ENSG00000124134 | 1.751757 | 4.685792 | 7.27862 | 2.03E-12 | 9.44E-11 | 17.73234 | KCNS1 |
| ENSG00000249267 | ENSG00000249267 | 1.761339 | 4.433684 | 7.804517 | 6.14E-14 | 5.35E-12 | 21.12207 | LINC00939 |
| ENSG00000187772 | ENSG00000187772 | 1.763889 | 2.046328 | 5.702917 | 2.41E-08 | 2.54E-07 | 8.684631 | LIN28B |
| ENSG00000229876 | ENSG00000229876 | 1.766529 | 2.25566 | 7.699136 | 1.26E-13 | 9.52E-12 | 20.4291 | CASC20 |
| ENSG00000173157 | ENSG00000173157 | 1.771194 | 2.441198 | 6.959235 | 1.56E-11 | 5.15E-10 | 15.75987 | ADAMTS20 |
| ENSG00000118271 | ENSG00000118271 | 1.774521 | 5.610033 | 5.088563 | 5.74E-07 | 3.71E-06 | 5.651872 | TTR |
| ENSG00000102313 | ENSG00000102313 | 1.784248 | 3.292464 | 7.756708 | 8.50E-14 | 7.02E-12 | 20.80685 | ITIH6 |
| ENSG00000183273 | ENSG00000183273 | 1.785952 | 3.439053 | 8.446011 | 6.90E-16 | 1.32E-13 | 25.48193 | CCDC60 |
| ENSG00000185306 | ENSG00000185306 | 1.790184 | 4.319076 | 6.134604 | 2.19E-09 | 3.31E-08 | 10.98788 | C12orf56 |
| ENSG00000182256 | ENSG00000182256 | 1.812089 | 2.684064 | 7.752303 | 8.76E-14 | 7.16E-12 | 20.77788 | GABRG3 |
| ENSG00000251165 | ENSG00000251165 | 1.813843 | 3.154547 | 7.295706 | 1.82E-12 | 8.62E-11 | 17.83973 | F11-AS1 |
| ENSG00000155761 | ENSG00000155761 | 1.836575 | 4.840948 | 8.325716 | 1.63E-15 | 2.62E-13 | 24.64634 | SPAG17 |
| ENSG00000175928 | ENSG00000175928 | 1.837572 | 7.059428 | 6.25662 | 1.09E-09 | 1.83E-08 | 11.66374 | LRRN1 |
| ENSG00000011677 | ENSG00000011677 | 1.837634 | 4.263597 | 4.845861 | 1.86E-06 | 1.01E-05 | 4.536151 | GABRA3 |
| ENSG00000157423 | ENSG00000157423 | 1.841822 | 4.172799 | 9.444973 | 4.06E-19 | 4.11E-16 | 32.71894 | HYDIN |
| ENSG00000159217 | ENSG00000159217 | 1.849624 | 6.092536 | 5.05176 | 6.88E-07 | 4.33E-06 | 5.479631 | IGF2BP1 |
| ENSG00000215146 | ENSG00000215146 | 1.849629 | 5.519498 | 8.522549 | 3.98E-16 | 8.69E-14 | 26.01776 | LOC441666 |
| ENSG00000119121 | ENSG00000119121 | 1.853211 | 6.045056 | 8.696828 | 1.12E-16 | 3.27E-14 | 27.24982 | TRPM6 |
| ENSG00000146166 | ENSG00000146166 | 1.85876 | 3.702345 | 5.835053 | 1.17E-08 | 1.38E-07 | 9.374884 | LGSN |
| ENSG00000110195 | ENSG00000110195 | 1.860987 | 6.77011 | 5.591921 | 4.36E-08 | 4.20E-07 | 8.115038 | FOLR1 |
| ENSG00000259129 | ENSG00000259129 | 1.877151 | 2.438962 | 6.311574 | 7.88E-10 | 1.40E-08 | 11.97164 | LINC00648 |
| ENSG00000124143 | ENSG00000124143 | 1.903469 | 4.218477 | 6.502892 | 2.55E-10 | 5.42E-09 | 13.06035 | ARHGAP40 |
| ENSG00000088386 | ENSG00000088386 | 1.926213 | 6.537656 | 5.747047 | 1.90E-08 | 2.07E-07 | 8.913689 | SLC15A1 |
| ENSG00000147381 | ENSG00000147381 | 1.937007 | 3.211388 | 4.119076 | 4.69E-05 | 0.000165 | 1.486137 | MAGEA4 |
| ENSG00000215808 | ENSG00000215808 | 1.946936 | 2.807042 | 7.085264 | 7.02E-12 | 2.65E-10 | 16.53021 | LINC01139 |
| ENSG00000106536 | ENSG00000106536 | 1.961161 | 4.769036 | 5.997179 | 4.77E-09 | 6.43E-08 | 10.23966 | POU6F2 |
| ENSG00000127928 | ENSG00000127928 | 1.962683 | 3.965184 | 5.908966 | 7.80E-09 | 9.71E-08 | 9.766707 | GNGT1 |
| ENSG00000186472 | ENSG00000186472 | 1.964077 | 7.804301 | 6.536563 | 2.08E-10 | 4.56E-09 | 13.25463 | PCLO |
| ENSG00000084674 | ENSG00000084674 | 1.980397 | 6.811322 | 4.943235 | 1.17E-06 | 6.78E-06 | 4.978067 | APOB |
| ENSG00000158816 | ENSG00000158816 | 1.991204 | 3.752451 | 7.63661 | 1.91E-13 | 1.37E-11 | 20.02113 | VWA5B1 |
| ENSG00000221867 | ENSG00000221867 | 2.026106 | 4.913651 | 3.947289 | 9.46E-05 | 0.000302 | 0.830772 | MAGEA3 |
| ENSG00000146276 | ENSG00000146276 | 2.034219 | 3.687007 | 7.052138 | 8.67E-12 | 3.14E-10 | 16.32671 | GABRR1 |
| ENSG00000197172 | ENSG00000197172 | 2.106386 | 4.419369 | 4.268437 | 2.50E-05 | 9.50E-05 | 2.076588 | MAGEA6 |
| ENSG00000236824 | ENSG00000236824 | 2.118183 | 9.357233 | 10.84223 | 5.59E-24 | 1.58E-19 | 43.63008 | BCYRN1 |
| ENSG00000259664 | ENSG00000259664 | 2.132434 | 2.728395 | 8.613642 | 2.05E-16 | 5.26E-14 | 26.65968 | LINC02254 |

Table S3. Difflab Cluster C

|  |  |  |  |  |  |  |  |  |
| --- | --- | --- | --- | --- | --- | --- | --- | --- |
|  | ensembl | logFC | AveExpr | t | P.Value | adj.P.Val | B | gene_id |
| ENSG00000106809 | ENSG00000106809 | -1.90104 | 8.984186 | -6.57716 | 1.63E-10 | 3.04E-08 | 13.53472 | OGN |
| ENSG00000237125 | ENSG00000237125 | -1.78659 | 6.332477 | -5.88613 | 8.85E-09 | 6.70E-07 | 9.725189 | HAND2-AS1 |
| ENSG00000119147 | ENSG00000119147 | -1.7513 | 6.392159 | -5.65729 | 3.08E-08 | 1.82E-06 | 8.54008 | ECRG4 |
| ENSG00000034971 | ENSG00000034971 | -1.73709 | 4.261836 | -5.26512 | 2.38E-07 | 9.70E-06 | 6.601438 | MYOC |
| ENSG00000164122 | ENSG00000164122 | -1.71142 | 3.892988 | -4.91614 | 1.33E-06 | 3.85E-05 | 4.977069 | ASB5 |
| ENSG00000136546 | ENSG00000136546 | -1.71022 | 6.00814 | -6.29062 | 8.91E-10 | 1.15E-07 | 11.91384 | SCN7A |
| ENSG00000181072 | ENSG00000181072 | -1.70263 | 5.470621 | -4.94739 | 1.14E-06 | 3.41E-05 | 5.118576 | CHRM2 |
| ENSG00000144891 | ENSG00000144891 | -1.69252 | 5.080071 | -6.16447 | 1.85E-09 | 2.01E-07 | 11.21859 | AGTR1 |
| ENSG00000164106 | ENSG00000164106 | -1.65838 | 6.60215 | -6.22143 | 1.33E-09 | 1.55E-07 | 11.5311 | SCRG1 |
| ENSG00000018625 | ENSG00000018625 | -1.65693 | 6.831986 | -6.03574 | 3.84E-09 | 3.55E-07 | 10.5209 | ATP1A2 |
| ENSG00000104332 | ENSG00000104332 | -1.65521 | 8.205661 | -6.046 | 3.62E-09 | 3.38E-07 | 10.57603 | SFRP1 |
| ENSG00000091482 | ENSG00000091482 | -1.64956 | 3.734088 | -5.25144 | 2.55E-07 | 1.03E-05 | 6.535921 | SMPX |
| ENSG00000101938 | ENSG00000101938 | -1.64908 | 7.570337 | -5.15197 | 4.20E-07 | 1.53E-05 | 6.064194 | CHRDL1 |
| ENSG00000115593 | ENSG00000115593 | -1.64611 | 4.279111 | -4.49664 | 9.24E-06 | 0.000185 | 3.154223 | SMYD1 |
| ENSG00000111262 | ENSG00000111262 | -1.6288 | 3.116088 | -5.807 | 1.37E-08 | 9.48E-07 | 9.310989 | KCNA1 |
| ENSG00000152785 | ENSG00000152785 | -1.6197 | 5.434451 | -4.94276 | 1.17E-06 | 3.47E-05 | 5.097576 | BMP3 |
| ENSG00000260230 | ENSG00000260230 | -1.6193 | 5.407864 | -5.70964 | 2.32E-08 | 1.46E-06 | 8.80773 | FRRS1L |
| ENSG00000018236 | ENSG00000018236 | -1.6144 | 7.072564 | -6.06255 | 3.30E-09 | 3.14E-07 | 10.66522 | CNTN1 |
| ENSG00000156113 | ENSG00000156113 | -1.59033 | 10.04129 | -6.74318 | 5.95E-11 | 1.41E-08 | 14.4998 | KCNMA1 |
| ENSG00000164107 | ENSG00000164107 | -1.58759 | 7.230682 | -4.88263 | 1.56E-06 | 4.39E-05 | 4.826193 | HAND2 |
| ENSG00000127083 | ENSG00000127083 | -1.57549 | 7.187119 | -6.29525 | 8.67E-10 | 1.14E-07 | 11.93956 | OMD |
| ENSG00000116194 | ENSG00000116194 | -1.57007 | 7.177835 | -6.46804 | 3.14E-10 | 5.00E-08 | 12.9107 | ANGPTL1 |
| ENSG00000175161 | ENSG00000175161 | -1.55657 | 4.385101 | -6.25361 | 1.10E-09 | 1.34E-07 | 11.70866 | CADM2 |
| ENSG00000165966 | ENSG00000165966 | -1.53999 | 5.622652 | -5.86886 | 9.73E-09 | 7.23E-07 | 9.634361 | PDZRN4 |
| ENSG00000146469 | ENSG00000146469 | -1.5277 | 4.884692 | -5.19909 | 3.32E-07 | 1.26E-05 | 6.286686 | VIP |
| ENSG00000138650 | ENSG00000138650 | -1.52756 | 4.637777 | -5.68046 | 2.72E-08 | 1.65E-06 | 8.658279 | PCDH10 |
| ENSG00000224958 | ENSG00000224958 | -1.51682 | 3.151234 | -6.09679 | 2.72E-09 | 2.70E-07 | 10.85027 | PGM5-AS1 |
| ENSG00000118729 | ENSG00000118729 | -1.5156 | 6.318488 | -5.88035 | 9.14E-09 | 6.86E-07 | 9.694756 | CASQ2 |
| ENSG00000198523 | ENSG00000198523 | -1.51474 | 8.720422 | -6.2756 | 9.72E-10 | 1.22E-07 | 11.83044 | PLN |
| ENSG00000082482 | ENSG00000082482 | -1.50927 | 4.783134 | -5.83593 | 1.17E-08 | 8.34E-07 | 9.461845 | KCNK2 |
| ENSG00000071991 | ENSG00000071991 | -1.50593 | 5.302938 | -5.2143 | 3.07E-07 | 1.19E-05 | 6.358914 | CDH19 |
| ENSG00000163017 | ENSG00000163017 | -1.50565 | 12.80662 | -5.17298 | 3.78E-07 | 1.41E-05 | 6.163188 | ACTG2 |
| ENSG00000184905 | ENSG00000184905 | -1.50044 | 5.247066 | -4.83792 | 1.93E-06 | 5.23E-05 | 4.626305 | TCEAL2 |
| ENSG00000004776 | ENSG00000004776 | -1.49527 | 10.65607 | -5.15839 | 4.06E-07 | 1.49E-05 | 6.09441 | HSPB6 |
| ENSG00000183230 | ENSG00000183230 | -1.49488 | 5.385609 | -5.50638 | 6.85E-08 | 3.49E-06 | 7.780113 | CTNNA3 |
| ENSG00000172403 | ENSG00000172403 | -1.48408 | 12.0249 | -5.54094 | 5.71E-08 | 3.02E-06 | 7.952649 | SYNPO2 |
| ENSG00000165072 | ENSG00000165072 | -1.47267 | 6.883559 | -6.07691 | 3.04E-09 | 2.96E-07 | 10.7427 | MAMDC2 |
| ENSG00000123560 | ENSG00000123560 | -1.47064 | 4.987143 | -5.30625 | 1.93E-07 | 8.19E-06 | 6.799187 | PLP1 |
| ENSG00000112936 | ENSG00000112936 | -1.45651 | 9.450982 | -4.54601 | 7.41E-06 | 0.000156 | 3.361272 | C7 |
| ENSG00000123572 | ENSG00000123572 | -1.44804 | 6.072679 | -5.33616 | 1.66E-07 | 7.20E-06 | 6.943844 | NRK |
| ENSG00000089250 | ENSG00000089250 | -1.44002 | 4.675036 | -6.25805 | 1.08E-09 | 1.32E-07 | 11.73326 | NOS1 |
| ENSG00000133392 | ENSG00000133392 | -1.43614 | 14.04726 | -4.73712 | 3.09E-06 | 7.65E-05 | 4.181627 | MYH11 |
| ENSG00000149970 | ENSG00000149970 | -1.4338 | 5.178263 | -5.94374 | 6.43E-09 | 5.27E-07 | 10.02962 | CNKSR2 |
| ENSG00000114200 | ENSG00000114200 | -1.43335 | 5.697823 | -6.13194 | 2.22E-09 | 2.29E-07 | 11.04114 | BCHE |
| ENSG00000126950 | ENSG00000126950 | -1.41095 | 5.326072 | -5.84168 | 1.13E-08 | 8.17E-07 | 9.491911 | TMEM35A |
| ENSG00000182253 | ENSG00000182253 | -1.40742 | 10.85066 | -5.20504 | 3.22E-07 | 1.23E-05 | 6.314916 | SYNM |
| ENSG00000075073 | ENSG00000075073 | -1.4048 | 7.602873 | -5.12384 | 4.82E-07 | 1.71E-05 | 5.932239 | TACR2 |
| ENSG00000196616 | ENSG00000196616 | -1.40342 | 8.367992 | -4.43551 | 1.21E-05 | 0.000231 | 2.900664 | ADH1B |
| ENSG00000118432 | ENSG00000118432 | -1.40058 | 6.441046 | -5.08679 | 5.79E-07 | 1.98E-05 | 5.759336 | CNR1 |
| ENSG00000130176 | ENSG00000130176 | -1.39414 | 11.48291 | -5.06364 | 6.49E-07 | 2.17E-05 | 5.651836 | CNN1 |
| ENSG00000175084 | ENSG00000175084 | -1.38854 | 12.18063 | -3.56383 | 0.000413 | 0.003938 | -0.36944 | DES |
| ENSG00000165300 | ENSG00000165300 | -1.3883 | 5.473641 | -5.25387 | 2.52E-07 | 1.02E-05 | 6.547568 | SLITRK5 |
| ENSG00000167676 | ENSG00000167676 | -1.36529 | 8.193538 | -5.06517 | 6.44E-07 | 2.15E-05 | 5.65893 | PLIN4 |
| ENSG00000253250 | ENSG00000253250 | -1.36528 | 5.237245 | -6.92629 | 1.91E-11 | 5.37E-09 | 15.58581 | C8orf88 |
| ENSG00000113296 | ENSG00000113296 | -1.36353 | 9.559983 | -4.37164 | 1.60E-05 | 0.000291 | 2.639112 | THBS4 |
| ENSG00000158445 | ENSG00000158445 | -1.34749 | 5.677183 | -5.10391 | 5.32E-07 | 1.85E-05 | 5.839086 | KCNB1 |
| ENSG00000139330 | ENSG00000139330 | -1.34462 | 3.350097 | -5.94726 | 6.30E-09 | 5.21E-07 | 10.04833 | KERA |
| ENSG00000179915 | ENSG00000179915 | -1.34331 | 5.136062 | -5.46547 | 8.49E-08 | 4.14E-06 | 7.577096 | NRXN1 |
| ENSG00000129596 | ENSG00000129596 | -1.34059 | 5.525873 | -6.60279 | 1.40E-10 | 2.74E-08 | 13.6825 | CDO1 |
| ENSG00000130226 | ENSG00000130226 | -1.3353 | 4.700891 | -5.07159 | 6.24E-07 | 2.10E-05 | 5.688704 | DPP6 |
| ENSG00000163377 | ENSG00000163377 | -1.33093 | 2.903123 | -4.71758 | 3.39E-06 | 8.24E-05 | 4.096353 | TAFA4 |
| ENSG00000184226 | ENSG00000184226 | -1.32972 | 6.776634 | -5.97822 | 5.30E-09 | 4.51E-07 | 10.213 | PCDH9 |
| ENSG00000122367 | ENSG00000122367 | -1.32777 | 7.255996 | -5.80659 | 1.37E-08 | 9.48E-07 | 9.308818 | LDB3 |
| ENSG00000135298 | ENSG00000135298 | -1.32728 | 4.553406 | -6.30149 | 8.36E-10 | 1.10E-07 | 11.97429 | ADGRB3 |
| ENSG00000138675 | ENSG00000138675 | -1.32618 | 3.539854 | -5.88454 | 8.93E-09 | 6.74E-07 | 9.716789 | FGF5 |
| ENSG00000145423 | ENSG00000145423 | -1.31583 | 11.41839 | -4.14794 | 4.16E-05 | 0.000627 | 1.749942 | SFRP2 |
| ENSG00000255248 | ENSG00000255248 | -1.30313 | 8.887145 | -6.81531 | 3.82E-11 | 9.69E-09 | 14.92493 | MIR100HG |
| ENSG00000170323 | ENSG00000170323 | -1.3011 | 6.238871 | -5.53526 | 5.89E-08 | 3.08E-06 | 7.92425 | FABP4 |
| ENSG00000229240 | ENSG00000229240 | -1.29822 | 1.810348 | -6.86496 | 2.81E-11 | 7.35E-09 | 15.2196 | LINC00710 |
| ENSG00000154330 | ENSG00000154330 | -1.2963 | 9.303145 | -5.35509 | 1.50E-07 | 6.64E-06 | 7.035703 | PGM5 |
| ENSG00000226237 | ENSG00000226237 | -1.29591 | 3.783162 | -7.11568 | 5.79E-12 | 2.05E-09 | 16.73242 | GAS1RR |
| ENSG00000138061 | ENSG00000138061 | -1.29518 | 9.884692 | -5.61416 | 3.87E-08 | 2.19E-06 | 8.321155 | CYP1B1 |
| ENSG00000147588 | ENSG00000147588 | -1.28731 | 2.794651 | -6.14497 | 2.06E-09 | 2.17E-07 | 11.11213 | PMP2 |
| ENSG00000237515 | ENSG00000237515 | -1.28692 | 3.649865 | -5.53511 | 5.89E-08 | 3.08E-06 | 7.923464 | SHISA9 |
| ENSG00000106511 | ENSG00000106511 | -1.28255 | 6.302341 | -5.4702 | 8.28E-08 | 4.05E-06 | 7.600523 | MEOX2 |
| ENSG00000173641 | ENSG00000173641 | -1.28056 | 8.975693 | -4.70586 | 3.57E-06 | 8.56E-05 | 4.045381 | HSPB7 |
| ENSG00000206579 | ENSG00000206579 | -1.27179 | 4.867155 | -5.75965 | 1.77E-08 | 1.17E-06 | 9.065348 | XKR4 |
| ENSG00000169085 | ENSG00000169085 | -1.26905 | 4.141027 | -6.55903 | 1.82E-10 | 3.33E-08 | 13.4305 | VXN |
| ENSG00000185985 | ENSG00000185985 | -1.26714 | 3.381473 | -6.26511 | 1.03E-09 | 1.27E-07 | 11.77234 | SLITRK2 |
| ENSG00000185681 | ENSG00000185681 | -1.26651 | 2.982808 | -4.6886 | 3.87E-06 | 9.11E-05 | 3.970484 | MORN5 |
| ENSG00000241684 | ENSG00000241684 | -1.25959 | 5.434684 | -7.16412 | 4.24E-12 | 1.63E-09 | 17.02948 | ADAMTS9-AS2 |
| ENSG00000106772 | ENSG00000106772 | -1.25368 | 10.61468 | -4.93233 | 1.23E-06 | 3.62E-05 | 5.050304 | PRUNE2 |
| ENSG00000124491 | ENSG00000124491 | -1.25142 | 9.274193 | -5.70502 | 2.38E-08 | 1.49E-06 | 8.784016 | F13A1 |
| ENSG00000164326 | ENSG00000164326 | -1.2498 | 3.061776 | -3.92841 | 0.000102 | 0.001287 | 0.918778 | CARTPT |
| ENSG00000111341 | ENSG00000111341 | -1.24916 | 12.78794 | -6.51172 | 2.42E-10 | 4.06E-08 | 13.15952 | MGP |
| ENSG00000135333 | ENSG00000135333 | -1.24039 | 6.87527 | -4.50371 | 8.96E-06 | 0.000181 | 3.183762 | EPHA7 |
| ENSG00000140285 | ENSG00000140285 | -1.23365 | 9.513409 | -6.52947 | 2.17E-10 | 3.77E-08 | 13.26097 | FGF7 |
| ENSG00000128573 | ENSG00000128573 | -1.23253 | 8.021528 | -5.30417 | 1.95E-07 | 8.25E-06 | 6.789161 | FOXP2 |
| ENSG00000163394 | ENSG00000163394 | -1.23177 | 2.956576 | -4.3175 | 2.03E-05 | 0.000352 | 2.420045 | CCKAR |
| ENSG00000152377 | ENSG00000152377 | -1.23144 | 10.05911 | -5.39877 | 1.20E-07 | 5.48E-06 | 7.248815 | SPOCK1 |
| ENSG00000179639 | ENSG00000179639 | -1.23085 | 5.200355 | -5.27661 | 2.24E-07 | 9.25E-06 | 6.656553 | FCER1A |
| ENSG00000181778 | ENSG00000181778 | -1.22614 | 3.982878 | -4.8582 | 1.75E-06 | 4.84E-05 | 4.716807 | TMEM252 |
| ENSG00000050030 | ENSG00000050030 | -1.22591 | 4.853524 | -5.36689 | 1.41E-07 | 6.29E-06 | 7.093135 | NEXMIF |
| ENSG00000134533 | ENSG00000134533 | -1.22541 | 8.464028 | -6.92914 | 1.88E-11 | 5.33E-09 | 15.6029 | RERG |
| ENSG00000180875 | ENSG00000180875 | -1.21544 | 7.416711 | -4.21569 | 3.13E-05 | 0.000498 | 2.014773 | GREM2 |
| ENSG00000040731 | ENSG00000040731 | -1.21208 | 2.43434 | -5.57464 | 4.78E-08 | 2.58E-06 | 8.121727 | CDH10 |
| ENSG00000123119 | ENSG00000123119 | -1.21207 | 6.494362 | -6.94431 | 1.71E-11 | 5.00E-09 | 15.69391 | NECAB1 |
| ENSG00000198300 | ENSG00000198300 | -1.21124 | 6.904847 | -6.4796 | 2.93E-10 | 4.74E-08 | 12.97642 | PEG3 |
| ENSG00000162706 | ENSG00000162706 | -1.20558 | 5.855597 | -4.41791 | 1.31E-05 | 0.000247 | 2.82825 | CADM3 |
| ENSG00000166292 | ENSG00000166292 | -1.20464 | 6.25219 | -5.42535 | 1.05E-07 | 4.88E-06 | 7.379224 | TMEM100 |
| ENSG00000175785 | ENSG00000175785 | -1.20159 | 6.997587 | -4.16673 | 3.85E-05 | 0.000589 | 1.823021 | PRIMA1 |
| ENSG00000163431 | ENSG00000163431 | -1.1987 | 10.71815 | -5.02619 | 7.80E-07 | 2.51E-05 | 5.478874 | LMOD1 |
| ENSG00000022267 | ENSG00000022267 | -1.19708 | 11.46183 | -5.98802 | 5.02E-09 | 4.38E-07 | 10.26531 | FHL1 |
| ENSG00000141338 | ENSG00000141338 | -1.19698 | 8.019223 | -5.01192 | 8.36E-07 | 2.64E-05 | 5.413285 | ABCA8 |
| ENSG00000166407 | ENSG00000166407 | -1.19445 | 2.838818 | -4.46493 | 1.06E-05 | 0.000207 | 3.022317 | LMO1 |
| ENSG00000188783 | ENSG00000188783 | -1.19293 | 10.62983 | -4.78885 | 2.43E-06 | 6.28E-05 | 4.408818 | PRELP |
| ENSG00000155511 | ENSG00000155511 | -1.19122 | 3.648957 | -5.80479 | 1.38E-08 | 9.54E-07 | 9.299476 | GRIA1 |
| ENSG00000170153 | ENSG00000170153 | -1.19048 | 9.015459 | -5.92577 | 7.10E-09 | 5.65E-07 | 9.934403 | RNF150 |
| ENSG00000145147 | ENSG00000145147 | -1.19021 | 8.703019 | -5.55426 | 5.32E-08 | 2.83E-06 | 8.019367 | SLIT2 |
| ENSG00000180139 | ENSG00000180139 | -1.18949 | 7.196934 | -6.971 | 1.45E-11 | 4.33E-09 | 15.85438 | ACTA2-AS1 |
| ENSG00000078549 | ENSG00000078549 | -1.1886 | 4.399901 | -5.22559 | 2.90E-07 | 1.14E-05 | 6.412627 | ADCYAP1R1 |
| ENSG00000004846 | ENSG00000004846 | -1.18788 | 3.445489 | -5.65274 | 3.15E-08 | 1.85E-06 | 8.516911 | ABCB5 |
| ENSG00000215386 | ENSG00000215386 | -1.18672 | 6.273904 | -6.0406 | 3.73E-09 | 3.47E-07 | 10.54702 | MIR99AHG |
| ENSG00000125851 | ENSG00000125851 | -1.1846 | 3.92484 | -4.50298 | 8.99E-06 | 0.000181 | 3.180684 | PCSK2 |
| ENSG00000188778 | ENSG00000188778 | -1.18412 | 3.156015 | -5.5042 | 6.93E-08 | 3.52E-06 | 7.769264 | ADRB3 |
| ENSG00000175497 | ENSG00000175497 | -1.18314 | 3.155625 | -4.28257 | 2.36E-05 | 0.000396 | 2.280011 | DPP10 |
| ENSG00000231768 | ENSG00000231768 | -1.18212 | 2.938625 | -6.49597 | 2.66E-10 | 4.39E-08 | 13.06963 | LINC01354 |
| ENSG00000165186 | ENSG00000165186 | -1.18187 | 5.737736 | -4.60193 | 5.76E-06 | 0.000126 | 3.598201 | PTCHD1 |
| ENSG00000171819 | ENSG00000171819 | -1.18101 | 4.398942 | -5.83623 | 1.16E-08 | 8.34E-07 | 9.463454 | ANGPTL7 |
| ENSG00000138615 | ENSG00000138615 | -1.17922 | 8.10822 | -4.19986 | 3.35E-05 | 0.000524 | 1.952577 | CILP |
| ENSG00000065534 | ENSG00000065534 | -1.17725 | 13.15571 | -5.92777 | 7.02E-09 | 5.62E-07 | 9.945016 | MYLK |
| ENSG00000091986 | ENSG00000091986 | -1.17033 | 12.23572 | -5.67157 | 2.85E-08 | 1.71E-06 | 8.61292 | CCDC80 |
| ENSG00000069431 | ENSG00000069431 | -1.16713 | 9.491698 | -6.71144 | 7.23E-11 | 1.63E-08 | 14.31385 | ABCC9 |
| ENSG00000150625 | ENSG00000150625 | -1.16669 | 4.966029 | -5.5203 | 6.37E-08 | 3.29E-06 | 7.849511 | GPM6A |
| ENSG00000155816 | ENSG00000155816 | -1.16528 | 4.96418 | -4.68207 | 3.99E-06 | 9.32E-05 | 3.942222 | FMN2 |
| ENSG00000108018 | ENSG00000108018 | -1.16452 | 4.858459 | -4.64046 | 4.83E-06 | 0.000109 | 3.762928 | SORCS1 |
| ENSG00000006128 | ENSG00000006128 | -1.16007 | 3.0144 | -3.89325 | 0.000117 | 0.001437 | 0.78948 | TAC1 |
| ENSG00000172399 | ENSG00000172399 | -1.15942 | 2.4917 | -6.72547 | 6.63E-11 | 1.52E-08 | 14.39601 | MYOZ2 |
| ENSG00000279192 | ENSG00000279192 | -1.15916 | 4.964512 | -5.6552 | 3.11E-08 | 1.83E-06 | 8.529434 | PWAR5 |
| ENSG00000188730 | ENSG00000188730 | -1.15521 | 3.475996 | -5.32851 | 1.72E-07 | 7.44E-06 | 6.90678 | VWC2 |
| ENSG00000013293 | ENSG00000013293 | -1.15247 | 3.151362 | -5.14613 | 4.32E-07 | 1.57E-05 | 6.036774 | SLC7A14 |
| ENSG00000149575 | ENSG00000149575 | -1.15207 | 4.323904 | -5.28887 | 2.11E-07 | 8.74E-06 | 6.715478 | SCN2B |
| ENSG00000102466 | ENSG00000102466 | -1.15029 | 6.014363 | -5.66445 | 2.96E-08 | 1.76E-06 | 8.576602 | FGF14 |
| ENSG00000080224 | ENSG00000080224 | -1.14915 | 3.189798 | -5.04651 | 7.06E-07 | 2.32E-05 | 5.572568 | EPHA6 |
| ENSG00000235601 | ENSG00000235601 | -1.1475 | 3.959645 | -5.27849 | 2.22E-07 | 9.17E-06 | 6.665585 | BARX1-DT |
| ENSG00000143196 | ENSG00000143196 | -1.13748 | 7.80948 | -5.2579 | 2.47E-07 | 1.00E-05 | 6.566862 | DPT |
| ENSG00000166923 | ENSG00000166923 | -1.13676 | 13.40556 | -5.00292 | 8.73E-07 | 2.74E-05 | 5.371984 | GREM1 |
| ENSG00000108231 | ENSG00000108231 | -1.13641 | 3.106696 | -5.27588 | 2.25E-07 | 9.27E-06 | 6.653045 | LGI1 |
| ENSG00000127951 | ENSG00000127951 | -1.13554 | 11.10462 | -6.79558 | 4.31E-11 | 1.07E-08 | 14.80831 | FGL2 |
| ENSG00000169760 | ENSG00000169760 | -1.13546 | 5.410393 | -4.74879 | 2.93E-06 | 7.34E-05 | 4.232658 | NLGN1 |
| ENSG00000152137 | ENSG00000152137 | -1.13378 | 9.725489 | -5.76745 | 1.70E-08 | 1.13E-06 | 9.105678 | HSPB8 |
| ENSG00000068781 | ENSG00000068781 | -1.13318 | 2.811838 | -6.53196 | 2.14E-10 | 3.75E-08 | 13.27523 | STON1-GTF2A1L |
| ENSG00000170500 | ENSG00000170500 | -1.12597 | 7.33469 | -4.36106 | 1.68E-05 | 0.000302 | 2.596083 | LONRF2 |
| ENSG00000165323 | ENSG00000165323 | -1.12528 | 5.972267 | -5.03165 | 7.59E-07 | 2.46E-05 | 5.504042 | FAT3 |
| ENSG00000214575 | ENSG00000214575 | -1.12427 | 5.328733 | -6.40676 | 4.51E-10 | 6.72E-08 | 12.5639 | CPEB1 |
| ENSG00000152583 | ENSG00000152583 | -1.12419 | 13.22514 | -6.7084 | 7.36E-11 | 1.65E-08 | 14.29608 | SPARCL1 |
| ENSG00000139910 | ENSG00000139910 | -1.12375 | 6.954273 | -5.43036 | 1.02E-07 | 4.77E-06 | 7.403868 | NOVA1 |
| ENSG00000243244 | ENSG00000243244 | -1.12323 | 9.227779 | -7.01802 | 1.08E-11 | 3.43E-09 | 16.13825 | STON1 |
| ENSG00000151892 | ENSG00000151892 | -1.12011 | 8.346433 | -4.18794 | 3.52E-05 | 0.000547 | 1.905823 | GFRA1 |
| ENSG00000259134 | ENSG00000259134 | -1.11805 | 4.79361 | -6.2614 | 1.06E-09 | 1.30E-07 | 11.7518 | LINC00924 |
| ENSG00000138722 | ENSG00000138722 | -1.11471 | 7.939432 | -5.29616 | 2.03E-07 | 8.51E-06 | 6.750545 | MMRN1 |
| ENSG00000198626 | ENSG00000198626 | -1.11407 | 7.981956 | -5.34345 | 1.60E-07 | 6.96E-06 | 6.979196 | RYR2 |
| ENSG00000173068 | ENSG00000173068 | -1.11303 | 9.125214 | -6.31091 | 7.91E-10 | 1.06E-07 | 12.02675 | BNC2 |
| ENSG00000154654 | ENSG00000154654 | -1.11184 | 6.074323 | -5.08714 | 5.78E-07 | 1.98E-05 | 5.760974 | NCAM2 |
| ENSG00000122824 | ENSG00000122824 | -1.10734 | 3.981786 | -5.59339 | 4.33E-08 | 2.38E-06 | 8.216169 | NUDT10 |
| ENSG00000167281 | ENSG00000167281 | -1.10729 | 4.813468 | -4.44204 | 1.18E-05 | 0.000225 | 2.927631 | RBFOX3 |
| ENSG00000082293 | ENSG00000082293 | -1.10728 | 4.451 | -4.76385 | 2.73E-06 | 6.93E-05 | 4.298732 | COL19A1 |
| ENSG00000249307 | ENSG00000249307 | -1.10642 | 4.320078 | -6.02994 | 3.96E-09 | 3.64E-07 | 10.48976 | LINC01088 |
| ENSG00000183454 | ENSG00000183454 | -1.10604 | 6.568616 | -4.38315 | 1.52E-05 | 0.000279 | 2.685974 | GRIN2A |
| ENSG00000078295 | ENSG00000078295 | -1.10275 | 6.91394 | -5.35008 | 1.54E-07 | 6.78E-06 | 7.011356 | ADCY2 |
| ENSG00000154258 | ENSG00000154258 | -1.10239 | 6.997338 | -5.74554 | 1.91E-08 | 1.24E-06 | 8.992477 | ABCA9 |
| ENSG00000082175 | ENSG00000082175 | -1.10117 | 6.87473 | -6.07947 | 3.00E-09 | 2.93E-07 | 10.75653 | PGR |
| ENSG00000181195 | ENSG00000181195 | -1.10007 | 4.088427 | -3.71007 | 0.000239 | 0.00255 | 0.133346 | PENK |
| ENSG00000250337 | ENSG00000250337 | -1.10007 | 4.275417 | -3.70647 | 0.000242 | 0.002578 | 0.120735 | PURPL |
| ENSG00000248485 | ENSG00000248485 | -1.09969 | 4.817289 | -4.01386 | 7.23E-05 | 0.000978 | 1.23739 | PCP4L1 |
| ENSG00000168356 | ENSG00000168356 | -1.0983 | 3.981015 | -6.23755 | 1.21E-09 | 1.45E-07 | 11.61995 | SCN11A |
| ENSG00000070193 | ENSG00000070193 | -1.09797 | 5.971103 | -4.57223 | 6.58E-06 | 0.000141 | 3.472016 | FGF10 |
| ENSG00000080644 | ENSG00000080644 | -1.09766 | 6.271523 | -4.44215 | 1.18E-05 | 0.000225 | 2.928056 | CHRNA3 |
| ENSG00000166573 | ENSG00000166573 | -1.0956 | 2.279207 | -5.93632 | 6.70E-09 | 5.41E-07 | 9.990267 | GALR1 |
| ENSG00000170381 | ENSG00000170381 | -1.09345 | 7.073732 | -3.84489 | 0.000142 | 0.001672 | 0.613444 | SEMA3E |
| ENSG00000115665 | ENSG00000115665 | -1.09095 | 2.783317 | -4.79547 | 2.35E-06 | 6.11E-05 | 4.438042 | SLC5A7 |
| ENSG00000121871 | ENSG00000121871 | -1.09077 | 3.13895 | -4.48365 | 9.79E-06 | 0.000194 | 3.100081 | SLITRK3 |
| ENSG00000106483 | ENSG00000106483 | -1.09036 | 11.03282 | -3.69256 | 0.000255 | 0.002685 | 0.072133 | SFRP4 |
| ENSG00000106034 | ENSG00000106034 | -1.09014 | 9.936502 | -6.06105 | 3.33E-09 | 3.16E-07 | 10.65709 | CPED1 |
| ENSG00000181092 | ENSG00000181092 | -1.08956 | 2.463716 | -3.61908 | 0.000337 | 0.00336 | -0.18171 | ADIPOQ |
| ENSG00000154175 | ENSG00000154175 | -1.08566 | 10.23672 | -5.09673 | 5.52E-07 | 1.91E-05 | 5.805621 | ABI3BP |
| ENSG00000179542 | ENSG00000179542 | -1.08475 | 4.872407 | -5.77926 | 1.59E-08 | 1.07E-06 | 9.166876 | SLITRK4 |
| ENSG00000171495 | ENSG00000171495 | -1.08457 | 2.208086 | -5.06602 | 6.42E-07 | 2.14E-05 | 5.662888 | MROH2B |
| ENSG00000178343 | ENSG00000178343 | -1.08447 | 6.333309 | -4.02314 | 6.96E-05 | 0.000948 | 1.27239 | SHISA3 |
| ENSG00000248309 | ENSG00000248309 | -1.08066 | 4.101547 | -6.44716 | 3.55E-10 | 5.52E-08 | 12.79226 | MEF2C-AS1 |
| ENSG00000104435 | ENSG00000104435 | -1.07875 | 5.423264 | -4.2014 | 3.33E-05 | 0.000521 | 1.958587 | STMN2 |
| ENSG00000179300 | ENSG00000179300 | -1.07685 | 2.751464 | -5.42785 | 1.03E-07 | 4.83E-06 | 7.391522 | RTL3 |
| ENSG00000151789 | ENSG00000151789 | -1.07632 | 6.710684 | -6.19078 | 1.59E-09 | 1.77E-07 | 11.36264 | ZNF385D |
| ENSG00000237179 | ENSG00000237179 | -1.07371 | 1.774842 | -6.66079 | 9.84E-11 | 2.03E-08 | 14.01852 | LINC01797 |
| ENSG00000095713 | ENSG00000095713 | -1.07315 | 4.91089 | -4.28874 | 2.29E-05 | 0.000389 | 2.30466 | CRTAC1 |
| ENSG00000120729 | ENSG00000120729 | -1.07229 | 4.773686 | -5.90678 | 7.89E-09 | 6.13E-07 | 9.834036 | MYOT |
| ENSG00000213088 | ENSG00000213088 | -1.07211 | 8.912032 | -4.33811 | 1.85E-05 | 0.000328 | 2.503148 | ACKR1 |
| ENSG00000174099 | ENSG00000174099 | -1.07174 | 10.57456 | -6.27599 | 9.70E-10 | 1.22E-07 | 11.83263 | MSRB3 |
| ENSG00000144229 | ENSG00000144229 | -1.06908 | 5.20336 | -4.3344 | 1.88E-05 | 0.000332 | 2.488157 | THSD7B |
| ENSG00000253647 | ENSG00000253647 | -1.06868 | 1.88802 | -6.7884 | 4.51E-11 | 1.10E-08 | 14.76593 | KCNIP1-OT1 |
| ENSG00000154553 | ENSG00000154553 | -1.06832 | 11.56178 | -6.0188 | 4.22E-09 | 3.82E-07 | 10.42997 | PDLIM3 |
| ENSG00000165566 | ENSG00000165566 | -1.06785 | 2.072301 | -6.30207 | 8.33E-10 | 1.10E-07 | 11.97749 | AMER2 |
| ENSG00000094963 | ENSG00000094963 | -1.06693 | 8.473847 | -4.70733 | 3.55E-06 | 8.53E-05 | 4.051754 | FMO2 |
| ENSG00000124212 | ENSG00000124212 | -1.06554 | 10.10652 | -4.22556 | 3.00E-05 | 0.000481 | 2.053689 | PTGIS |
| ENSG00000260802 | ENSG00000260802 | -1.06419 | 3.696088 | -4.71119 | 3.49E-06 | 8.40E-05 | 4.068558 | SERTM2 |
| ENSG00000149591 | ENSG00000149591 | -1.06386 | 13.52963 | -5.42648 | 1.04E-07 | 4.85E-06 | 7.38479 | TAGLN |
| ENSG00000106819 | ENSG00000106819 | -1.06283 | 11.17982 | -5.64583 | 3.27E-08 | 1.89E-06 | 8.481772 | ASPN |
| ENSG00000224957 | ENSG00000224957 | -1.06088 | 2.968434 | -5.84304 | 1.12E-08 | 8.13E-07 | 9.499025 | LINC01266 |
| ENSG00000223561 | ENSG00000223561 | -1.06069 | 4.242379 | -4.45901 | 1.09E-05 | 0.000212 | 2.99777 | LOC646588 |
| ENSG00000175857 | ENSG00000175857 | -1.05955 | 6.795099 | -5.47249 | 8.18E-08 | 4.01E-06 | 7.611829 | GAPT |
| ENSG00000274956 | ENSG00000274956 | -1.05904 | 3.711994 | -4.382 | 1.53E-05 | 0.00028 | 2.681293 | NKAIN3-IT1 |
| ENSG00000185046 | ENSG00000185046 | -1.05701 | 5.39199 | -5.65166 | 3.17E-08 | 1.86E-06 | 8.511446 | ANKS1B |
| ENSG00000102468 | ENSG00000102468 | -1.05475 | 5.983675 | -5.31856 | 1.81E-07 | 7.79E-06 | 6.858651 | HTR2A |
| ENSG00000075035 | ENSG00000075035 | -1.05125 | 5.886705 | -3.75661 | 0.0002 | 0.002211 | 0.297286 | WSCD2 |
| ENSG00000258537 | ENSG00000258537 | -1.05091 | 1.517112 | -6.57855 | 1.62E-10 | 3.03E-08 | 13.54272 | FRMD6-AS2 |
| ENSG00000128591 | ENSG00000128591 | -1.0506 | 11.7767 | -3.97524 | 8.45E-05 | 0.001109 | 1.092616 | FLNC |
| ENSG00000236548 | ENSG00000236548 | -1.05056 | 3.240652 | -6.73135 | 6.40E-11 | 1.48E-08 | 14.43042 | RNF217-AS1 |
| ENSG00000119938 | ENSG00000119938 | -1.05055 | 7.700416 | -5.31999 | 1.80E-07 | 7.74E-06 | 6.865537 | PPP1R3C |
| ENSG00000144057 | ENSG00000144057 | -1.05049 | 5.711997 | -4.65682 | 4.48E-06 | 0.000103 | 3.833278 | ST6GAL2 |
| ENSG00000243069 | ENSG00000243069 | -1.05041 | 3.662349 | -4.97142 | 1.02E-06 | 3.11E-05 | 5.227939 | ARHGEF26-AS1 |
| ENSG00000145936 | ENSG00000145936 | -1.05037 | 9.27697 | -5.42846 | 1.03E-07 | 4.82E-06 | 7.394529 | KCNMB1 |
| ENSG00000182132 | ENSG00000182132 | -1.04933 | 4.457169 | -5.30727 | 1.92E-07 | 8.15E-06 | 6.804085 | KCNIP1 |
| ENSG00000180287 | ENSG00000180287 | -1.04782 | 2.970622 | -4.49671 | 9.24E-06 | 0.000185 | 3.154527 | PLD5 |
| ENSG00000166250 | ENSG00000166250 | -1.04753 | 9.833029 | -5.9816 | 5.20E-09 | 4.48E-07 | 10.23102 | CLMP |
| ENSG00000150627 | ENSG00000150627 | -1.04739 | 5.339352 | -4.70604 | 3.57E-06 | 8.56E-05 | 4.04616 | WDR17 |
| ENSG00000102271 | ENSG00000102271 | -1.04717 | 5.429946 | -5.96434 | 5.73E-09 | 4.80E-07 | 10.1391 | KLHL4 |
| ENSG00000109846 | ENSG00000109846 | -1.046 | 9.508462 | -5.94196 | 6.49E-09 | 5.30E-07 | 10.02019 | CRYAB |
| ENSG00000118407 | ENSG00000118407 | -1.04511 | 8.998021 | -6.2496 | 1.13E-09 | 1.37E-07 | 11.68652 | FILIP1 |
| ENSG00000154262 | ENSG00000154262 | -1.04491 | 7.652078 | -6.24517 | 1.16E-09 | 1.39E-07 | 11.66204 | ABCA6 |
| ENSG00000141052 | ENSG00000141052 | -1.04342 | 8.680127 | -4.5772 | 6.44E-06 | 0.000138 | 3.493096 | MYOCD |
| ENSG00000232046 | ENSG00000232046 | -1.04088 | 2.793417 | -6.32659 | 7.22E-10 | 9.80E-08 | 12.11415 | LINC01798 |
| ENSG00000164530 | ENSG00000164530 | -1.0404 | 6.444331 | -3.68581 | 0.000262 | 0.002743 | 0.048649 | PI16 |
| ENSG00000203808 | ENSG00000203808 | -1.038 | 2.937425 | -5.24199 | 2.67E-07 | 1.06E-05 | 6.49078 | BVES-AS1 |
| ENSG00000158270 | ENSG00000158270 | -1.03752 | 8.852955 | -5.83844 | 1.15E-08 | 8.29E-07 | 9.474985 | COLEC12 |
| ENSG00000004799 | ENSG00000004799 | -1.03738 | 11.12397 | -4.94772 | 1.14E-06 | 3.41E-05 | 5.120068 | PDK4 |
| ENSG00000248994 | ENSG00000248994 | -1.03704 | 1.817512 | -6.20496 | 1.46E-09 | 1.66E-07 | 11.44048 | LOC105374618 |
| ENSG00000197614 | ENSG00000197614 | -1.03466 | 9.436333 | -4.71293 | 3.46E-06 | 8.35E-05 | 4.076118 | MFAP5 |
| ENSG00000072133 | ENSG00000072133 | -1.03438 | 5.555196 | -4.57785 | 6.42E-06 | 0.000138 | 3.495868 | RPS6KA6 |
| ENSG00000107796 | ENSG00000107796 | -1.03421 | 13.81683 | -6.20311 | 1.48E-09 | 1.68E-07 | 11.43033 | ACTA2 |
| ENSG00000149451 | ENSG00000149451 | -1.03157 | 7.920983 | -4.49497 | 9.31E-06 | 0.000186 | 3.14727 | ADAM33 |
| ENSG00000148948 | ENSG00000148948 | -1.03147 | 4.830295 | -5.38727 | 1.27E-07 | 5.77E-06 | 7.192589 | LRRC4C |
| ENSG00000164188 | ENSG00000164188 | -1.0307 | 3.908568 | -5.90455 | 7.99E-09 | 6.19E-07 | 9.822244 | RANBP3L |
| ENSG00000102383 | ENSG00000102383 | -1.03029 | 5.627836 | -6.02324 | 4.12E-09 | 3.76E-07 | 10.45377 | ZDHHC15 |
| ENSG00000223403 | ENSG00000223403 | -1.02972 | 2.719787 | -6.79314 | 4.38E-11 | 1.08E-08 | 14.79392 | MEG9 |
| ENSG00000111404 | ENSG00000111404 | -1.02932 | 4.824599 | -4.63517 | 4.95E-06 | 0.000111 | 3.740245 | RERGL |
| ENSG00000232079 | ENSG00000232079 | -1.02892 | 3.481417 | -5.08031 | 5.98E-07 | 2.03E-05 | 5.729211 | LINC01697 |
| ENSG00000168702 | ENSG00000168702 | -1.02796 | 3.794827 | -5.16705 | 3.89E-07 | 1.45E-05 | 6.135213 | LRP1B |
| ENSG00000109132 | ENSG00000109132 | -1.02794 | 1.673886 | -5.20909 | 3.15E-07 | 1.21E-05 | 6.334163 | PHOX2B |
| ENSG00000054938 | ENSG00000054938 | -1.02777 | 9.646294 | -3.70662 | 0.000242 | 0.002577 | 0.121251 | CHRDL2 |
| ENSG00000266524 | ENSG00000266524 | -1.02686 | 4.124545 | -4.11577 | 4.76E-05 | 0.0007 | 1.625596 | GDF10 |
| ENSG00000132639 | ENSG00000132639 | -1.02654 | 6.051237 | -3.95086 | 9.32E-05 | 0.0012 | 1.001856 | SNAP25 |
| ENSG00000132554 | ENSG00000132554 | -1.02557 | 4.073227 | -5.6589 | 3.05E-08 | 1.80E-06 | 8.548288 | RGS22 |
| ENSG00000130822 | ENSG00000130822 | -1.02514 | 6.119204 | -3.8585 | 0.000135 | 0.001604 | 0.662767 | PNCK |
| ENSG00000171246 | ENSG00000171246 | -1.02391 | 6.045513 | -3.45049 | 0.000624 | 0.005473 | -0.74613 | NPTX1 |
| ENSG00000153002 | ENSG00000153002 | -1.02379 | 3.122361 | -4.32222 | 1.99E-05 | 0.000347 | 2.43904 | CPB1 |
| ENSG00000108924 | ENSG00000108924 | -1.02353 | 7.690941 | -5.40027 | 1.19E-07 | 5.46E-06 | 7.256158 | HLF |
| ENSG00000053328 | ENSG00000053328 | -1.02325 | 5.500943 | -4.6078 | 5.60E-06 | 0.000123 | 3.623219 | METTL24 |
| ENSG00000151320 | ENSG00000151320 | -1.02299 | 8.106501 | -5.44283 | 9.55E-08 | 4.52E-06 | 7.465275 | AKAP6 |
| ENSG00000136267 | ENSG00000136267 | -1.02291 | 4.450299 | -4.94726 | 1.14E-06 | 3.41E-05 | 5.117972 | DGKB |
| ENSG00000140538 | ENSG00000140538 | -1.0228 | 5.576438 | -4.74546 | 2.97E-06 | 7.42E-05 | 4.218097 | NTRK3 |
| ENSG00000134548 | ENSG00000134548 | -1.02075 | 2.970045 | -5.34874 | 1.55E-07 | 6.81E-06 | 7.004884 | SPX |
| ENSG00000112964 | ENSG00000112964 | -1.01976 | 7.360812 | -5.91383 | 7.59E-09 | 5.97E-07 | 9.871275 | GHR |
| ENSG00000189320 | ENSG00000189320 | -1.01792 | 6.142215 | -4.97764 | 9.87E-07 | 3.04E-05 | 5.256322 | FAM180A |
| ENSG00000182050 | ENSG00000182050 | -1.01725 | 3.361692 | -4.62116 | 5.27E-06 | 0.000118 | 3.680268 | MGAT4C |
| ENSG00000144191 | ENSG00000144191 | -1.01442 | 3.785954 | -4.92149 | 1.29E-06 | 3.77E-05 | 5.001255 | CNGA3 |
| ENSG00000188906 | ENSG00000188906 | -1.01383 | 8.765342 | -6.37326 | 5.49E-10 | 7.89E-08 | 12.37541 | LRRK2 |
| ENSG00000229558 | ENSG00000229558 | -1.01186 | 1.808091 | -5.72199 | 2.17E-08 | 1.38E-06 | 8.871178 | SACS-AS1 |
| ENSG00000132872 | ENSG00000132872 | -1.01131 | 2.887103 | -3.72989 | 0.000221 | 0.002401 | 0.202934 | SYT4 |
| ENSG00000145242 | ENSG00000145242 | -1.01096 | 3.230424 | -5.38328 | 1.30E-07 | 5.86E-06 | 7.173103 | EPHA5 |
| ENSG00000163637 | ENSG00000163637 | -1.01083 | 9.40236 | -6.49132 | 2.73E-10 | 4.49E-08 | 13.04318 | PRICKLE2 |
| ENSG00000163395 | ENSG00000163395 | -1.0094 | 4.26617 | -3.92417 | 0.000104 | 0.001303 | 0.903118 | IGFN1 |
| ENSG00000100448 | ENSG00000100448 | -1.00907 | 4.434345 | -3.8954 | 0.000116 | 0.001427 | 0.797369 | CTSG |
| ENSG00000091656 | ENSG00000091656 | -1.00889 | 8.485362 | -5.10415 | 5.32E-07 | 1.85E-05 | 5.840198 | ZFHX4 |
| ENSG00000172915 | ENSG00000172915 | -1.0078 | 8.393507 | -4.69503 | 3.76E-06 | 8.90E-05 | 3.998375 | NBEA |
| ENSG00000152092 | ENSG00000152092 | -1.00719 | 4.079663 | -3.84408 | 0.000142 | 0.001676 | 0.610502 | ASTN1 |
| ENSG00000126010 | ENSG00000126010 | -1.00671 | 4.082997 | -4.59335 | 5.98E-06 | 0.00013 | 3.561678 | GRPR |
| ENSG00000203685 | ENSG00000203685 | -1.00658 | 6.425239 | -3.84496 | 0.000142 | 0.001672 | 0.613702 | STUM |
| ENSG00000249923 | ENSG00000249923 | -1.00551 | 3.933136 | -4.12048 | 4.67E-05 | 0.000688 | 1.643751 | LOC284865 |
| ENSG00000068976 | ENSG00000068976 | -1.00457 | 6.64744 | -5.66215 | 3.00E-08 | 1.78E-06 | 8.564854 | PYGM |
| ENSG00000170624 | ENSG00000170624 | -1.00281 | 9.749385 | -5.97618 | 5.36E-09 | 4.55E-07 | 10.20215 | SGCD |

Table S4. Lasso Coefficients

| ENSEMBL | coef | gene_id | Primer-F | Primer-R |  |
| --- | --- | --- | --- | --- | --- |
| ENSG00000198523 | -0.47595 | PLN | ACCTCACTCGCTCAGCTATAA | CATCACGATGATACAGATCAGCA | |
| ENSG00000150893 | -0.30834 | FREM2 | CCTGCATGACCTGGTGTTG | GCCAGTGCGTCGTTGTCTA | |
| ENSG00000260230 | -0.21375 | FRRS1L | CCTGTCGGAGGAGGGTTAC | CCACAGTCGTCCACTTTGATT | |
| ENSG00000138207 | -0.20879 | RBP4 | AGGAGAACTTCGACAAGGCTC | GAGAACTCCGCGACGATGTT | |
| ENSG00000115593 | -0.19652 | SMYD1 | AGAACGTGGAGGTCTTCACC | AAACCACTGCGGAATAAGCCC | |
| ENSG00000088386 | -0.17606 | SLC15A1 | TCTTTGGTTATCCCCTGAGCA | GGCGGTGGACAGGTTATCATC | |
| ENSG00000175161 | -0.17173 | CADM2 | AAACTTCCAAGGCATATCTCACC | TGCGATTTGCATCCTCTTCTT | |
| ENSG00000138650 | -0.16938 | PCDH10 | TGGATGGTGGAAGGAGTCTTT | TTCAGCGATATTCCCCACGAA | |
| ENSG00000187772 | -0.1578 | LIN28B | CATCTCCATGATAAACCGAGAGG | GTTACCCGTATTGACTCAAGGC | |
| ENSG00000182256 | 0.154487 | GABRG3 | GTGGGTCTTGGCTCCAAAATC | ACGTCAATTACGGTCGGTTTTA | |
| ENSG00000004776 | 0.158091 | HSPB6 | TGCTAGACGTGAAGCACTTCT | ACCACCTTGACAGCAATTTCC | |
| ENSG00000236824 | 0.16821 | BCYRN1 | GGAGCCCCAATAGAGCTTATACA | TCCACACCTACACCATACATCC | |
| ENSG00000165966 | 0.183082 | PDZRN4 | CCAGCACTCAGACGGACATC | CTAACACGACACAACTCGACC | |
| ENSG00000018625 | 0.187599 | ATP1A2 | GGCCGCAAATACCAAGTGGA | CCACACCCAGATATAGATTGTCG | |
| ENSG00000184905 | 0.211787 | TCEAL2 | GGAGGATCAAAGACAGAGGGA | CTCCACCCTAGCCATGTTGT | |
| ENSG00000091482 | 0.257107 | SMPX | ACAGCCAGTTTCCAATGTTAGAG | CTGTTCAGCTTTGGGGACATATT | |
| ENSG00000146469 | 0.261418 | VIP | GACACCAGAAATAAGGCCCAG | GTCACCCAACCTGAGAGCAG | |
| ENSG00000136546 | 0.387002 | SCN7A | CAATGCGGCTTCCATCTTGTG | ACGCAATCAATCAGGACACTAAT | |
